# Design, synthesis, in vitro anti-α-glucosidase evaluations, and computational studies of new phthalimide-phenoxy-1,2,3-triazole-*N*-phenyl (or benzyl) acetamides as potential anti-diabetic agents

**DOI:** 10.1038/s41598-023-36890-y

**Published:** 2023-06-20

**Authors:** Mehdi Emadi, Mohammad Halimi, Ali Moazzam, Samanesadat Hosseini, Somayeh Mojtabavi, Mohammad Ali Faramarzi, Reza Ghadimi, Ali Akbar Moghadamnia, Ensieh Nasli-Esfahani, Maryam Mohammadi-Khanaposhtani, Mohammad Mahdavi

**Affiliations:** 1 Electrical and Computer Engineering Department, Babol Noshirvani University of Technology, Babol, Iran; 2 Department of Biology, Babol Branch, Islamic Azad University, Babol, Iran; 3 Endocrinology and Metabolism Research Center, Endocrinology and Metabolism Clinical Sciences Institute, Tehran University of Medical Sciences, Tehran, Iran; 4 Shahid Beheshti University of Medical Sciences, Tehran, Iran; 5 Department of Pharmaceutical Biotechnology, Faculty of Pharmacy, Tehran University of Medical Sciences, Tehran, Iran; 6 Social Determinants of Health Research Center, Health Research Institute, Babol University of Medical Sciences, Babol, Iran; 7 Department of Pharmacology and Toxicology, School of Medicine, Babol University of Medical Sciences, Babol, Iran; 8 Cellular and Molecular Biology Research Center, Health Research Institute, Babol University of Medical Sciences, Babol, Iran; 9 Diabetes Research Center, Endocrinology and Metabolism Clinical Sciences Institute, Tehran University of Medical Sciences, Tehran, Iran

**Keywords:** Computational biology and bioinformatics, Drug discovery

## Abstract

An important target in the treatment of type 2 diabetes is α-glucosidase. Inhibition of this enzyme led to delay in glucose absorption and decrease in postprandial hyperglycemia. A new series of phthalimide-phenoxy-1,2,3-triazole-*N*-phenyl (or benzyl) acetamides **11a**–**n** were designed based on the reported potent α-glucosidase inhibitors. These compounds were synthesized and screened for their in vitro inhibitory activity against the latter enzyme. The majority of the evaluated compounds displayed high inhibition effects (IC_50_ values in the range of 45.26 ± 0.03–491.68 ± 0.11 µM) as compared to the positive control acarbose (IC_50_ value = 750.1 ± 0.23 µM). Among this series, compounds **11j** and **11i** represented the most potent α-glucosidase inhibitory activities with IC_50_ values of 45.26 ± 0.03 and 46.25 ± 0.89 µM. Kinetic analysis revealed that the compound **11j** is a competitive inhibitor with a K_i_ of 50.4 µM. Furthermore, the binding interactions of the most potent compounds in α-glucosidase active site were studied through molecular docking and molecular dynamics. The latter studies confirmed the obtained results through in vitro experiments. Furthermore, in silico pharmacokinetic study of the most potent compounds was also performed.

## Introduction

Glucosidases are an important group of digestive enzymes that are responsible for the hydrolytic cleavage of glycosidic bonds of oligosaccharides and disaccharides^1^. The catalytic specificity of glucosidases depends on the position of cleavage site, the configuration of the hydroxyl groups, and the number of monosaccharides in substrate^2^. Two main types of glucosidases are α-glucosidases and β-glucosidases which differ in the orientation of carboxylic acid residues during catalysis process^3^. Among the latter enzymes, α-glucosidase (EC 3.2.1.20) has received a special attention to the pharmaceutical researchers because of its catalytic activity in the intestine that led to the delay in glucose absorption and the decrease in postprandial hyperglycemia^4^. Therefore, inhibition of this enzyme is an important protocol in treatment of type 2 diabetes mellitus (T2DM) as the most widespread chronic metabolic disorder related to hyperglycemia^5^. Acarbose and other available α-glucosidase inhibitors cause various side effects including abdominal discomfort, bloating, diarrhea, pain, and flatulence^6^. On the other hand, α-glucosidase has also been well appreciated as a valuable therapeutic target for other carbohydrate related diseases including viral infections, cancer, and hepatitis^7–9^. Therefore, development of safe and efficient α-glucosidase inhibitors for treating T2DM and other carbohydrat-related diseases is an important goal for pharmacists.

**Figure 1:**
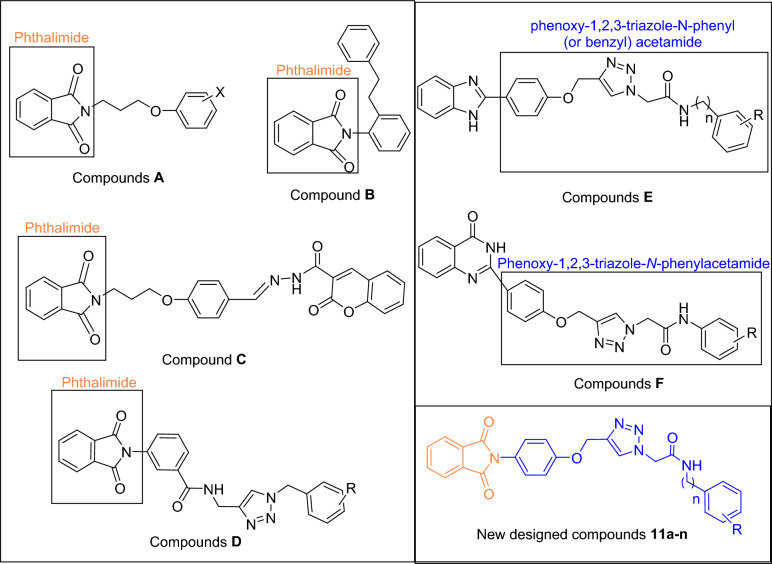
Design strategy for phthalimide-phenoxy-1,2,3-triazole-*N*-phenyl (or benzyl) acetamide derivatives **11a–n** as new α-glucosidase inhibitors.

One of the most important heterocycles in the design of new potent synthetic α-glucosidase inhibitors is phthalimide^10,11^. As can be seen Fig. 1, compounds **A**–**D** are derivatives of phthalimide that exhibited high inhibitory activity against α-glucosidase^12–15^. On the other hand, several series of phenoxy-1,2,3-triazole-*N*-phenyl (or benzyl) acetamide derivatives with significant anti-α-glucosidase activity have been reported (Fig. 1, compounds **E**–**F**)^16,17^. Therefore, we concluded that the combination of phthalimide ring and phenoxy-1,2,3-triazole-*N*-phenyl (or benzyl) acetamide moiety can lead to new potent α-glucosidase inhibitors. In this regards, phthalimide-phenoxy-1,2,3-triazole-*N*-phenyl (or benzyl) acetamide scaffold designed and fourteen derivatives **11a**–**n** of this scaffold were synthesized and evaluated against α-glucosidase in vitro and in silico.

## Results and discussion

### Chemistry

**Scheme 1:**
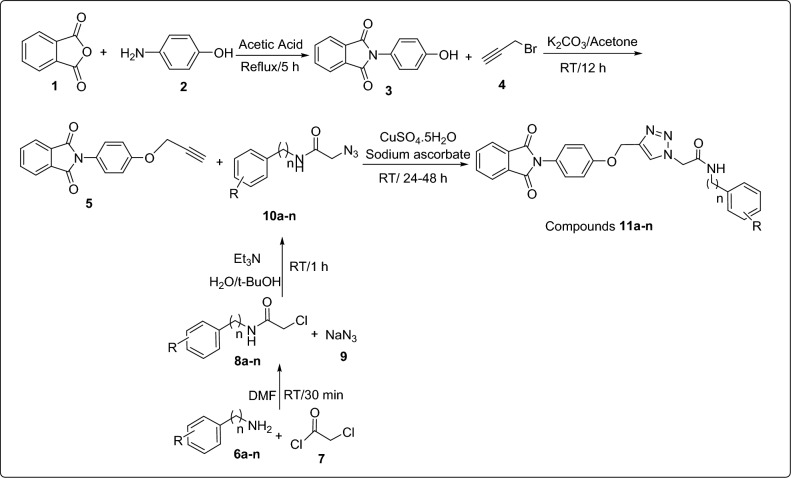
Synthesis of phthalimide-phenoxy-1,2,3-triazole-*N*-phenyl (or benzyl) acetamide derivatives **11a**–**n**.

The synthetic route for the synthesis of phthalimide-phenoxy-1,2,3-triazole-*N*-phenyl (or benzyl) acetamides **11a**–**n** has been depicted in Scheme 1. This route was started from the reaction of phthalic anhydride **1** and 4-aminophenol **2** in acetic acid at reflux condition to give 2-(4-hydroxyphenyl)isoindoline-1,3-dione **3**. Then, compound** 3** was reacted with propargyl bromide** 4** in the presence of potassium carbonate in acetone and afforded 2-(4-(prop-2-yn-1-yloxy)phenyl)isoindoline-1,3-dione** 5**. On the other hand, amine derivatives **6a**–**n** were reacted with chloroacetyl chloride **7** in DMF at RT for 30 min to give *N*-phenyl-2-chloroacetamides **8a**–**n**. The latter compounds and sodium azide **9** were reacted in the mixture of H_2_O/t-BuOH (1:1) in the presence of triethylamine (Et_3_N) at RT for 1 h to give azide derivatives **10a**–**n**. Finally, mixture of 2-(4-(prop-2-yn-1-yloxy)phenyl)isoindoline-1,3-dione** 5**, sodium ascorbate, and copper(II) sulfate (CuSO_4_) was added to the freshly prepared azide derivatives **10a**–**n** and the reaction was continued at RT for 24–48 h to give the target compounds **11a**–**n**.

### In vitro*** inhibitory activity of compounds 11a***–***n against α-glucosidase***

The in vitro enzymatic inhibitory activity of phthalimide-phenoxy-1,2,3-triazole-*N*-phenyl (or benzyl) acetamide **11a**–**n** was evaluated against yeast α-glucosidase. Acarbose was chosen as positive control and anti-α-glucosidase activities of *N*-phenylacetamide derivatives **11a**–**l** and *N*-benzylacetamide derivatives **11m–n** are expressed as IC_50_ values in Table 1.

**Table 1:** Anti-α-glucosidase activity of new phthalimide derivatives **11a**–**n** in comparison with positive control acarbose.


Compound	R	n	IC_50_ (µM)^a^
**11a**	H	0	203.78 ± 0.32
**11b**	2,3-Dimethyl	0	366.10 ± 0.86
**11c**	2,6-Dimethyl	0	750 <
**11d**	4-Ethyl	0	491.68 ± 0.11
**11e**	3-F	0	188.35 ± 0.27
**11f**	3-Cl	0	112.16 ± 0.03
**11g**	2,3-Dichloro	0	239.10 ± 0.41
**11h**	2,4-Dichloro	0	283.98 ± 0.41
**11i**	2,6-Dichloro	0	46.25 ± 0.89
**11j**	4-Br	0	45.26 ± 0.03
**11k**	4-Nitro	0	750 <
**11l**	2-Methyl-4-nitro	0	273.29 ± 0.28
**11m**	H	1	750 <
**11n**	4-F	1	685.99 ± 0.32
Acarbose	-	-	750.1 ± 0.23

^a^IC_50_ values are indicated as the mean ± SD of three independent experiments.

### Structure–activity relationship (SAR) for α-glucosidase inhibitory activity

As evidenced by obtained results, all of the *N*-phenylacetamide derivatives, with the exception of 2,6-dimethyl derivative **11c** and 4-nitro derivative **11j**, were more potent than positive control acarbose. In contrast, *N*-benzylacetamide derivatives **11m**–**n** did not show significant inhibitory activity against this enzyme.

In the *N*-phenylacetamide derivatives **9a**–**l**, the most potent compound was 4-bromo derivative **11j**. Substitution of bromine with nitro group, in case of compound **11k**, leads to the complete loss of inhibitory activity and substitution of bromine with ethyl group, in case of compound **11d**, leads to a significant decrease in the inhibitory activity.

The second most potent compound was 2,6-dichloro derivative **11i**. 2,3-Dichloro derivative **11g** and 2,4-dichloro derivative **11j** as regioisomers of compound **11i** showed moderate anti-α-glucisidase activity in comparison to this compound. The third potent compound among the newly synthesized compounds was 3-chloro derivative **11f**. Replacing of chloro substituent with fluorine atom, as in case of compound **11e** (the fourth potent compound), caused to a moderate decrease in the anti-α-glucosidase activity. The fifth potent compound was un-substituted compound **11a**. Placing two methyl substituents on the pendant phenyl group of compound **11a** depending on their positions, has interesting effects on the inhibitory effect of this compound: 2,3-dimethyl derivative **11b** was 1.8-fold less potent than compound **11a** while 2,6-dimethyl derivative **11c** was inactive. Another interesting point that can be observed about the effect of methyl substitution in the obtained inhibitory activities is that the 4-nitro derivative is an inactive compound, but by adding a methyl on 2-position of the 4-nitrophenyl group, potent compound **11l** is obtained.

As can be seen in Table 1, *N*-benzylacetamide derivatives **11m**–**n** did not show a significant inhibitory against α-glucosidase.

### Comparison of the new compounds 11 with template compounds E and F

**Scheme 2:**
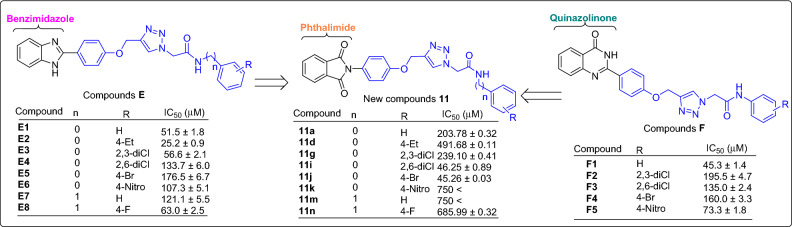
Comparison of IC_50_ values of phthalimide derivatives **11** against α-glucosidase with their corresponding analogs of benzimidazole derivatives **E** and quinazolinone derivatives **F**.

The comparison of IC_50_ values of the new phthalimide derivatives **11** with their corresponding analogs of benzimidazole derivatives **E** revealed that with the exception of 2,6-dichloro and 4-bromo derivatives, benzimidazole analogs were more potent than their corresponding analogs of phthalimide series (Scheme 2).The mentioned trend is also observed in the comparison between phthalimide derivatives **11** and quinazolinone derivatives **F** (Scheme 2)^17^.

### Kinetic study

**Figure 2:**
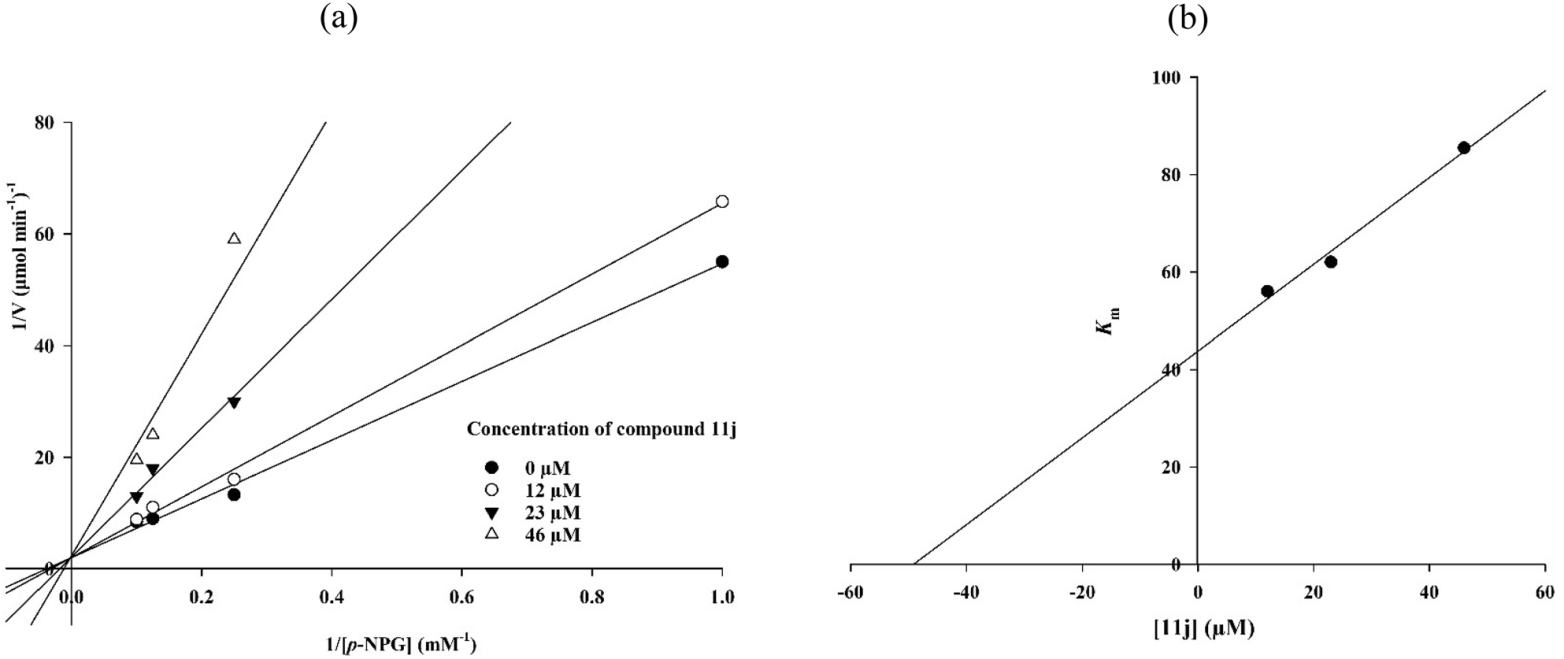
(**a**) Lineweaver–Burk plot of the kinetics of α-glucosidase inhibition by **11j**. (**b**) Secondary re-plot of Lineweaver–Burk plots between the slopes of each line on Lineweaver–Burk plot versus various concentrations of **11j**.

To gain further insight into the mechanism of α-glucosidase inhibition of the title class of compounds, a kinetic study was performed on compound **11j** as the most potent α-glucosidase inhibitor. For this purpose, the reaction rates in the presence of different concentrations of compound **11j** were measured in various concentrations of substrate (*p*-nitrophenyl-a-D-glucopyranoside). Graphs of different concentrations of inhibitor were drawn by the Lineweaver–Burk plot (Fig. 2a). As the concentrations of inhibitors increased, V_max_ values were not affected, but K_m_ values gradually decreased, thereby indicating that compound **11j** was a competitive inhibitor against α-glucosidase (Fig. 2a). The K_i_ value was calculated directly by plotting the slope of each line in the Lineweaver–Burk plots into the different concentrations of compound **11j** (Fig. 2b). The results proved that K_i_ value of compound **11j** was 50.4 µM.

### Molecular docking study

**Figure 3:**
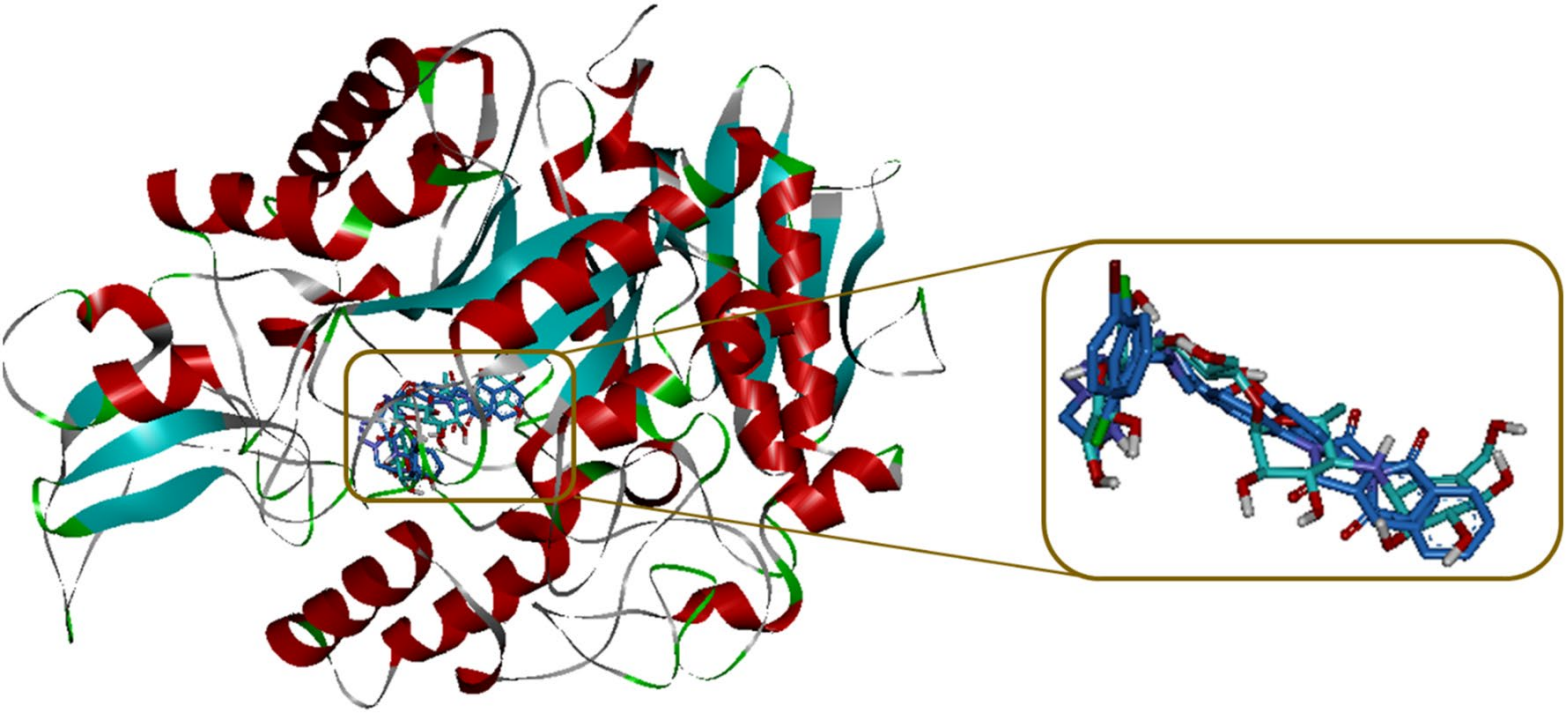
Acarbose (cyan) and the most potent compounds **11j**, **11i**, and **11f.** (blue) superimposed in the α-glucosidase active site.

In order to explain interactions of the most potent compounds **11j**, **11i**, and **11f.** in α-glucosidase active site, molecular docking simulation was carried out^18^. The superposed structure of acarbose and the title new compounds in the active site of target enzyme is shown in Fig. 3. Interaction modes of the positive control acarbose and selected compounds **11j**, **11i**, and **11f.** were showed in the Fig. 3 and details of their interactions in the active site of target enzyme were listed in Table 2.

**Table 2:** Interaction mode details of the compounds **11j**, **11i**, and **11f**.

Compound	Interaction	Interacting unit of the ligand	Amino acid
**11j**	π-anion	Phthalimide	Glu304
	Hydrophobic	Phthalimide	Pro309
	Hydrophobic	Phthalimide	Pro309
	H-bond	O atom of phenoxy	Asn241
	π-cation	Phenyl ring of phenoxy	His279
	H-bond	1,2,3-Triazole	His239
	π-cation	1,2,3-Triazole	His239
	H-bond	C = O unit of acetamide	Arg312
	Hydrophobic	4-Br of *N*-phenylacetamide	Phe158
	Hydrophobic	Phenyl ring of* N*-phenylacetamide	Arg312
**11i**	Hydrophobic	Phthalimide	Val305
	Hydrophobic	Phthalimide	Pro309
	Hydrophobic	Phenyl ring of phenoxy	Pro309
	H-bond	1,2,3-Triazole	Asn241
	π-cation	1,2,3-Triazole	His279
	Hydrophobic	1,2,3-Triazole	His239
	Non-classical H-bond	C = O unit of acetamide	Phe157
	H-bond	NH unit of acetamide	Phe157
	Hydrophobic	2-Cl of *N*-phenylacetamide	Phe311
	Hydrophobic	2-Cl of *N*-phenylacetamide	Tyr313
	Hydrophobic	2-Cl of *N*-phenylacetamide	Arg312
	Hydrophobic	6-Cl of *N*-phenylacetamide	Phe157
	π-anion	Phenyl ring of* N*-phenylacetamide	Asp408
	Hydrophobic	Phenyl ring of* N*-phenylacetamide	Arg312
**11f.**	Hydrophobic	Phthalimide	Pro309
	Hydrophobic	Phthalimide	Pro309
	π-anion	Phenyl ring of phenoxy	Glu304
	Non-classical H-bond	1,2,3-Triazole	Phe311
	Non-classical H-bond	1,2,3-Triazole	Arg312
	H-bond	C=O unit of acetamide	Asn241
	Unfavorable	C=O unit of acetamide	Asp408
	H-bond	NH unit of acetamide	Asp408
	H-bond	NH unit of acetamide	Phe157

**Figure 4:**
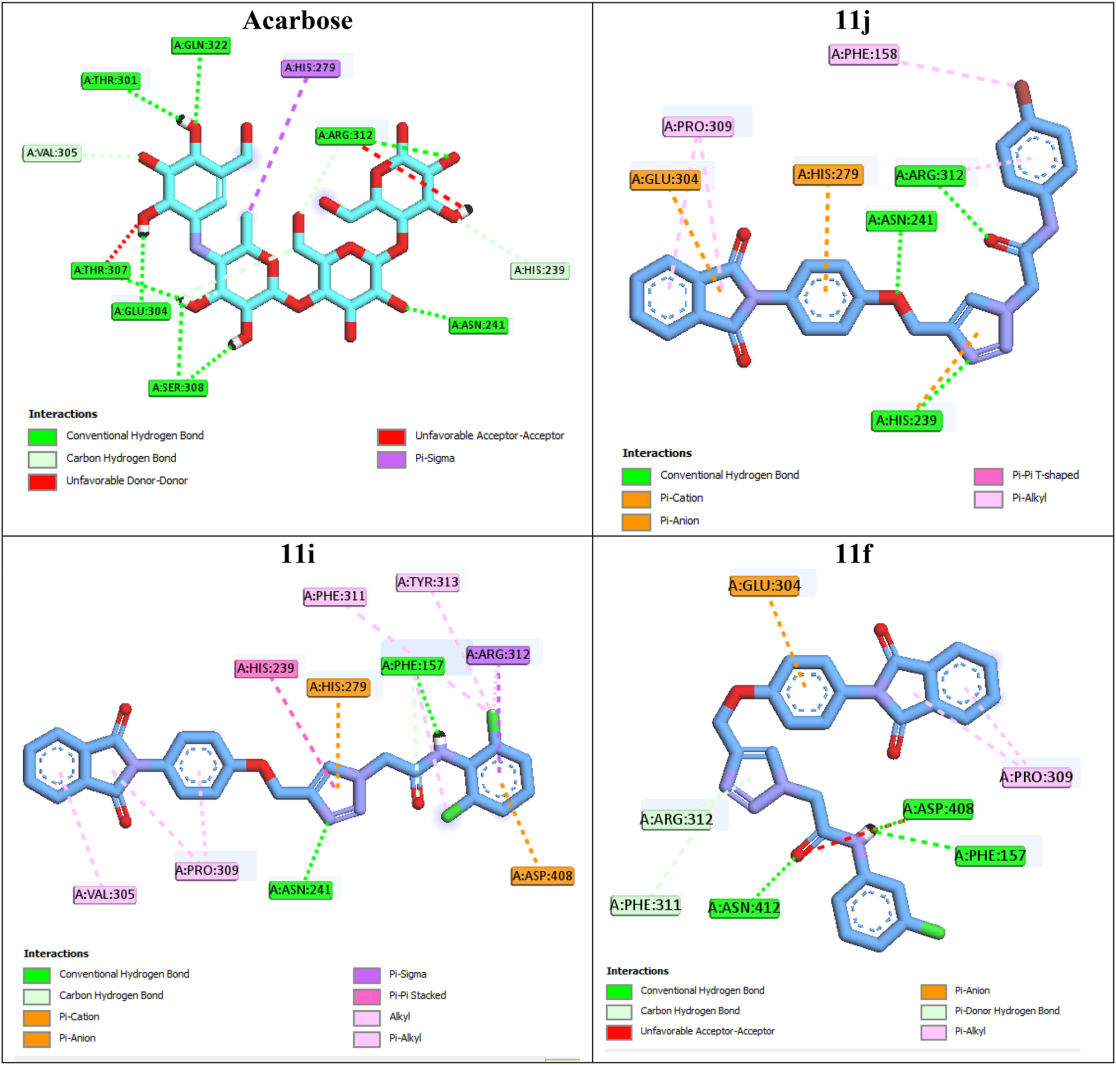
2D interaction modes of the positive control acarbose and the most potent compounds **11j**, **11i**, and **11f** in the α-glucosidase active site.

The 2D interaction mode of acarbose demonstrated that this positive control created eight hydrogen bonds with active site residues Thr307, Asn241, Glu304, Pro309, Ser308, Thr301, Arg312, and Gln322 (Fig. 4). Acarbose also formed a hydrophobic interaction with residue His279, two non-classical hydrogen bonds with residues Val305 and His239, and two unfavorable interactions with residues Thr307 and Arg312. Moreover, BE of acarbose was -4.04 kcal/mol.

The most potent compound **11j** created three hydrogen bonds with residues Asn241, His239, and Arg312 via oxygen atom of phenoxy moiety, 1,2,3-triazole ring, and carbonyl unit of acetamide moiety, respectively (Fig. 4). Phthalimide moiety of this compound established three interaction with active site: a π-anion interaction with Glu304 and two hydrophobic interactions with Pro309. Two π-cation interactions were also observed between phenyl ring of phenoxy moiety with residue His279 and 1,2,3-triazole ring with His239. 4-Bromophenyl ring of compound 11j established two hydrophobic interactions with Phe158 and Arg312 via bromo substituent and phenyl ring, respectively. The second potent compound **11i** established two hydrogen bonds with residues Asn241 and Phe157 via 1,2,3-triazole ring and NH unit of acetamide moiety, respectively (Fig. 4). The latter amino acid also interacted with carbonyl unit of acatamide moiety via a non-classical hydrogen bond. Compound **11i** formed a π-cation interaction and a π-anion interaction with residues His279 and Asp408. This compound also established several hydrophobic interactions with residues Val305, Pro309, His239, Phe311, Tyr313, Arg312, and Phe157.

The third potent compound **11f.** created three classical hydrogen bonds with residues Asn241 and Asp408 via acetamide moiety and two non-classical hydrogen bonds with residues Phe311 and Arg312 via 1,2,3-triazole ring (Fig. 4). Phenyl ring of phenoxy moiety established a π-anion interaction with Glu304. Compound **11f.** also formed an unfavorable interaction with residue Asp408 via carbonyl unit of acetamide moiety.

**Figure 5:**
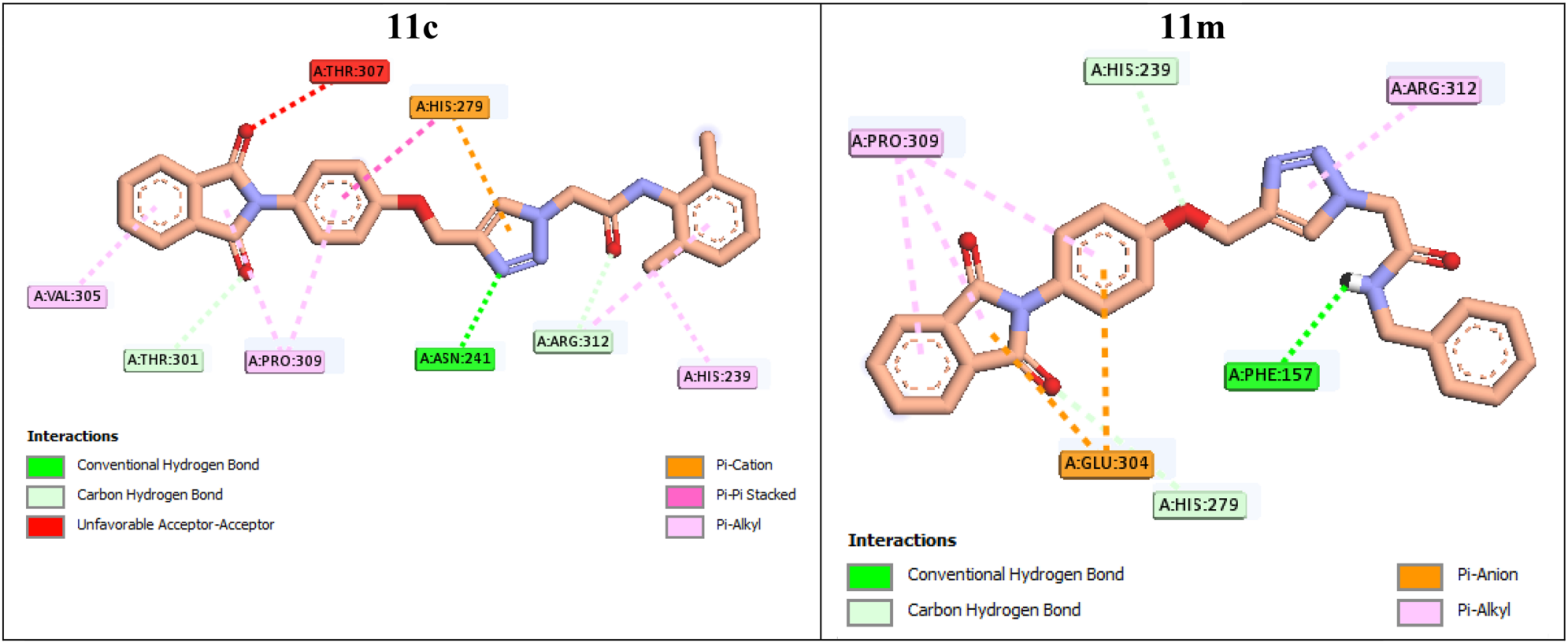
2D interaction modes of the inactive compounds **11c** and **11 m** in the α-glucosidase active site.

Docking study on the inactive compounds **11c** and **11m** showed that this compounds formed only a hydrogen bond with the active site of target enzyme. As can be seen in Fig. 5, compound **11c** formed a hydrogen bond with Asn241, a π-cation interaction with His279, two non-classical hydrogen bonds with Thr301 and Arg312, an unfavorable interaction with Thr307, and several hydrophobic interactions with Val305, His239, His279, Pro309, and Arg312. Inactive compound **11m** established a hydrogen bond with Phe157, two π-anion interactions with Glu304, two non-classical hydrogen bonds with His239 and His279, and several hydrophobic interactions with Pro309 and Arg312.

### Molecular dynamics

Interaction of a ligand with a protein is a dynamic phenomenon which takes place on a very small time scale. Molecular dynamic simulation helps to grasp the interaction between ligand and protein and evaluate the stability and flexibility of the resulting complex. To this end, dynamics of the protein–ligand complex is simulated in an environment very similar to the natural environment that includes water and ions. Based on in vitro studies compound **11j** has the most potential to inhibit α-glucosidase. Therefore the complex of α-glucosidase-**11j** was simulated in an explicit hydration environment by molecular dynamics simulation to evaluate the stability, flexibility and intermolecular interactions between α-glucosidase and this compound^19^. Moreover molecular dynamics of acarbose as a standard inhibitor in complex with α-glucosidase was simulated in an explicit hydration environment to have a decent reference for comparison. In this study, molecular dynamic simulation was performed in two steps. A 10 ns simulation at the first step to investigate if the ligands i.e. acarbose and **11j** were stable at their binding site on α-glucosidase. After confirming the stability of ligands in their binding site, simulation time was extended for another 10 ns to gain a better comprehension of the behavior of these compounds in the active site of α-glucosidase. Stability of **11j** and α-glucosidase was confirmed in this step too. For further evaluation the trajectory file was analyzed by several tools. To evaluate the stability of the complexes, root-mean-square deviation (RMSD) and radius of gyration (Rg) of all structures of the trajectory were calculated and the related graphs were drawn. To assess the residual flexibility and the flexibility of ligand atoms, the root mean square fluctuation (RMSF) of backbone atoms of α-glucosidase and heavy atoms of ligands were calculated.

**Figure 6:**
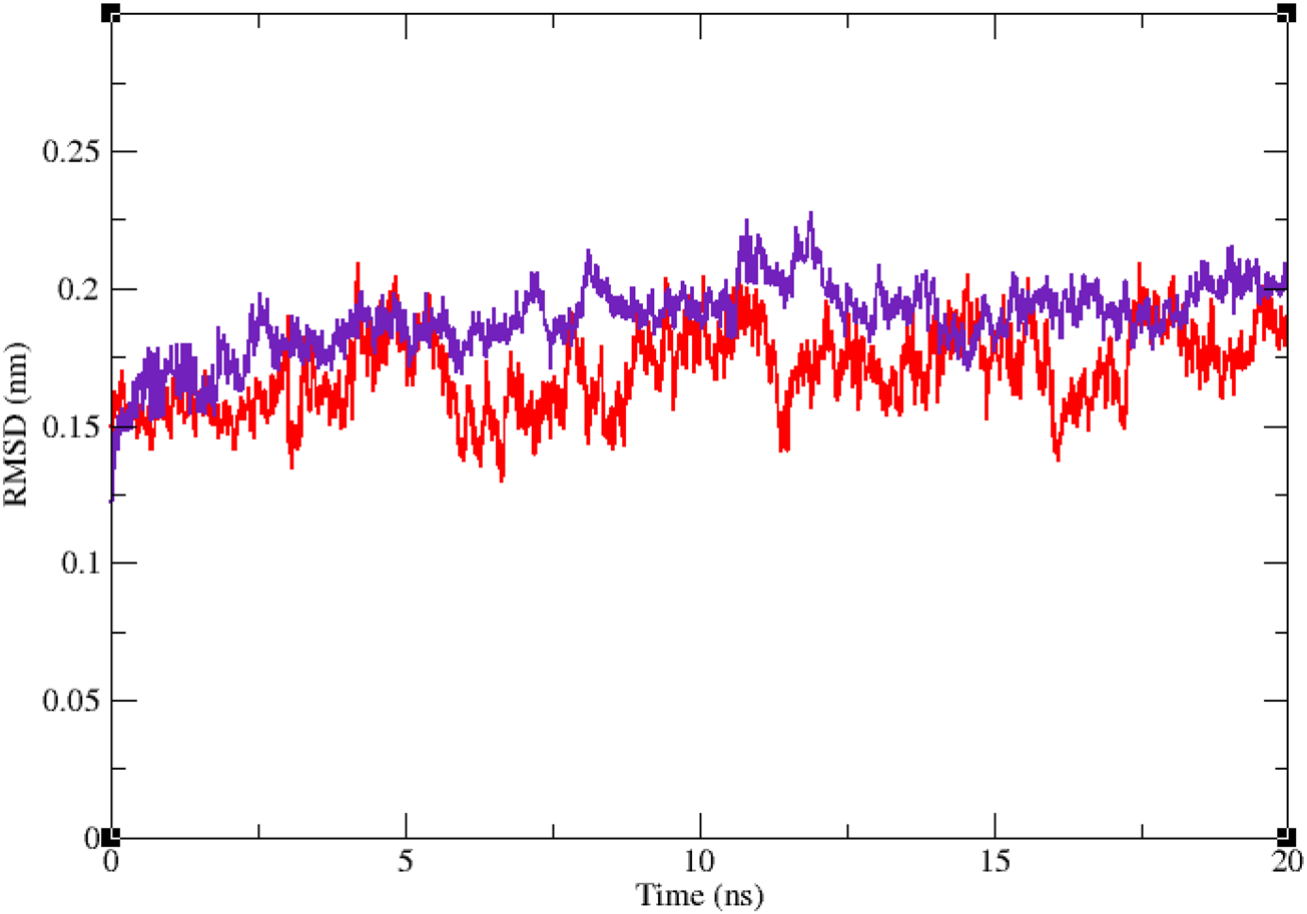
Superimposed RMSD of Cα atoms of α-glucosidase in complex with **11j** (red) and acarbose (indigo).

**Figure 7:**
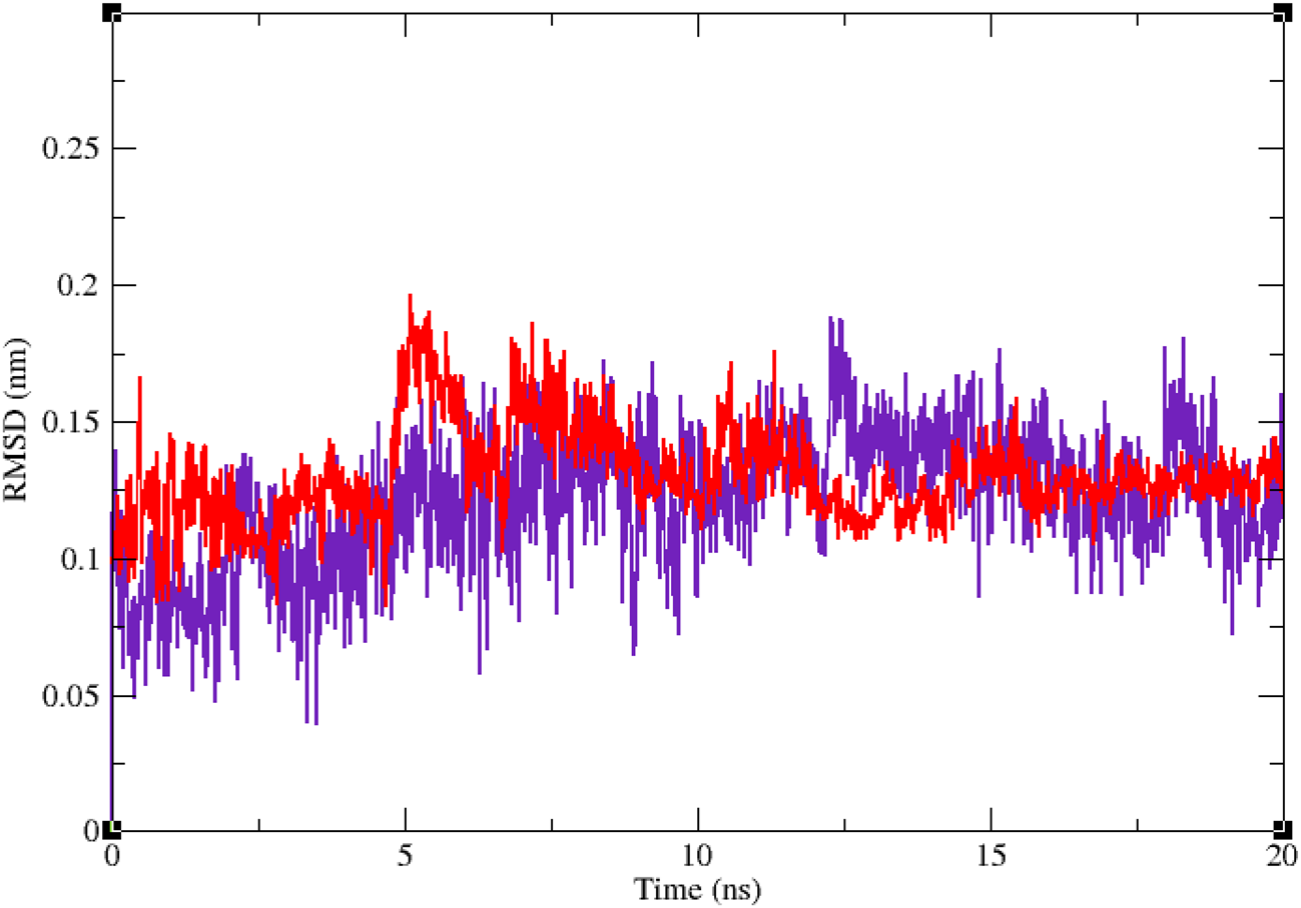
Superimposed RMSD of **11j** (red) and acarbose (indigo) in complex with α-glucosidase.

Figures 6 and 7 show the result of RMSD calculations. According to Fig. 6 that shows the RMSD of backbone atoms of α-glucosidase in complex with **11j** and acarbose, RMSD of α-glucosidase does not change very much and is less than 0.25 nm in all the simulation trajectory. RMSD less than 0.3 nm indicates a stable structure. The average RMSD values of α-glucosidase in the complex with acarbose and/or **11j** were 0.17 and 0.16 nm, respectively. RMSD less than 0.25 nm was observed for acarbose and/or **11j** in the complex with α-glucosidase too that shows they had the least conformational changes and were completely stable during the simulation time (Fig. 7). The average RMSD values of acarbose and/or **11j** in complexes with α-glucosidase were 0.14 and 0.14 nm, respectively.

**Figure 8:**
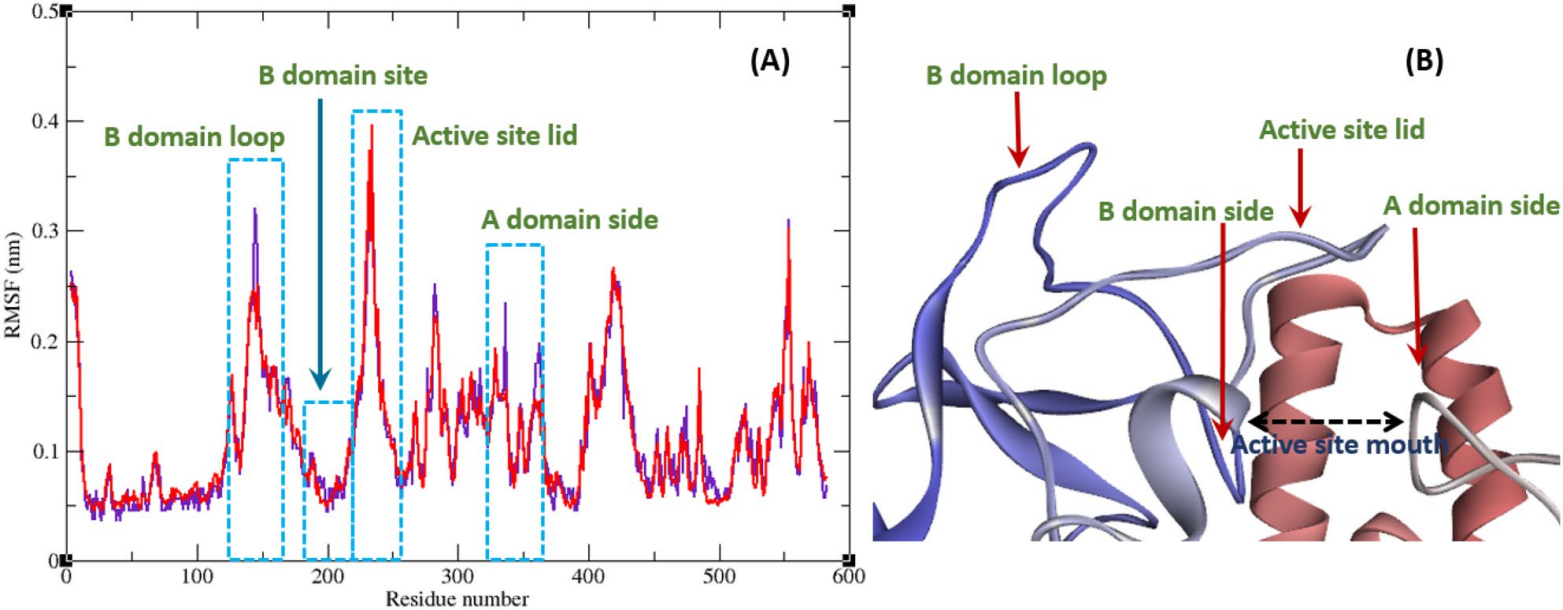
(**A**) RMSF graph of the Cα atoms of α-glucosidase in complex with acarbose (indigo) and **11j** (red). (**B**) Close-up representation of α-glucosidase active site.

**Figure 9:**
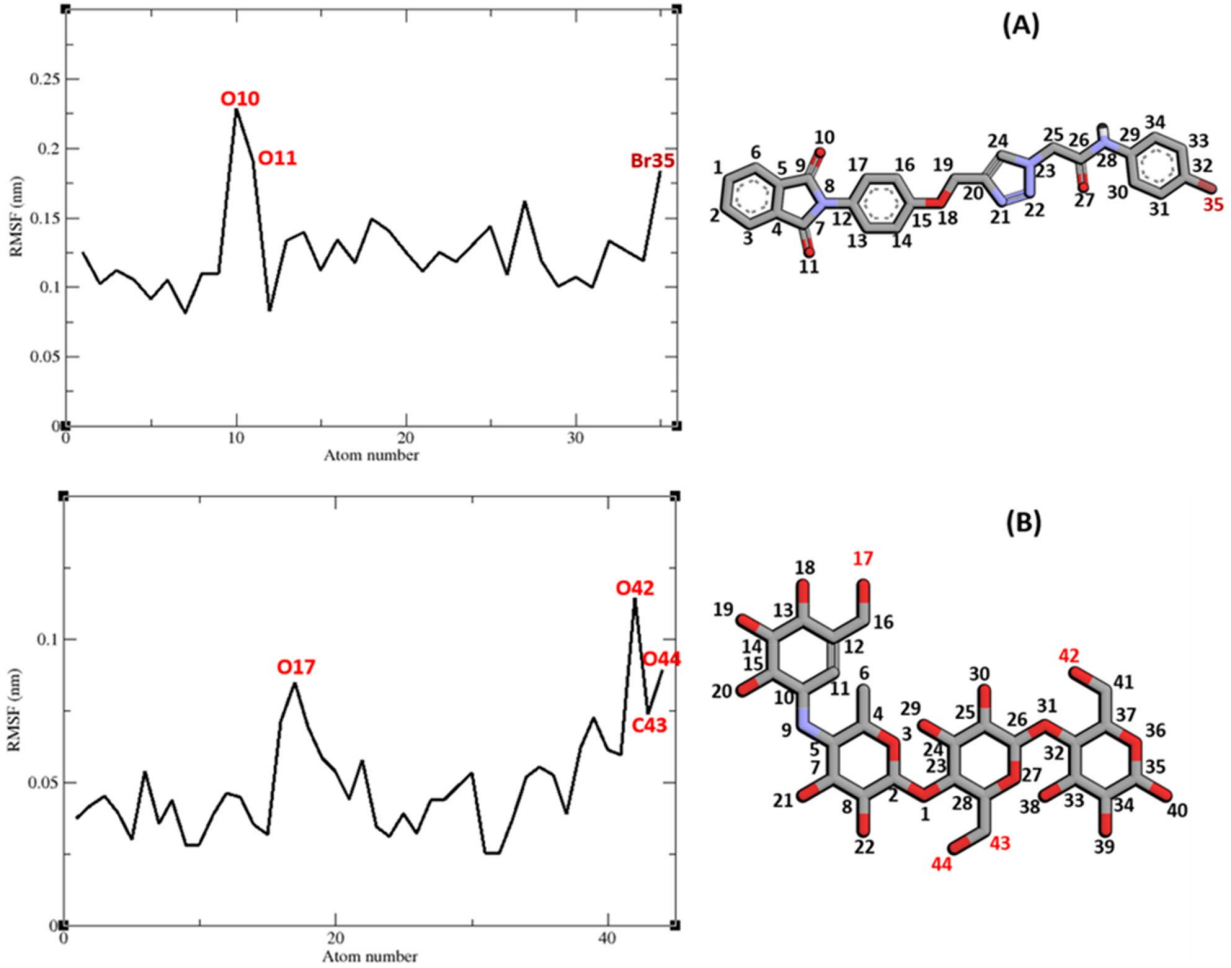
RMSF graph of the heavy atoms of **11j** (**A**) and acarbose (**B**) in complex with α-glucosidase. Structure of these compounds and parts of these molecules with greatest fluctuations are illustrated.

**Figure 10:**
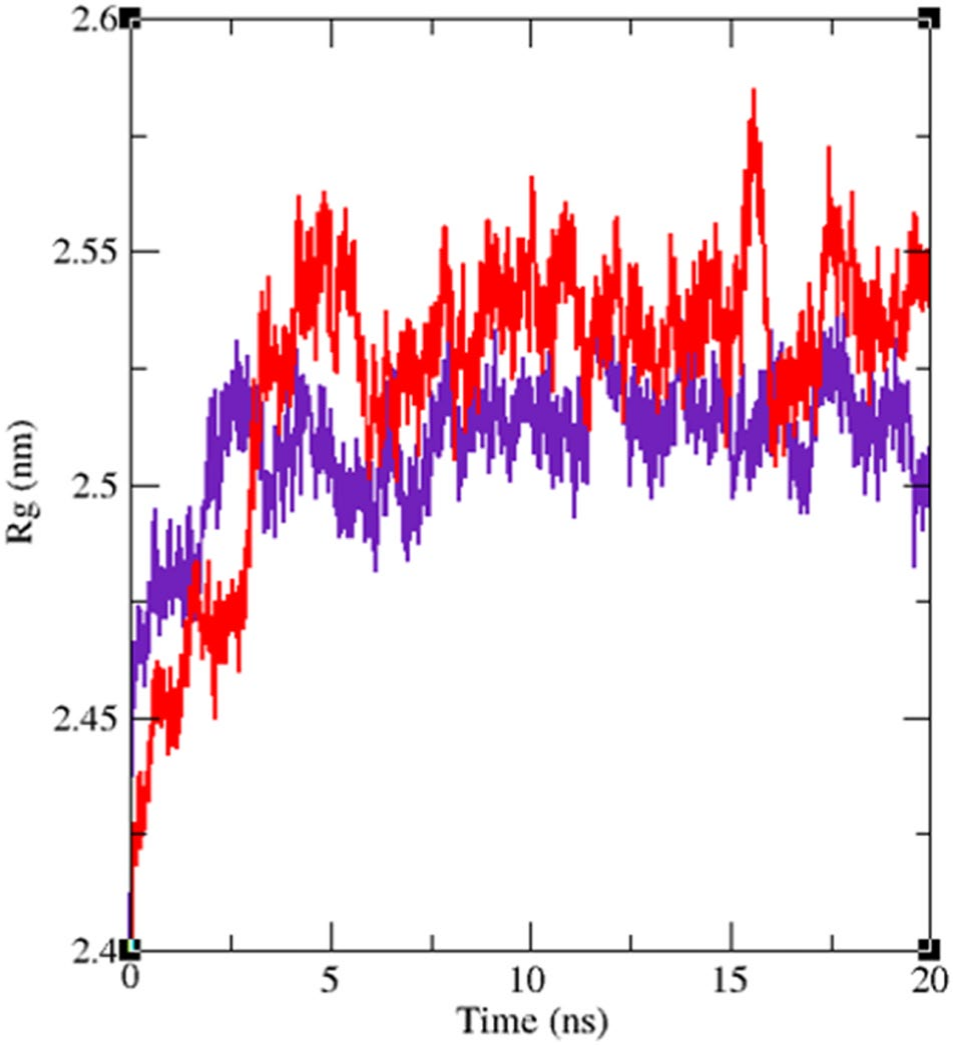
Time dependence of the radius of gyration (Rg) graph of α-glucosidase in complex with **11j** (red) and acarbose (indigo).

All atoms and consequently residues in a protein have some fluctuations. The fluctuation of α-glucosidase residues in complexes with acarbose and **11j** are depicted in Fig. 8. According to this figure RMSF of α-glucosidase residues in the complex of this enzyme with acarbose and/or **11j** are very similar and almost match. α-Glucosidase is a big protein with 579 residues and several domains with different structure and functions. As it could be seen from Fig. 8 the fluctuation of different parts of this big protein are not the same. Active site of this enzyme is located in a cleft between two domains i.e. A domain and B domain and the residues of these domains that contribute to the non-bond interactions with ligands have the lowest fluctuations. On the other hand, as is to be expected, residues located in the loop regions including B domain loop and active site lid have more fluctuations. RMSF of heavy atoms of acarbose and **11j** are depicted in Fig. 9. As can be seen in this figure, the RMSF of all heavy atoms in these ligands is less than 0.2 nm. This low RMSF indicates that these compounds have a stable structure in complex with α-glucosidase and intermolecular interactions limit their fluctuations. Among the heavy atoms of acarbose and **11j** those that were part of a ring had the lowest RMSF. Rings usually have less fluctuations as atoms of the ring limit their movements and make stable non-bond interactions such as π-Anion, π-Cation, π-Alkyl, π-π T-shaped, π-sigma, and hydrogen bonds with binding site residues of the protein. The radius of gyration (Rg) of α-glucosidase was calculated for evaluation of protein compactness during simulation (Fig. 10). The average Rg of α-glucosidase was 2.530 and 2.54 nm in the complex of α-glucosidase with acarbose and **11j**, respectively. The Rg value of α-glucosidase in complexes with both acarbose and **11j** was only in the narrow range of 2.45 to 2.52 nm and did not show a significant upward or downward trend during the simulation time that shows stable protein structures.

### In silico* pharmacokinetic and toxicity studies*

Pharmacokinetic (ADME) and toxicity predictions of the positive control acarbose and the most potent compounds **11j** and **11i** were performed by PreADMET online software (Table 3). As can be seen in Table 3, acarbos did not follow of Lipinski 'Rule of five' while compounds **11j** and **11i** followed of this rule. Acarbose and compounds **11j** and **11i** had poor permeability to Caco-2. Permeability to blood brain barrier (BBB) and skin for the title compounds is in the acceptable range. Compounds **11j** and **11i** had high human intestinal absorption (HIA) while acarbose did not have HIA. Prediction of mutagenicity of acarbose and compounds **11j** and **11i** demonstrated that these compounds are mutagen. Moreover, this study predicted that acarbose had carcinogenic effect on mouse and did not have this effect on rat while compound **11j** has carcinogenic effect on rat and mouse. Unlike the compound **11j,** compound **11i** has not carcinogenicity on the latter animals. Cardiotoxicity (hERG inhibition) of the positive acarbose is ambiguous while compounds **11j**and **11i** in term of this type of toxicity have medium risk.

**Table 3:** Pharmacokinetic (ADME) and toxicity prediction of acarbose and the most potent compounds **11j** and **11i**.

Druglikeness/ADME/T ^a^	Compound		
	Acarbose	**11j**	**11i**
Rule of Five	Violated	Suitable	Suitable
Caco2	9.44448	21.4585	21.2303
HIA	0.000000	96.936969	97.002824
BBB	0.0271005	0.117396	0.0829309
Skin permeability	−5.17615	−3.23477	−3.26244
Ames test	Mutagen	Mutagen	Mutagen
Carcino mouse	Positive	Positive	Negative
Carcino rat	Negative	Positive	Negative
hERG inhibition	Ambiguous	Medium risk	Medium risk

^a^The recommended ranges for Caco2: < 25 poor, > 500 great, HIA: > 80% is high < 25% is poor, BBB = −3.0 to 1.2, and Skin_Permeability = −8.0 to −1.0

## Conclusion

In conclusion, we designed and synthesized a novel series of phthalimide-phenoxy-1,2,3-triazole-*N*-phenyl (or benzyl) acetamide **11a**–**n**. The synthesized compounds **11a**–**n** were evaluated against α-glucosidase because were designed based on active pharmacophores of potent reported α-glucosidase inhibitors. The majority of the title compounds displayed high α-glucosidase inhibitory activity. Among them, compounds **11j** and **11i** represented the most potent α-glucosidase inhibitory activities with IC_50_ values of 45.26 ± 0.03 and 46.25 ± 0.89 µM as compared to the positive control acarbose (IC_50_ = 750.1 ± 0.23 µM). In addition, kinetic analysis demonstrated that compound **11j** behaves as a competitive inhibitor with a K_i_ value of 50.4 µM. The binding interactions of the most potent compounds and two inactive compounds at α-glucosidase active site were studied through molecular docking. The obtained results revealed that potent compounds formed significant interactions with the active site in comparison to inactive compounds. Molecular dynamics study of the most potent compound **11j** and positive control acarbose also demonstrated that our new compound showed an appropriate state in the α-glucosidase active site. Furthermore, in silico pharmacokinetic study predicted that the most potent new compounds **11j** and **11i** have satisfactory pharmacokinetics after the oral admission as drug candidates.

## Experimental

### General

Melting points of compounds **11a**–**n** were measured with a Kofler hot stage apparatus. IR spectra of these compounds were recorded with a Nicolet Magna FTIR 550 spectrophotometer (KBr disks). ^1^H and ^13^C NMR spectra of the title phthalimide derivatives were obtained with a Bruker FT-400 (TMS was used as an internal standard). Mass spectrometry results were obtained with an Agilent Technology (HP) mass spectrometer (Ionization potential: 70 eV). Elemental analysis was determined with an Elementar Analysen system GmbH VarioEL CHNS mode.

### Synthesis of 2-(4-hydroxyphenyl)isoindoline-1,3-dione 3

A mixture of phthalic anhydride **1** (10 mmol) and 4-aminophenol **2** (10 mmol) in acetic acid (50 mL) was stirred at reflux condition for 5 h. Then, cold water was added to the reaction mixture and appeared participates were separated by filtration to give pure 2-(4-hydroxyphenyl)isoindoline-1,3-dione **3.**

### Synthesis of 2-(4-(prop-2-yn-1-yloxy)phenyl)isoindoline-1,3-dione 5

A mixture of 2-(4-hydroxyphenyl)isoindoline-1,3-dione **3** (1 mmol), propargyl bromide **4** (1.2 mmol, 0.15 mL), and K_2_CO_3_ (1.2 mmol) in acetone (10 mL) was stirred at RT for 12 h. After completion of the reaction (checked by TLC), the reaction mixture poured into cold water. Subsequently, the precipitated product was filtered off to give pure 2-(4-(prop-2-yn-1-yloxy)phenyl)isoindoline-1,3-dione **5**.

### General procedure for the synthesis of azide derivatives 10a–n

Azide derivatives **10a**–**n** were synthesized in situ according to our pervious reported work^16^.

### General procedure for the synthesis of phthalimide-phenoxy-1,2,3-triazole-N-phenyl (or benzyl) acetamide 11a–n

A mixture of 2-(4-(prop-2-yn-1-yloxy)phenyl)isoindoline-1,3-dione **5** (1 mmol), sodium ascorbate, and CuSO_4_.5H_2_O (7 mol %) was added to the prepared azide derivatives **10a**–**n**, and the reaction mixture was stirred at RT for 24–48 h. After that, the reaction mixture was poured into crushed ice and appeared participates **11a**–**n** were filtered off, washed with cold water, and purified by recrystallization in ethyl acetate.

### 2-(4-((4-(1,3-dioxoisoindolin-2-yl)phenoxy)methyl)-1H-1,2,3-triazol-1-yl)-N-phenylacetamide (11a)

Yield: 77%. White crystal. M.p. 218–220 °C. IR (KBr): 3428, 1680, 1367, 1198 cm^−1^. ^1^H NMR (400 MHz, DMSO-*d*_6_) *δ* 10.59 (s, 1H), 8.34 (s, 1H), 7.92 (ddt, *J* = 20.0, 5.5, 3.2 Hz, 4H), 7.62 (dd, *J* = 8.8, 4.9 Hz, 2H), 7.39 (d, *J* = 8.4 Hz, 2H), 7.33–7.10 (m, 5H), 5.38 (s, 2H), 5.26 (s, 2H). ^13^C NMR (100 MHz, DMSO-*d*_6_) *δ* 167.72, 164.59, 158.11, 143.06, 135.25, 135.06, 132.03, 129.24, 126.96, 125.17, 123.79, 121.54, 121.46, 115.87, 115.34, 61.71, 52.66 ppm. EI-MS (70 eV): m/z (%) = 453.2 (M^+^). Anal. Calcd for C_25_H_19_N_5_O_4_: C 66.22; H 4.22; N 15.44; Found: C 66.46; H 4.38; N 15.68.

### N-(2,3-dimethylphenyl)-2-(4-((4-(1,3-dioxoisoindolin-2-yl)phenoxy)methyl)-1H-1,2,3-triazol-1-yl)acetamide (11b)

Yield: 85%. White crystal. M.p. 206–208 °C. IR (KBr): 3432, 1681, 1336, 1185 cm^−1^. ^1^H NMR (400 MHz, DMSO-*d*_6_)* δ* 9.92 (s, 1H), 8.33 (s, 1H), 7.93 (ddt, *J* = 21.1, 5.5, 3.2 Hz, 4H), 7.52–7.27 (m, 2H), 7.30–7.12 (m, 3H), 7.14–6.91 (m, 2H), 5.44 (s, 2H), 5.26 (s, 2H), 2.26 (s, 3H), 2.13 (s, 3H). ^13^C NMR (100 MHz, DMSO-*d*_6_) *δ* 167.71, 164.90, 158.13, 142.80, 137.63, 135.72, 135.07, 132.03, 131.51, 129.24, 127.75, 126.87, 125.78, 125.17, 123.79, 123.73, 115.34, 61.72, 52.41, 20.61, 14.50 ppm. EI-MS (70 eV): m/z (%) = 481.2 (M^+^). Anal. Calcd for C_27_H_23_N_5_O_4_: C 67.35; H 4.81; N 14.54; Found: C 67.46; H 14.69; N 14.68.

### N-(2,6-dimethylphenyl)-2-(4-((4-(1,3-dioxoisoindolin-2-yl)phenoxy)methyl)-1H-1,2,3-triazol-1-yl)acetamide (11c)

Yield: 88%. White crystal. M.p. 209–211 °C. IR (KBr): 3429, 1681, 1359, 1210 cm^−1^. ^1^H NMR (400 MHz, DMSO-*d*_6_) *δ* 9.83 (s, 1H), 8.33 (s, 1H), 7.93 (ddd, *J* = 24.9, 5.5, 3.1 Hz, 4H), 7.45–7.26 (m, 2H), 7.28–7.14 (m, 2H), 7.09 (s, 3H), 5.43 (s, 2H), 5.26 (s, 2H), 2.18 (s, 6H). ^13^C NMR (100 MHz, DMSO-*d*_6_) *δ* 167.72, 164.50, 158.12, 142.82, 135.56, 135.08, 134.66, 132.04, 129.25, 128.23, 127.24, 126.84, 125.17, 123.80, 115.34, 61.70, 52.14, 18.52 ppm. EI-MS (70 eV): m/z (%) = 481.8 (M^+^). Anal. Calcd for C_27_H_23_N_5_O_4_: C 67.35; H 4.81; N 14.54; Found: C 67.58; H 14.77; N 14.36.

### 2-(4-((4-(1,3-dioxoisoindolin-2-yl)phenoxy)methyl)-1H-1,2,3-triazol-1-yl)-N-(4-ethylphenyl)acetamide (11d)

Yield: 82% (394 mg). White crystal. M.p. 202–204 °C. IR (KBr): 3434, 1683, 1320, 1162 cm^−1^. ^1^H NMR (400 MHz, DMSO-*d*_6_) *δ* 10.47 (s, 1H), 8.34 (s, 1H), 7.92 (ddt, *J* = 21.1, 5.5, 3.2 Hz, 4H), 7.53 (d, *J* = 8.1 Hz, 2H), 7.39 (d, *J* = 8.6 Hz, 2H), 7.19 (dd, *J* = 20.9, 8.3 Hz, 4H), 5.39 (s, 2H), 5.27 (s, 2H), 2.55 (q, *J* = 7.6 Hz, 2H), 1.15 (t, *J* = 7.5 Hz, 3H). ^13^C NMR (100 MHz, DMSO-*d*_6_) *δ* 167.70, 164.39, 158.13, 142.83, 139.67, 136.57, 135.06, 132.01, 129.22, 128.55, 126.89, 125.17, 123.78, 119.78, 115.35, 61.72, 52.72, 28.07, 16.10 ppm. EI-MS (70 eV): m/z (%) = 481.3 (M^+^). Anal. Calcd for C_27_H_23_N_5_O_4_: C 67.35; H 4.81; N 14.54; Found: C 67.60; H 5.04; N 14.31.

### 2-(4-((4-(1,3-dioxoisoindolin-2-yl)phenoxy)methyl)-1H-1,2,3-triazol-1-yl)-N-(3-fluorophenyl)acetamide (11e)

Yield: 75%. White crystal. M.p. 204–206 °C. IR (KBr): 3441, 1686, 1328, 1201, 989 cm^−1^. ^1^H NMR (400 MHz, DMSO-*d*_6_) *δ* 10.75 (s, 1H), 8.33 (s, 1H), 7.93 (ddt, *J* = 20.0, 5.5, 3.2 Hz, 4H), 7.58 (dt, *J* = 11.6, 2.1 Hz, 1H), 7.46–7.25 (m, 4H), 7.21 (d, *J* = 8.5 Hz, 2H), 6.93 (td, *J* = 8.4, 2.5 Hz, 1H), 5.41 (s, 2H), 5.27 (s, 2H). ^13^C NMR (100 MHz, DMSO-*d*_6_)* δ* 167.72, 165.10, 163.79 (^1^*J*_C-F_ = 240 Hz), 158.11, 142.90, 140.61, (^3^*J*_C-F_ = 11 Hz), 135.07, 132.03, 131.13, 113.04, 129.25, 126.92, 125.18, 123.79, 115.48, 115.45 (^3^*J*_C-F_ = 10 Hz), 110.86 (^2^*J*_C-F_ = 21 Hz), 106.67 (^2^*J*_C-F_ = 26 Hz), 61.70, 52.71 ppm. EI-MS (70 eV): m/z (%) = 471.3 (M^+^).

Anal. Calcd for C_25_H_18_FN_5_O_4_: C 63.69; H 3.85; N 14.86; Found: C 63.48; H 4.69; N 14.99.

### N-(3-chlorophenyl)-2-(4-((4-(1,3-dioxoisoindolin-2-yl)phenoxy)methyl)-1H-1,2,3-triazol-1-yl)acetamide (11f.)

Yield: 71%. White crystal. M.p. 210–212 °C. IR (KBr): 3437, 1687, 1364, 1165, 758 cm^−1^. ^1^H NMR (400 MHz, DMSO-*d*_6_) *δ* 10.73 (s, 1H), 8.33 (s, 1H), 8.05–7.84 (m, 4H), 7.79 (s, 1H), 7.54–7.32 (m, 4H), 7.26–7.06 (m, 3H), 5.41 (s, 2H), 5.26 (s, 2H). ^13^C NMR (100 MHz, DMSO-*d*_6_) *δ* 167.72, 165.13, 158.11, 142.88, 140.28, 135.08, 133.67, 132.04, 131.13, 129.26, 126.93, 125.17, 124.00, 123.80, 119.21, 118.12, 115.35, 61.70, 52.71 ppm. EI-MS (70 eV): m/z (%) = 487.5 (M^+^). Anal. Calcd for C_25_H_18_ClN_5_O_4_: C 61.54; H 3.72; N 14.35; Found: C 61.27; H 3.88; N 14.55.

### N-(2,3-dichlorophenyl)-2-(4-((4-(1,3-dioxoisoindolin-2-yl)phenoxy)methyl)-1H-1,2,3-triazol-1-yl)acetamide (11 g)

Yield: 68% (354 mg). White crystal. M.p. 203– 205 °C. IR (KBr): 3444, 1691, 1330, 1171, 763 cm^−1^. ^1^H NMR (400 MHz, DMSO-*d*_6_) *δ* 10.29 (s, 1H), 8.33 (s, 1H), 7.93 (ddt, *J* = 20.2, 5.5, 3.1 Hz, 4H), 7.75 (dd, *J* = 8.2, 1.5 Hz, 1H), 7.50 (dd, *J* = 8.1, 1.4 Hz, 1H), 7.43–7.31 (m, 3H), 7.30–7.09 (m, 2H), 5.51 (s, 2H), 5.26 (s, 2H). ^13^C NMR (100 MHz, DMSO-*d*_6_) *δ* 167.72, 165.56, 158.11, 142.88, 136.62, 135.08, 132.46, 132.06, 129.25, 128.62, 127.55, 126.94, 125.43, 125.18, 125.18, 123.80, 115.34, 61.69, 52.44 ppm. EI-MS (70 eV): m/z (%) = 521.3 (M^+^).

Anal. Calcd for C_25_H_17_Cl_2_N_5_O_4_: C 57.49; H 3.28; N 13.41; Found: C 57.70; H 3.66; N 13.50.

### N-(2,4-dichlorophenyl)-2-(4-((4-(1,3-dioxoisoindolin-2-yl)phenoxy)methyl)-1H-1,2,3-triazol-1-yl)acetamide (11 h)

Yield: 74% (385 mg). White crystal. M.p. 232–234 °C. IR (KBr): 3435, 1688, 1341, 1210, 734 cm^−1^. ^1^H NMR (400 MHz, DMSO-*d*_6_) *δ* 10.20 (s, 1H), 8.31 (s, 1H), 8.05–7.84 (m, 4H), 7.80 (d, *J* = 8.8 Hz, 1H), 7.72 (d, *J* = 2.4 Hz, 1H), 7.45 (dd, *J* = 8.7, 2.4 Hz, 1H), 7.41–7.30 (m, 2H), 7.26–7.00 (m, 2H), 5.49 (s, 2H), 5.25 (s, 2H). ^13^C NMR (100 MHz, DMSO-*d*_6_) *δ* 167.72, 165.54, 158.11, 142.83, 135.09, 133.85, 132.07, 130.25, 129.55, 129.27, 128.19, 127.57, 127.31, 126.91, 125.17, 123.81, 115.33, 61.68, 52.42 ppm. EI-MS (70 eV): m/z (%) = 521.1 (M^+^). Anal. Calcd for C_25_H_17_Cl_2_N_5_O_4_: C 57.49; H 3.28; N 13.41; Found: C 57.28; H 3.44; N 13.19.

### N-(2,6-dichlorophenyl)-2-(4-((4-(1,3-dioxoisoindolin-2-yl)phenoxy)methyl)-1H-1,2,3-triazol-1-yl)acetamide (11i)

Yield: 66% (343 mg). White crystal. M.p. 202–204 °C. IR (KBr): 3446, 1693, 1389, 1251, 770 cm^−1^. ^1^H NMR (400 MHz, DMSO-*d*_6_) *δ* 10.49 (s, 1H), 8.32 (s, 1H), 7.93 (ddt, *J* = 20.2, 5.5, 3.1 Hz, 4H), 7.57 (d, *J* = 8.1 Hz, 2H), 7.39 (dd, *J* = 8.3, 5.0 Hz, 3H), 7.20 (d, *J* = 8.6 Hz, 2H), 5.47 (s, 2H), 5.25 (s, 2H). ^13^C NMR (100 MHz, DMSO-*d*_6_) *δ* 167.71, 164.95, 158.11, 142.89, 135.08, 133.91, 132.50, 132.06, 130.06, 129.25, 129.10, 126.93, 125.17, 123.80, 115.33, 61.67, 51.89 ppm. EI-MS (70 eV): m/z (%) = 521.6 (M^+^). Anal. Calcd for C_25_H_17_Cl_2_N_5_O_4_: C 57.49; H 3.28; N 13.41; Found: C 57.36; H 3.14; N 13.72.

### N-(4-bromophenyl)-2-(4-((4-(1,3-dioxoisoindolin-2-yl)phenoxy)methyl)-1H-1,2,3-triazol-1-yl)acetamide (11j)

Yield: 73%. White crystal. M.p. 237–239 °C. IR (KBr): 3443, 1685, 1344, 1158, 632 cm^−1^. ^1^H NMR (400 MHz, DMSO-*d*_6_) *δ* 10.66 (s, 1H), 8.33 (s, 1H), 7.93 (ddt, *J* = 20.0, 5.5, 3.1 Hz, 4H), 7.71–7.47 (m, 4H), 7.43–7.30 (m, 2H), 7.28–7.06 (m, 2H), 5.39 (s, 2H), 5.26 (s, 2H). ^13^C NMR (100 MHz, DMSO-*d*_6_) *δ* 167.71, 164.88, 158.11, 142.86, 138.25, 135.07, 132.22, 132.05, 129.25, 126.90, 125.17, 123.80, 121.62, 115.88, 115.34, 61.70, 52.73 ppm. EI-MS (70 eV): m/z (%) = 531.3 (M^+^). Anal. Calcd for C_25_H_18_BrN_5_O_4_: C 56.41; H 3.41; N 13.16; Found: C 56.66; H 3.20; N 13.35.

### 2-(4-((4-(1,3-dioxoisoindolin-2-yl)phenoxy)methyl)-1H-1,2,3-triazol-1-yl)-N-(4-nitrophenyl)acetamide (11 k)

Yield: 66% (328 mg). Light brown crystal. M.p. 260–262 °C. IR (KBr): 3430, 1691, 1552, 1349, 1178 cm^−1^. ^1^H NMR (400 MHz, DMSO-*d*_6_) *δ* 11.13 (s, 1H), 8.34 (s, 1H), 8.29–8.18 (m, 2H), 8.00–7.93 (m, 2H), 7.91 (td, *J* = 5.6, 5.1, 2.0 Hz, 2H), 7.87–7.77 (m, 2H), 7.45–7.31 (m, 2H), 7.27–6.94 (m, 2H), 5.48 (s, 2H), 5.27 (s, 2H). ^13^C NMR (100 MHz, DMSO-*d*_6_) *δ* 167.72, 165.82, 158.10, 144.97, 143.05, 142.88, 135.08, 132.06, 129.27, 126.90, 125.60, 125.18, 123.80, 119.50, 115.34, 61.68, 52.82 ppm. EI-MS (70 eV): m/z (%) = 498.2 (M^+^).

Anal. Calcd for C_25_H_18_N_6_O_6_: C 60.24; H 3.64; N 16.86; Found: C 60.39; H 3.47; N 17.02.

### 2-(4-((4-(1,3-dioxoisoindolin-2-yl)phenoxy)methyl)-1H-1,2,3-triazol-1-yl)-N-(2-methyl-4-nitrophenyl)acetamide (11 l)

Yield: 72%. Light brown crystal. M.p. 228–230 °C. IR (KBr): 3427, 1690, 1551, 1352, 1205 cm^−1^. ^1^H NMR (400 MHz, DMSO-*d*_6_) *δ* 10.09 (s, 1H), 8.34 (s, 1H), 8.17 (d, *J* = 2.6 Hz, 1H), 8.09 (dd, *J* = 8.9, 2.7 Hz, 1H), 8.00–7.81 (m, 5H), 7.43–7.32 (m, 2H), 7.25–7.11 (m, 2H), 5.55 (s, 2H), 5.27 (s, 2H), 2.43 (s, 3H). ^13^C NMR (100 MHz, DMSO-*d*_6_) *δ* 167.72, 165.73, 158.10, 143.94, 142.88, 142.58, 135.09, 132.05, 131.80, 129.27, 126.92, 126.02, 125.18, 123.80, 123.73, 122.32, 115.34, 61.69, 52.66, 18.32 ppm. EI-MS (70 eV): m/z (%) = 512.5 (M^+^). Anal. Calcd for C_26_H_20_N_6_O_6_: C 60.94; H 3.93; N 16.40; Found: C 61.16; H 4.14; N 16.59.

### N-benzyl-2-(4-((4-(1,3-dioxoisoindolin-2-yl)phenoxy)methyl)-1H-1,2,3-triazol-1-yl)acetamide (11m)

Yield: 80%. White crystal. M.p. 201–203 °C. IR (KBr): 3445, 1689, 1388, 1220 cm^−1^. ^1^H NMR (400 MHz, DMSO-*d*_6_) *δ* 8.89 (t, *J* = 5.8 Hz, 1H), 8.28 (s, 1H), 7.93 (ddd, *J* = 24.4, 5.6, 3.1 Hz, 4H), 7.49–6.82 (m, 9H), 5.24 (s, 2H), 5.23 (s, 2H), 4.35 (d, *J* = 5.7 Hz, 2H). ^13^C NMR (100 MHz, DMSO-*d*_6_) *δ* 167.72, 165.90, 158.13, 142.78, 139.17, 135.08, 132.04, 129.25, 128.84, 127.87, 127.48, 126.80, 125.16, 123.80, 115.34, 61.71, 52.12, 42.87 ppm. EI-MS (70 eV): m/z (%) = 467.3 (M^+^). Anal. Calcd for C_26_H_21_N_5_O_4_: C 66.80; H 4.53; N 14.98; Found: C 66.68; H 4.74; N 15.15.

### 2-(4-((4-(1,3-dioxoisoindolin-2-yl)phenoxy)methyl)-1H-1,2,3-triazol-1-yl)-N-(4-fluorobenzyl)acetamide (11n)

Yield: 78% (367 mg). White crystal. M.p. 204–206 °C. IR (KBr): 3439, 1685, 1313, 1224, 963 cm^−1^. ^1^H NMR (400 MHz, DMSO-*d*_6_) *δ* 8.89 (t, *J* = 5.8 Hz, 1H), 8.28 (s, 1H), 7.93 (ddt, *J* = 19.7, 5.3, 3.1 Hz, 5H), 7.45–7.28 (m, 4H), 7.26–7.08 (m, 4H), 5.24 (s, 2H), 5.22 (S, 2H), 4.33 (d, *J* = 4.9 Hz, 2H). ^13^C NMR (100 MHz, DMSO-*d*_6_) *δ* 167.72, 165.93, 162.94 (^1^*J*_C-F_ = 241 Hz), 158.12, 142.82, 135.44 (^4^*J*_C-F_ = 3 Hz), 135.08, 132.05, 129.92 (^3^*J*_C-F_ = 8 Hz), 129.25, 126.83, 126.82, 125.16, 123.80, 115.67 (^2^*J*_C-F_ = 22 Hz), 115.33, 61.71, 52.10, 42.14 ppm. EI-MS (70 eV): m/z (%) = 485.3 (M^+^). Anal. Calcd for C_25_H_18_FN_5_O_4_: C 63.69; H 3.85; N 14.86; Found: C 63.82; H 4.08; N 15.01.

### In vitro α-glucosidase inhibition assay

*Saccharomyces cerevisiae* (*S. cerevisiae*) form of *α*-glucosidase (EC3.2.1.20, 20 U/mg) and *p*-nitrophenyl glucopyranoside (substrate) were purchased from Sigma-Aldrich. *α*-Glucosidase solution was produced in potassium phosphate buffer (PPB, pH 6.8, 50 mM) and phthalimide-phenoxy-1,2,3-triazole-*N*-phenyl (or benzyl) acetamides **11a**–**n** were dissolved in DMSO (10% final concentration)^18^. The various concentrations of the latter compounds **11a**–**n** (20 µL), α-glucosidase solution (20 µL) and PPB (135 µL) were added in the 96-well plate and incubated at 37 °C for 10 min. Then, substrate (25 µL, 4 mM) was added to this plate and allowed to incubate at 37 °C for 20 min. The change in absorbance was measured at 405 nm using by a standard spectrophotometer (Gen5, Power wave xs2, BioTek, America). Acarbose and DMSO (10% final concentration) were used as positive and negative controls, respectively.

### Kinetic study

The kinetic study was performed on the most potent compound **11j** to determine inhibition mode of the newly synthesized compounds. The 20 µL of α-glucosidase solution (1 U/mL) was incubated with different concentrations of compound **11j** (0, 12, 23 and 46 µM) for 15 min at 30 °C. The reaction was then started by adding different concentrations of substrate (1–10 mM) and change in absorbance was measured for 20 min at 405 nm using by spectrophotometer (Gen5, Power wave xs2, BioTek, America).

### Docking study

Homology model of α-glucosidase was prepared based on the described method by Imran et al.^19,20^. In the first step, a PDB with high sequence similarity with *S. cerevisiae* α-glucosidase was searched by SWISS-MODEL and *S. cerevisiae* isomaltase with PDB code: 3A4A) was selected. This enzyme was 72% identical and had 85% similarity with the *S. cerevisiae* α-glucosidase. In the second step, *S. cerevisiae* isomaltase was subjected through sequence alignment and homology model was constructed using by automated homology modeling pipeline SWISS-MODEL (managed by Swiss Institute of Bioinformatics) and the quality of the obtained homology model was verified using PROCHECK^21^.

In the third step docking study of acarbose and the selected compounds was performed in the active site of modeled α-glucosidase. The 3D structures of the acarbose and selected inhibitors were built by MarvineSketch 5.8.3, 2012, ChemAxon (http://www.chemaxon.com) and converted to pdbqt coordinate using Auto dock Tools. The pdbqt coordinate of enzyme was produced using the same software. Prepared pdbqt files were used as input files for the AUTOGRID program. In this program for each atom type in the selected ligand, maps were calculated with 0.375 Å spacing between grid points and the center of the grid box was placed at x = 12.5825, y = 7.8955, and z = 12.519. The dimensions for the active site box were set at 40 × 40 × 40 Å. Flexible ligand dockings were accomplished for the selected ligands. Each docked system was carried out by 50 runs of the AUTODOCK program search by the Lamarckian genetic algorithm (LGA). The best poses of the selected ligands were selected for analyzing the interactions between target enzyme and the selected ligand. The obtained results were visualized using BIOVIA Discovery Studio v.3.5.

### Molecular dynamics

MD simulations were performed using Groningen machine for chemical simulations (GROMACS) 5.1.2^22^. Topology files and other force field parameters of the selected compounds were made by SwissParam server^23^. Protein topology file was constructed by using the pdb2gmx command and CHARMM27 all-atom force field (CHARM22 plus CMAP for proteins). The protein–ligand complex (in.gro format) was created in Notepad +  + and the topology file of the protein was edited to include topology parameters of ligand as well. The resulting complex was centered in a cubic box with a side length of 2.0 nm and the SPC216 water model was used to fill the system. The net negative charge of the protein was neutralized by fifteen Na + ions which replaced the same number of water molecules. The Steepest descent minimization algorithm was used for the minimization of the system in a maximum number of 50,000 steps until the maximum force became less than 10.0 kJ/mol. For NVT equilibration the v-rescale algorithm was used in 300 K with a coupling constant of 0.1 ps and time duration of 500 ps. The last phase in preparation of the system was NPT equilibration. In this step, Berenson pressure coupling algorithm with a coupling constant of 5.0 ps was applied for 1000 ps of NPT simulation. Particle Mesh Ewald (PME) algorithm was used for long-range electrostatics and cut-off method for van der Waals interactions. Cut off distances were set at 1.0 nm for the calculation of the electrostatic and 1.2 nm for van der Waals interactions. Finally, 20 ns MD simulation was performed for the protein–ligand complex.

### In silico pharmacokinetic and toxicity predictions

In silico prediction of pharmacokinetic properties and toxicity profile of the positive control acarbose and the most potent compounds **11j** and **11i** was performed using by the preADMET online server^24^.

### Ethical approval and consent to participate

The ethics code for this work is IR.NIMAD. REC.1400.155 (https://ethics.research.ac.ir/IR.NIMAD.REC.1400.155).

## Supplementary Information

Supplementary Information.

## Data Availability

The datasets used or analyzed during the current study are available from the corresponding author on reasonable request.
