# Supplementary material for: Design, synthesis, in vitro anti-α-glucosidase evaluations, and computational studies of new phthalimide-phenoxy-1,2,3-triazole-N-phenyl (or benzyl) acetamides as potential anti-diabetic agents
Source: Sci Rep. 2023 Jun 20;13:10030. doi: 10.1038/s41598-023-36890-y (PMC10282079; doi:10.1038/s41598-023-36890-y)
Supplement: Supplementary file 1 — Supplementary Information. [file 41598_2023_36890_MOESM1_ESM.docx]

**Support information**

**2-(4-((4-(1,3-dioxoisoindolin-2-yl)phenoxy)methyl)-1*H*-1,2,3-triazol-1-yl)-*N*-phenylacetamide (11a)**


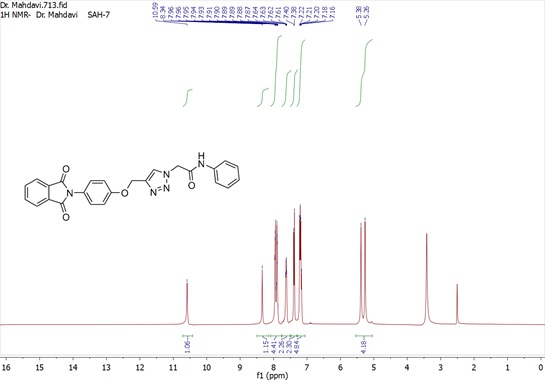


**
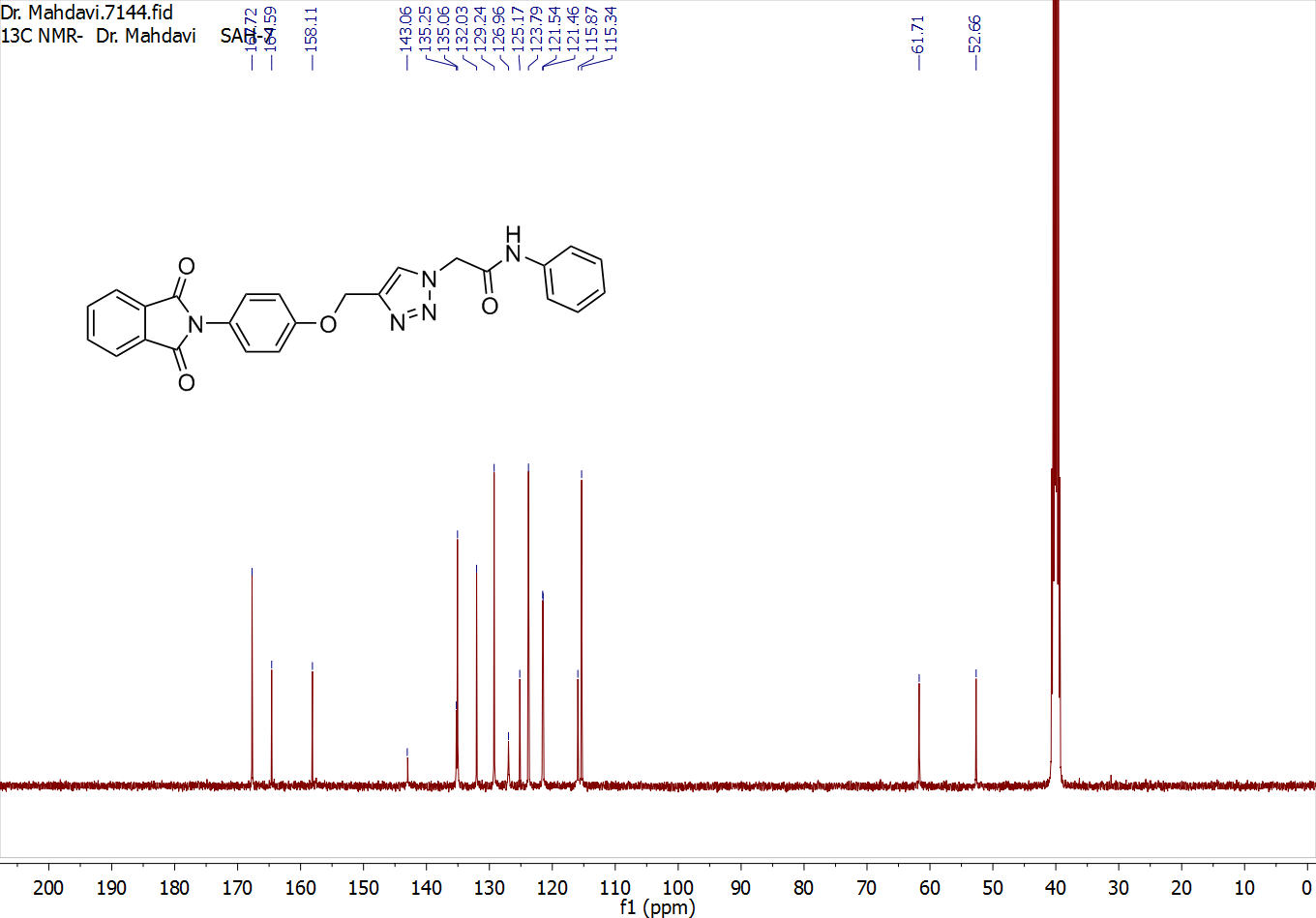
**

**
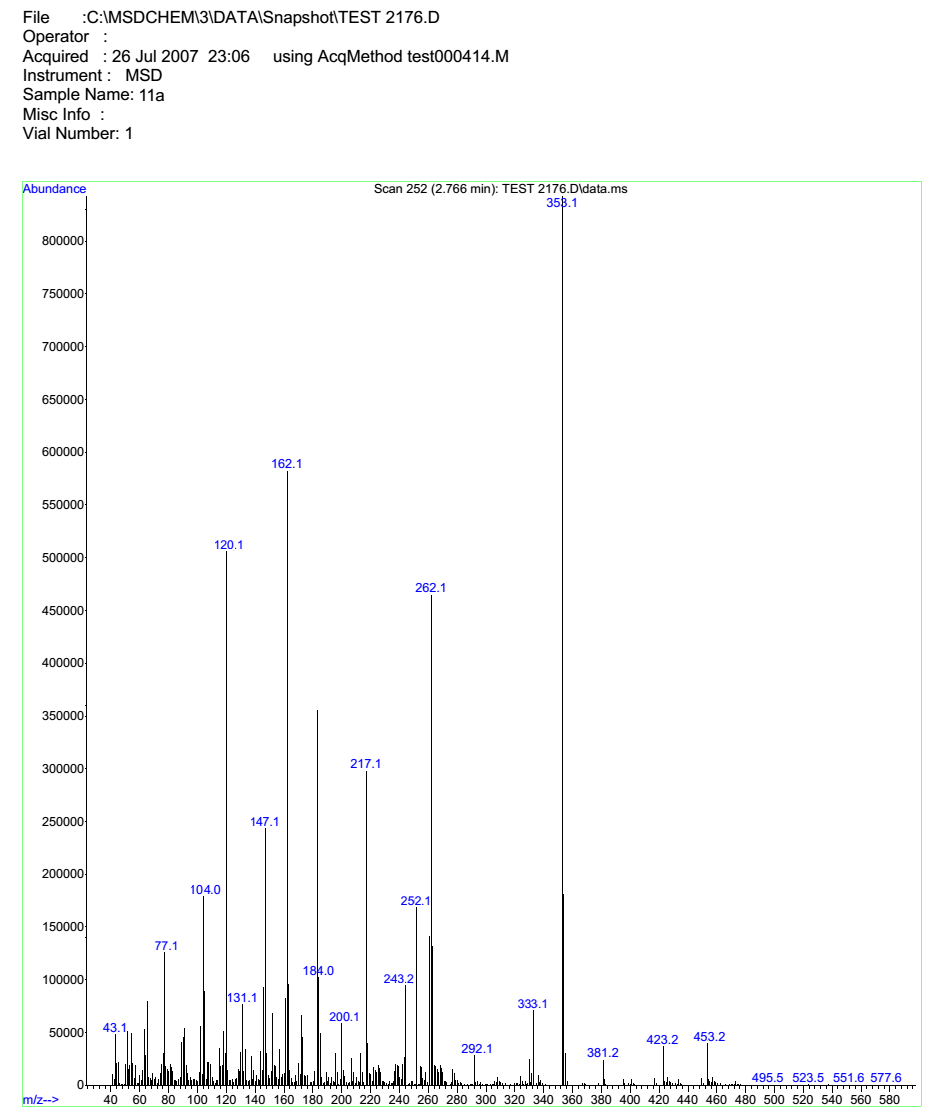
**

***N*-(2,3-dimethylphenyl)-2-(4-((4-(1,3-dioxoisoindolin-2-yl)phenoxy)methyl)-1*H*-1,2,3-triazol-1-yl)acetamide (11b)**


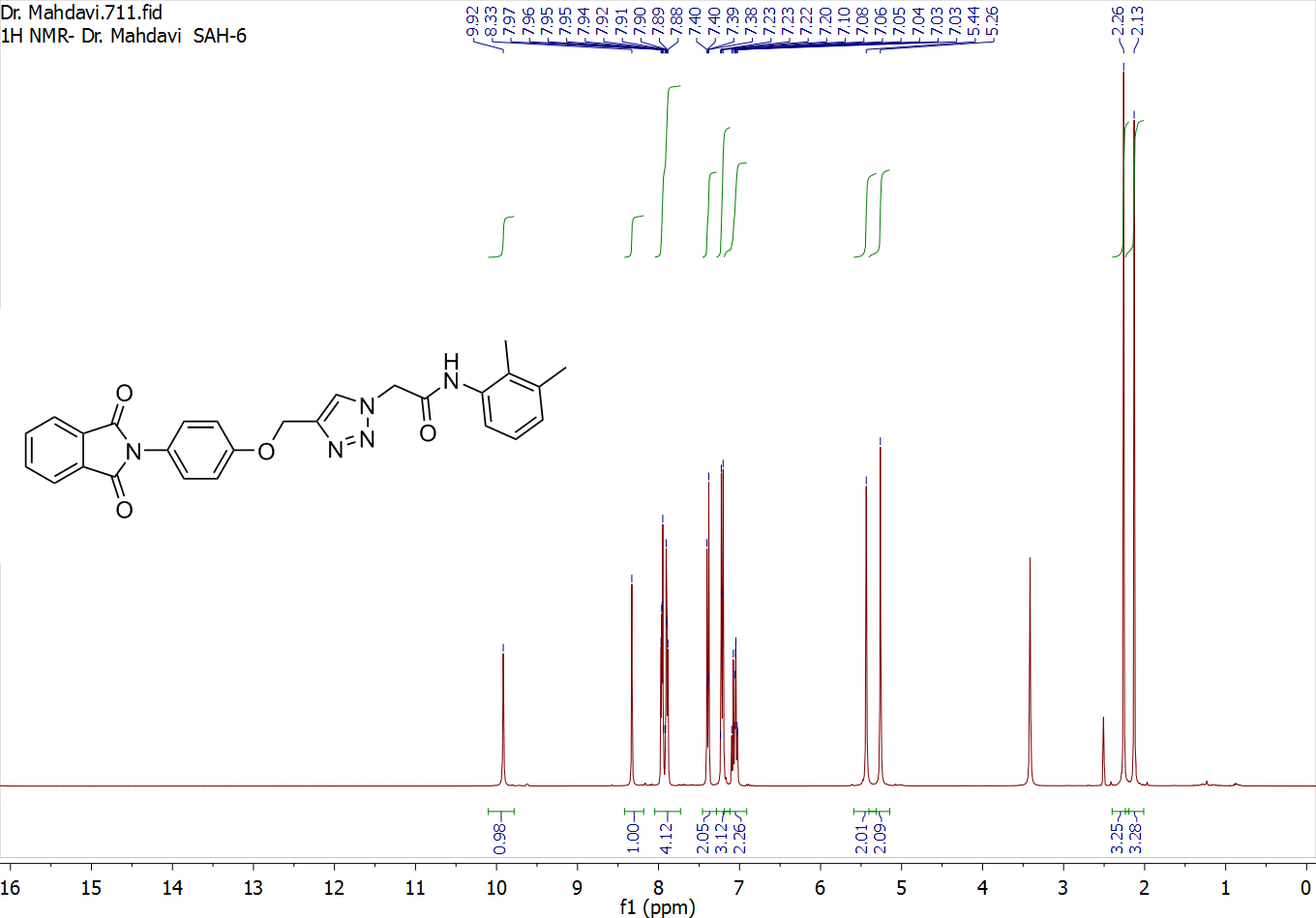


**
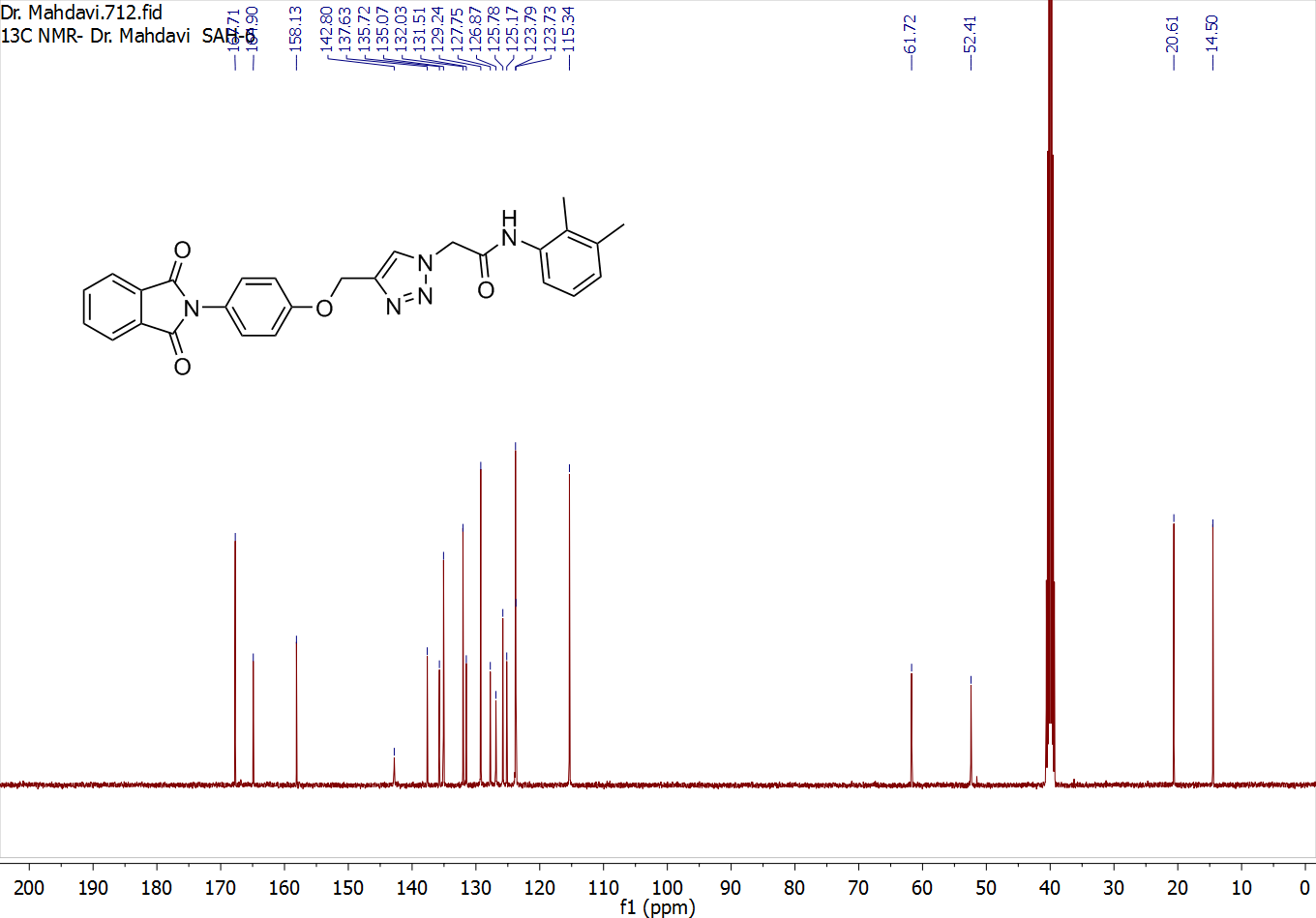
**

**
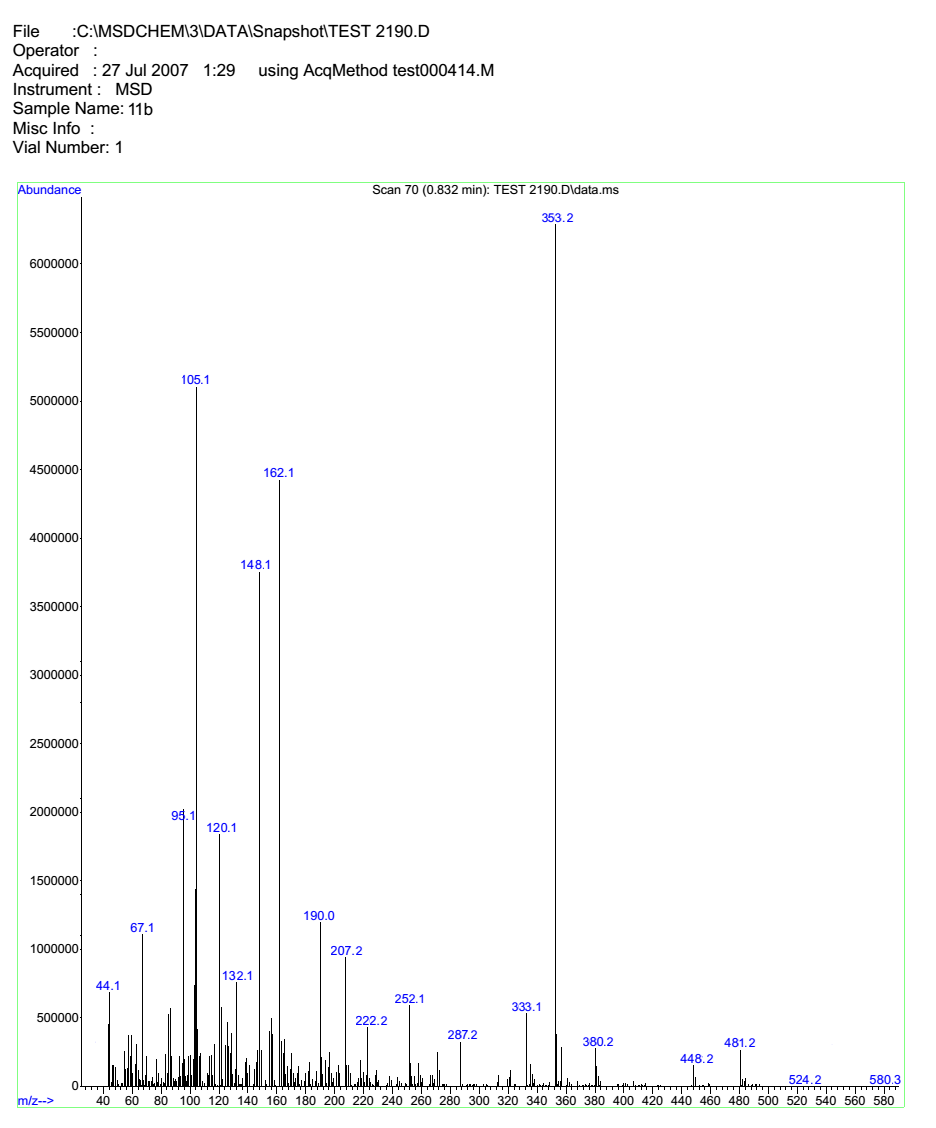
**

***N*-(2,6-dimethylphenyl)-2-(4-((4-(1,3-dioxoisoindolin-2-yl)phenoxy)methyl)-1*H*-1,2,3-triazol-1-yl)acetamide (11c)**


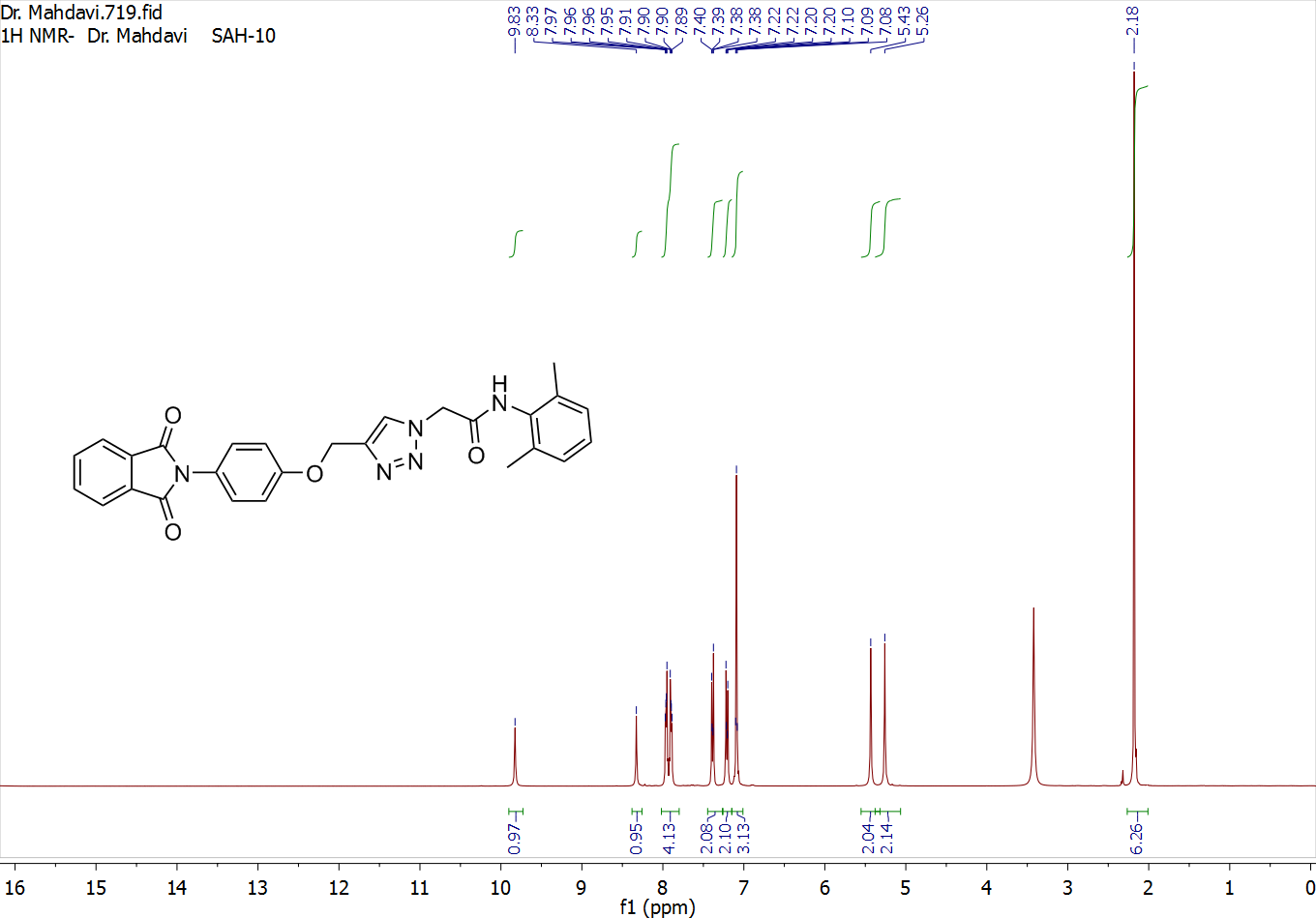


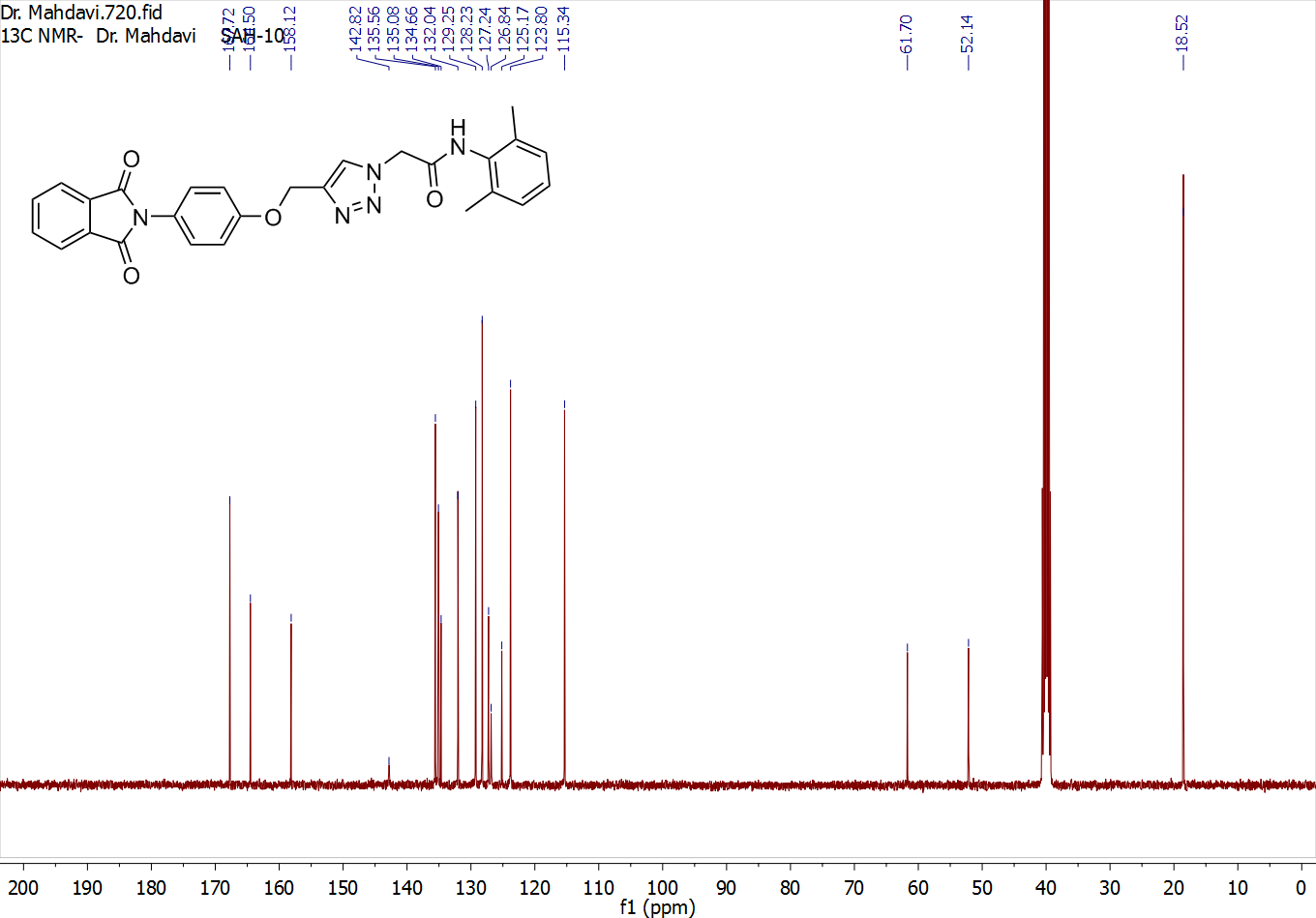


**
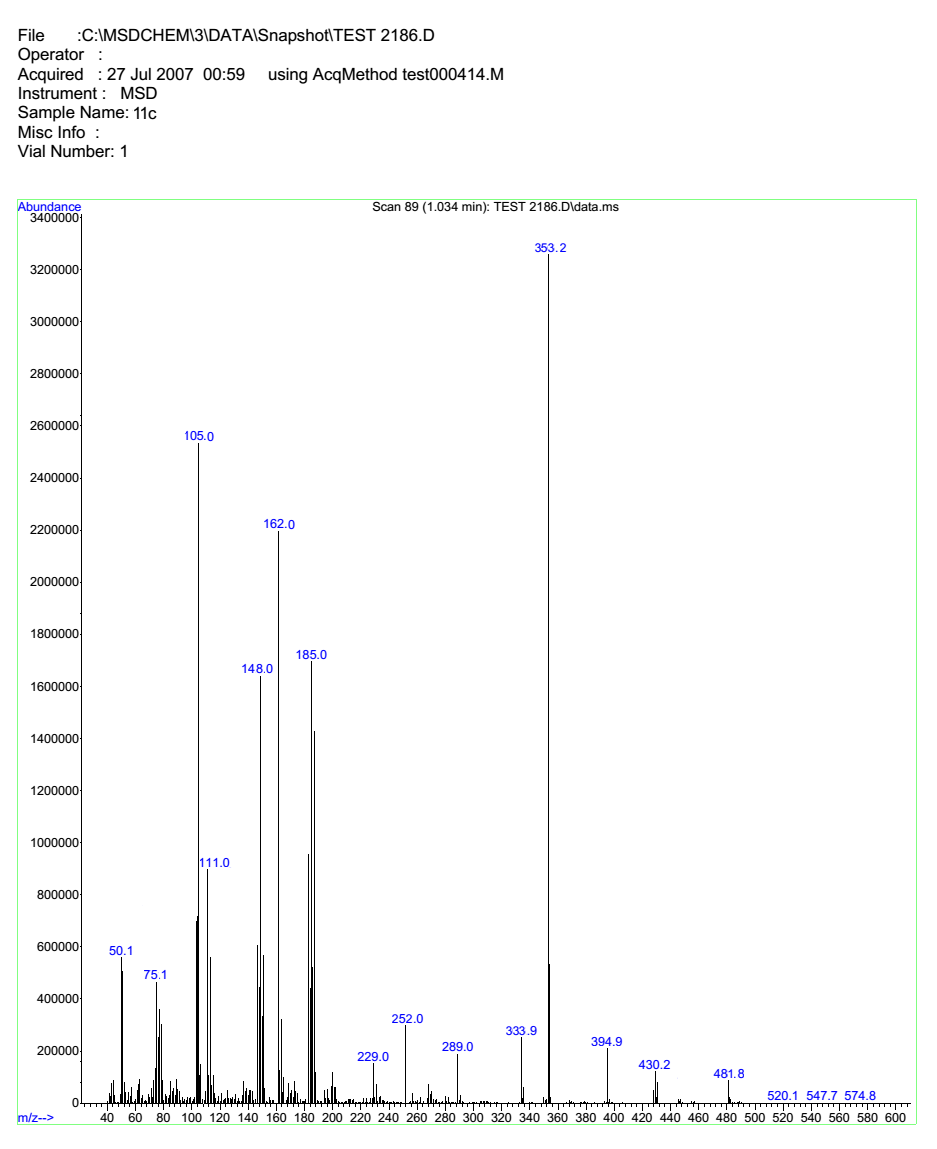
**

**2-(4-((4-(1,3-dioxoisoindolin-2-yl)phenoxy)methyl)-1*H*-1,2,3-triazol-1-yl)-*N*-(4-ethylphenyl)acetamide (11d)**


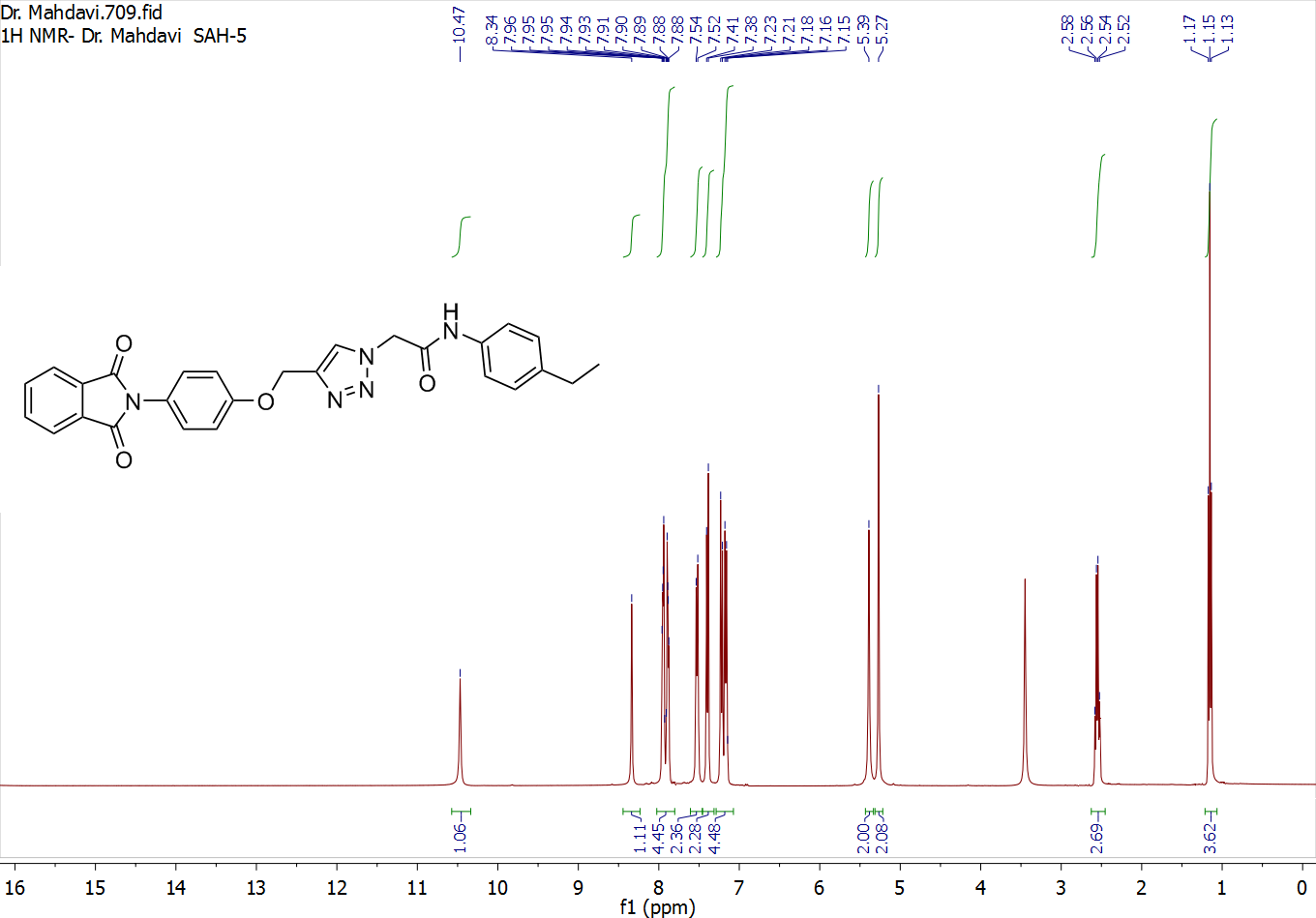


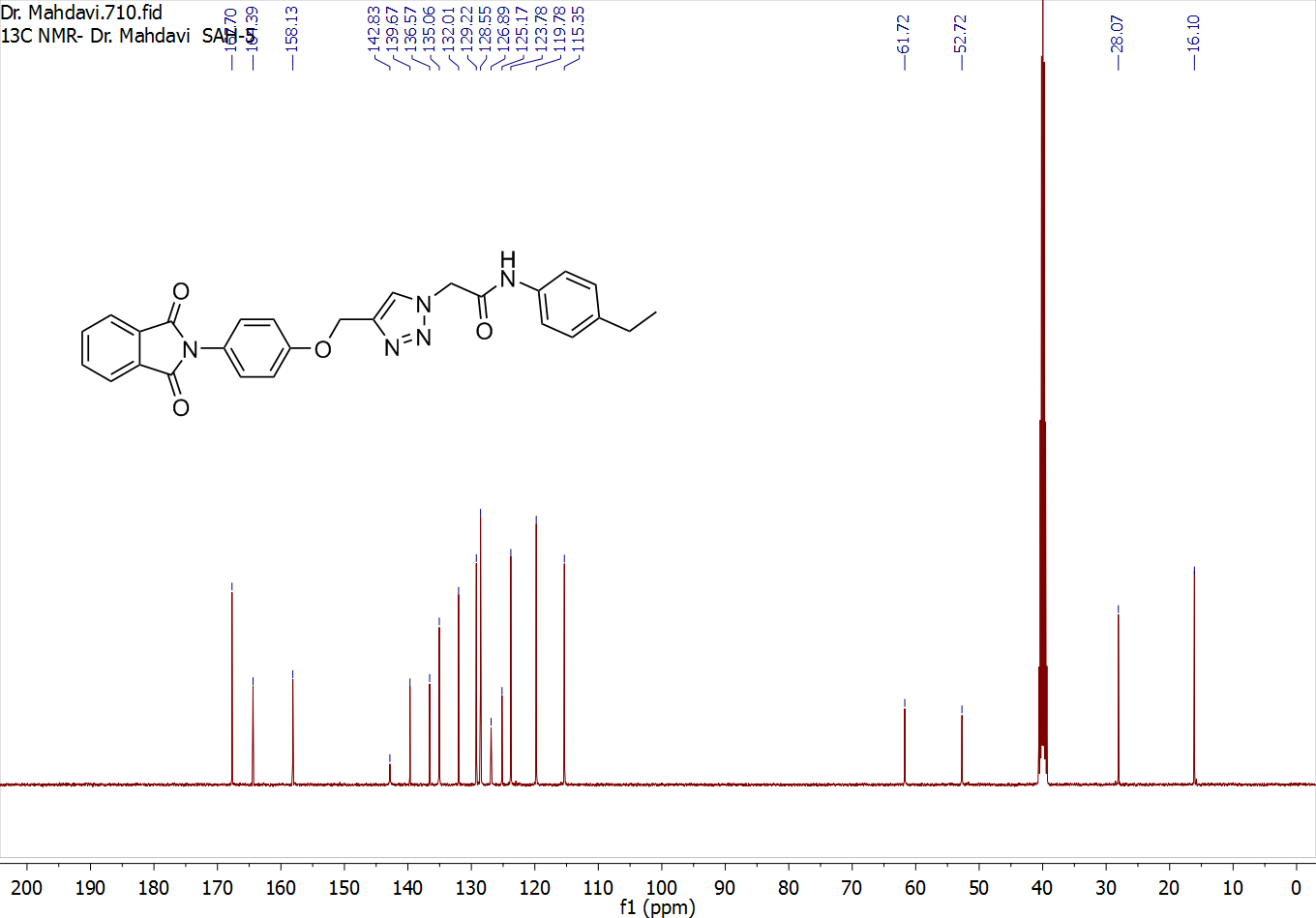


**
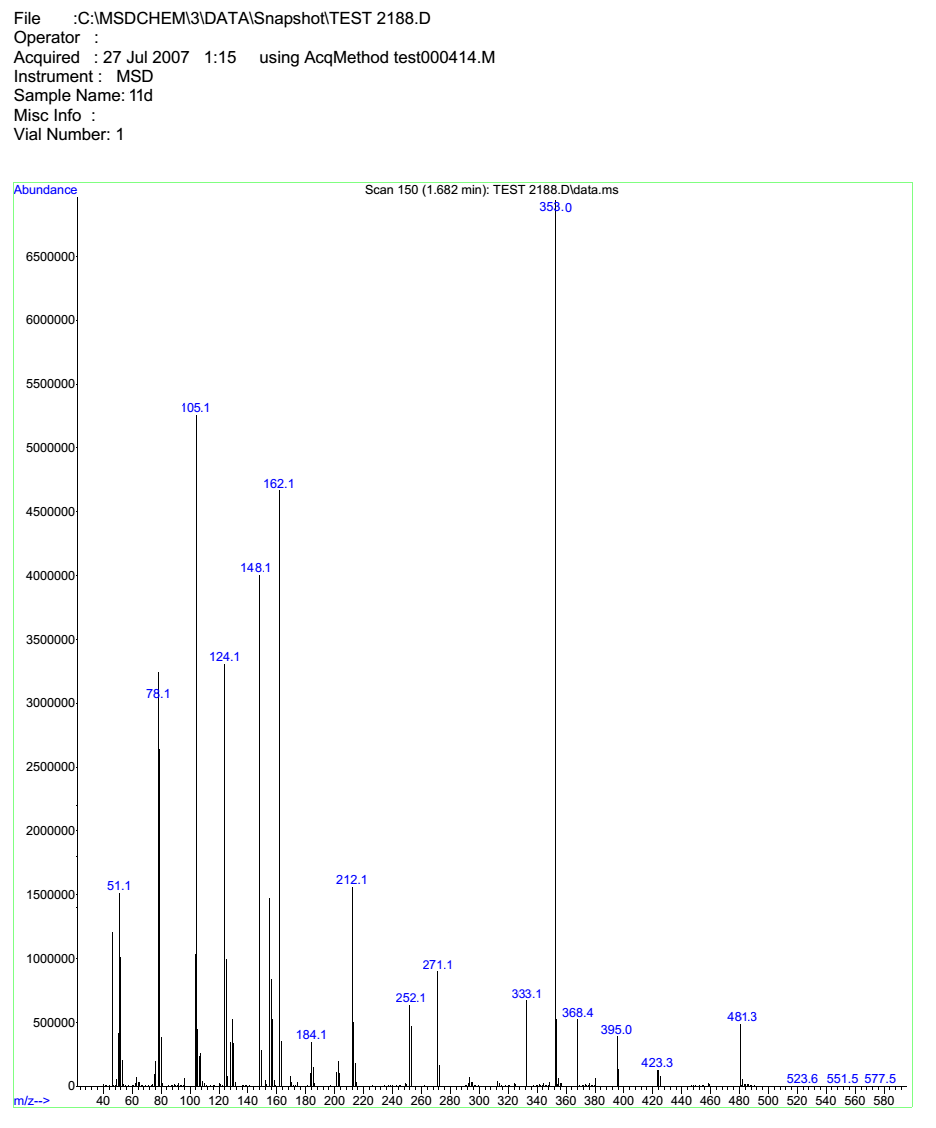
**

**2-(4-((4-(1,3-dioxoisoindolin-2-yl)phenoxy)methyl)-1*H*-1,2,3-triazol-1-yl)-*N*-(3-fluorophenyl)acetamide (11e)**


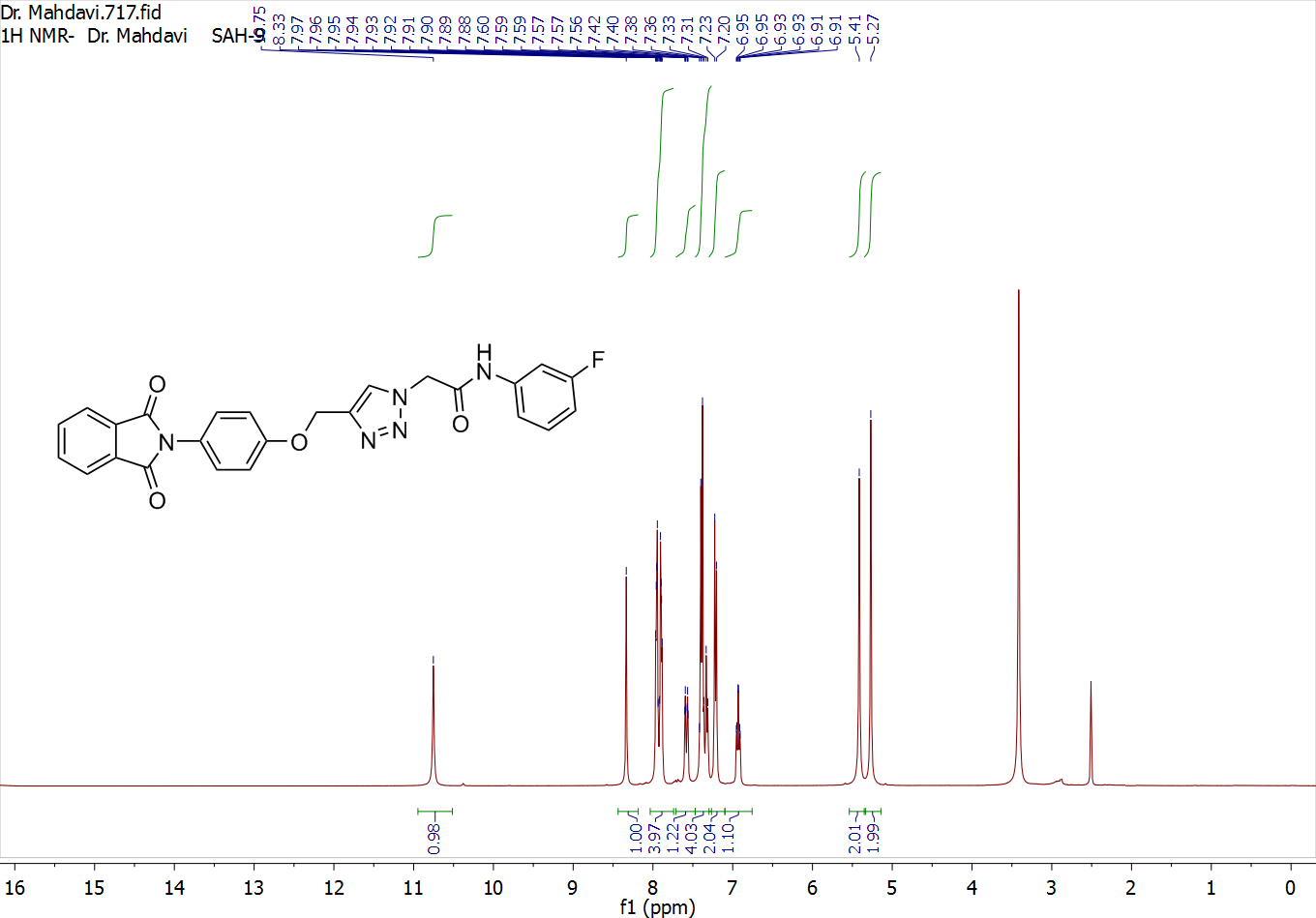


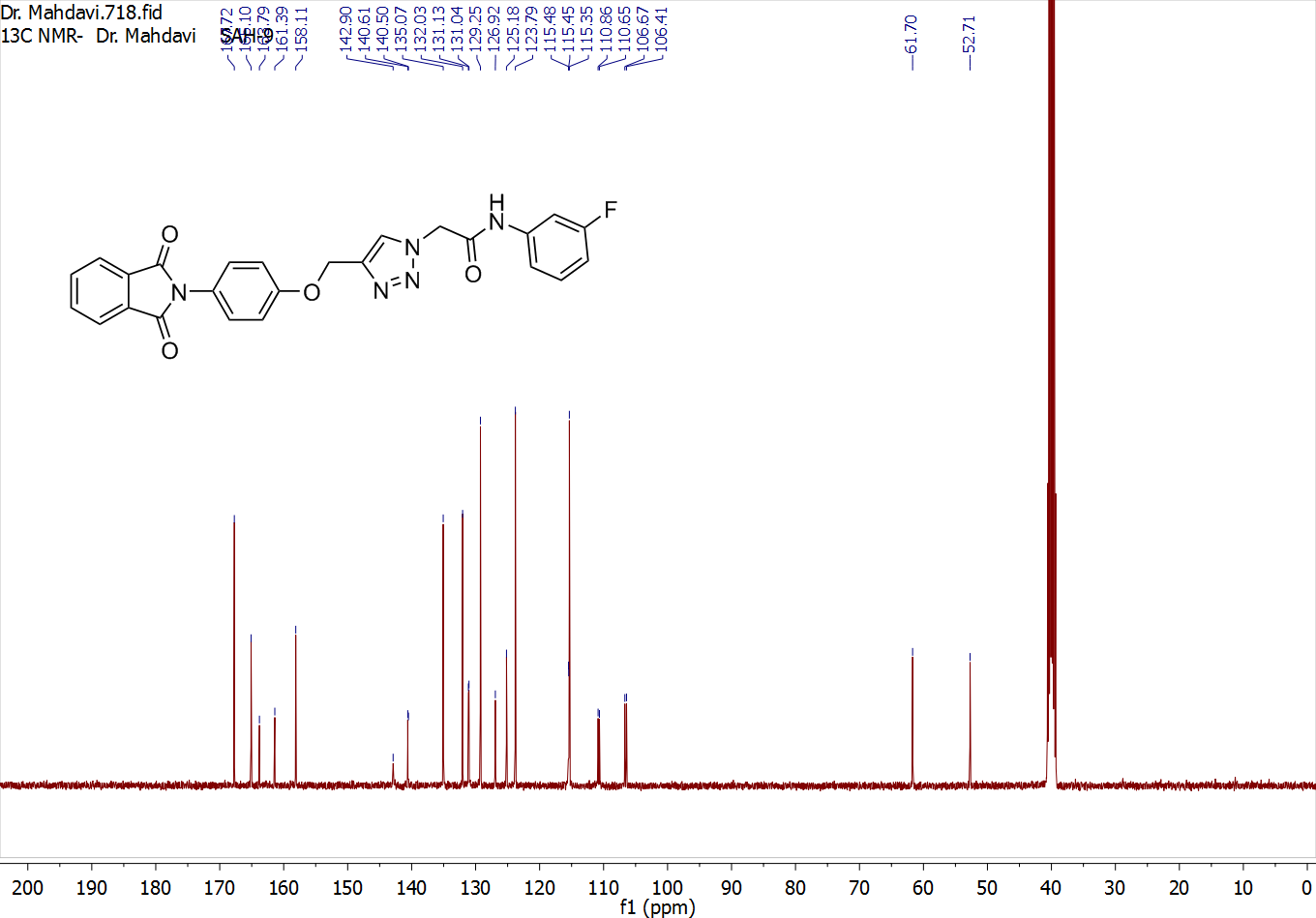


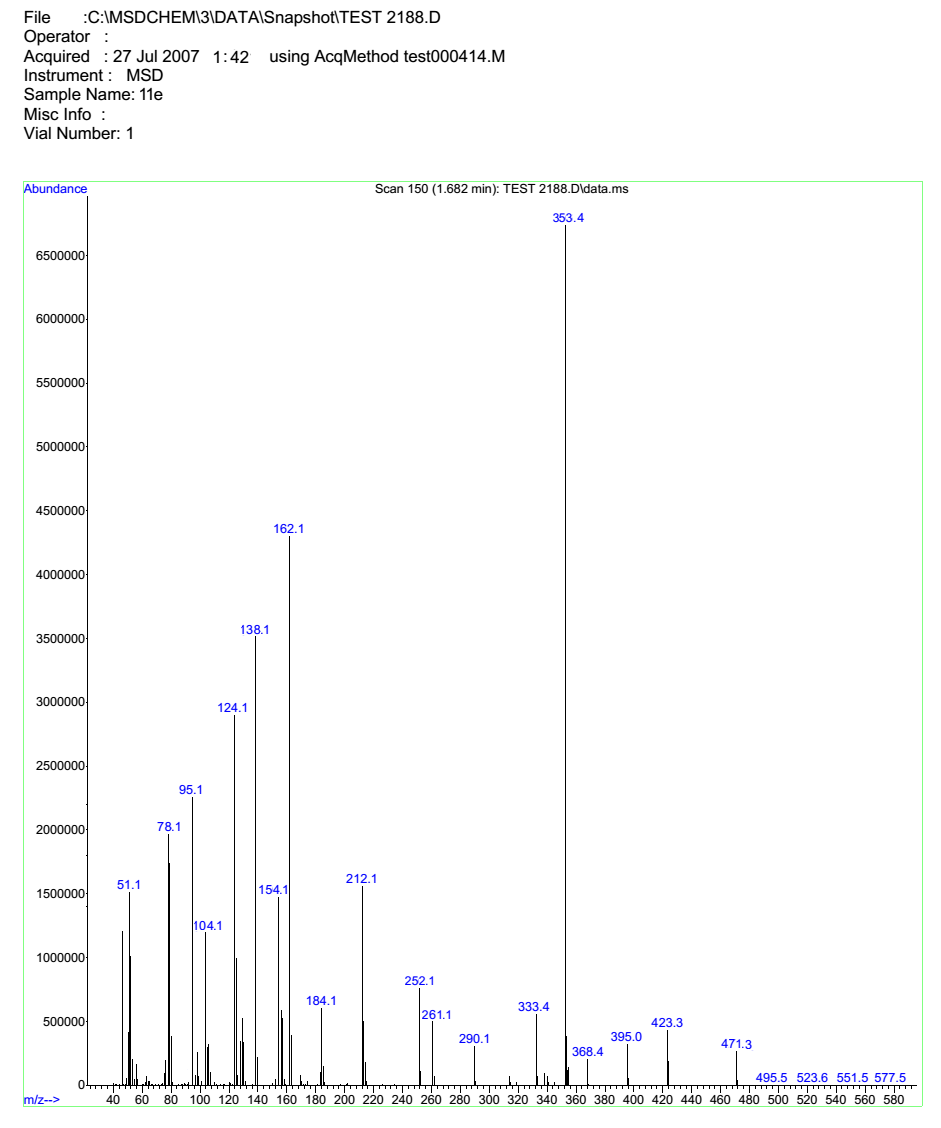


***N*-(3-chlorophenyl)-2-(4-((4-(1,3-dioxoisoindolin-2-yl)phenoxy)methyl)-1*H*-1,2,3-triazol-1-yl)acetamide (11f)**


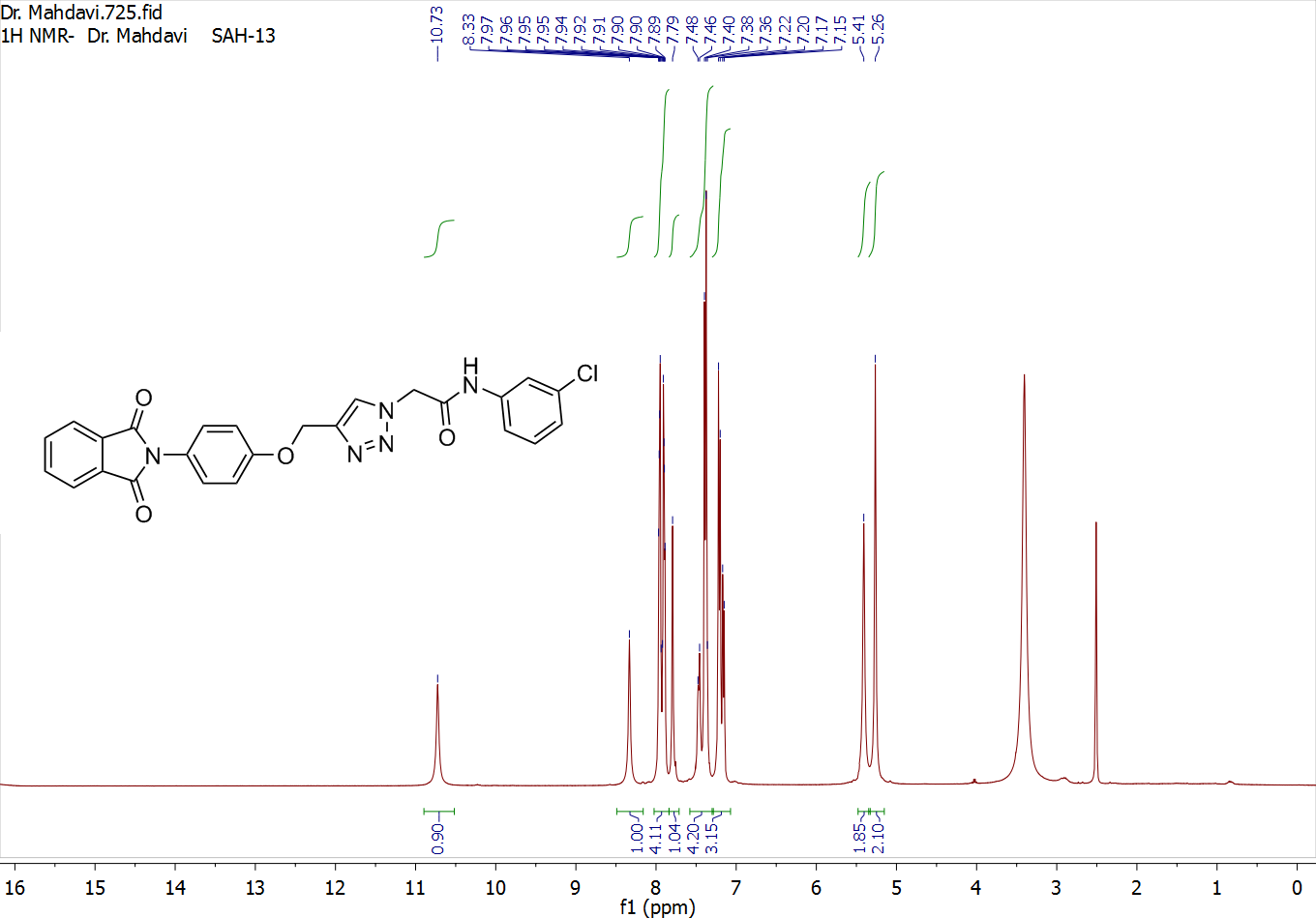


**
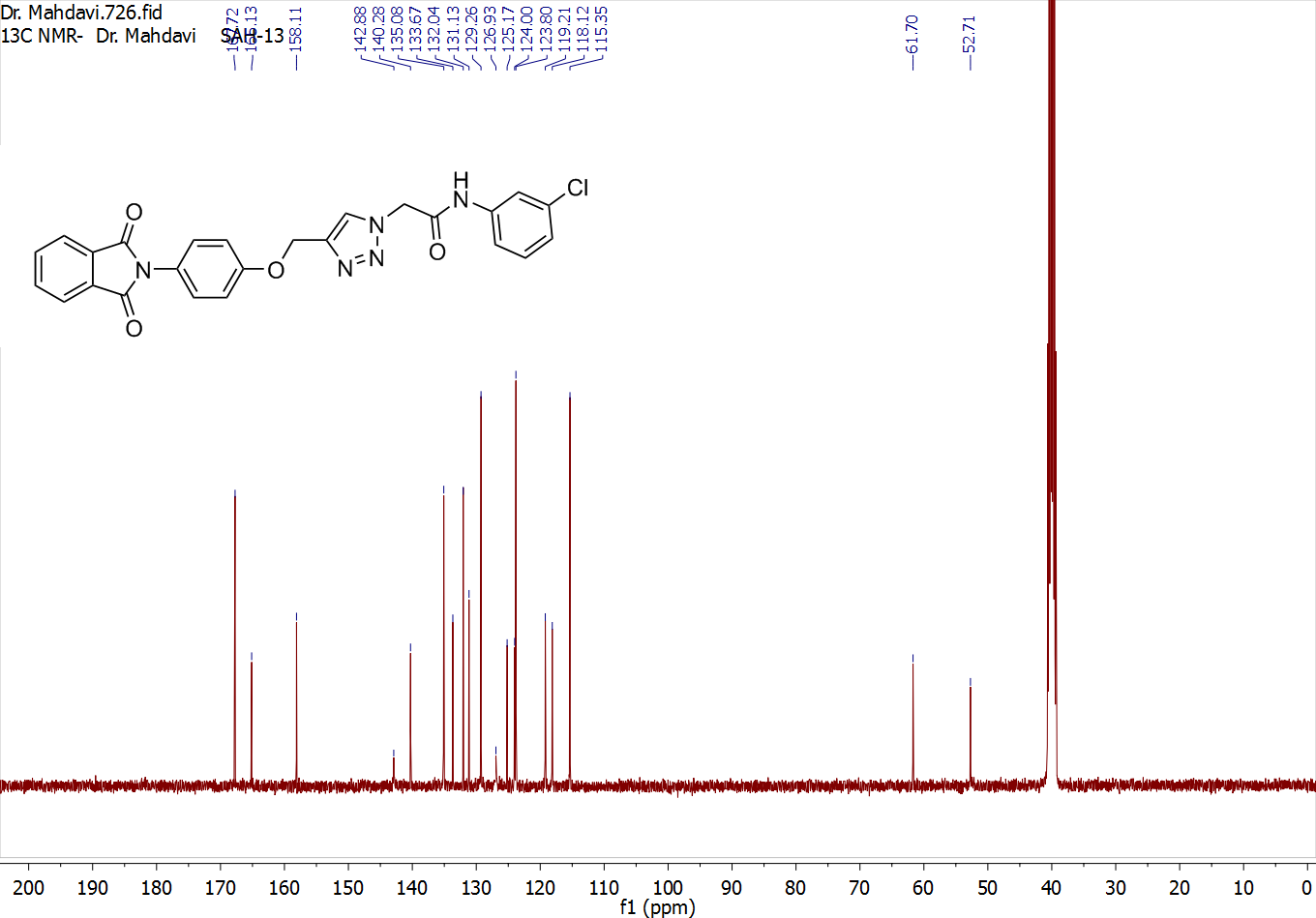
**


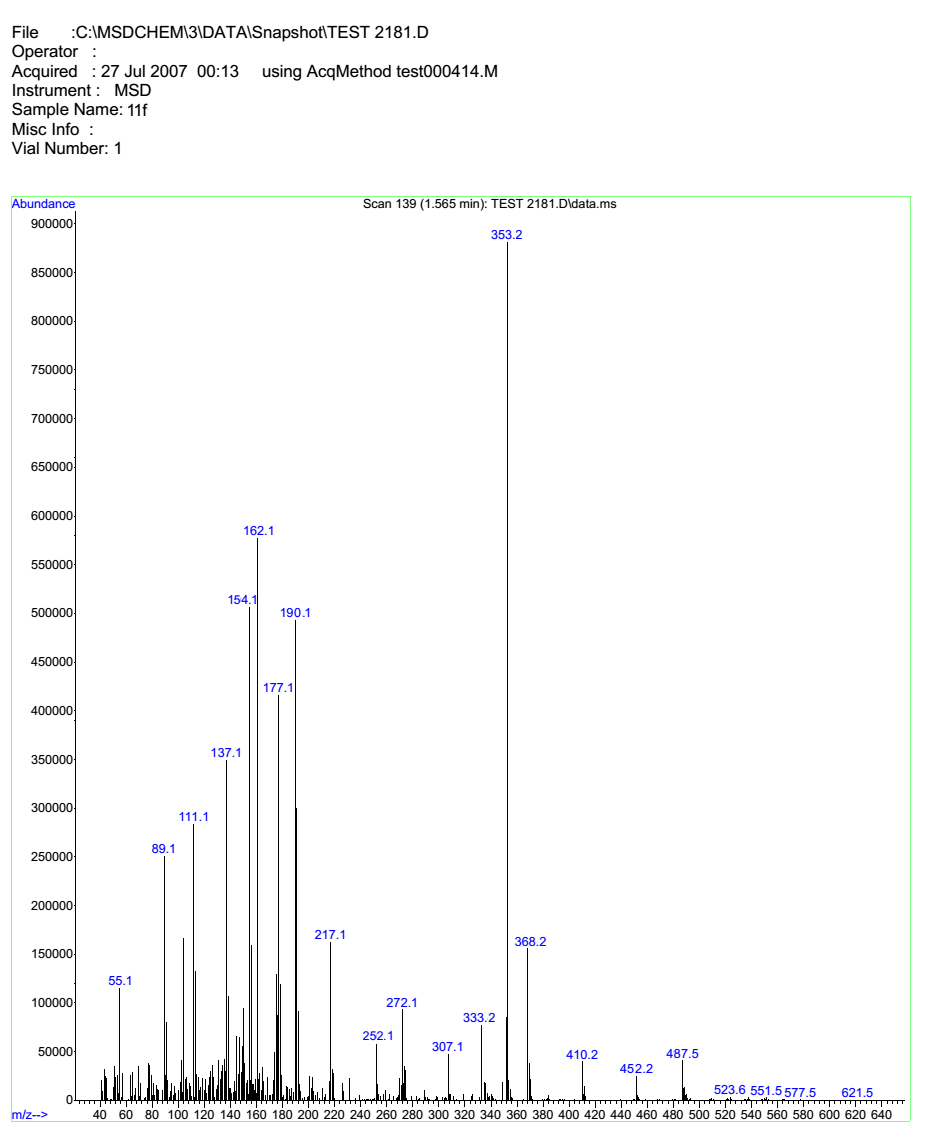


***N*-(2,3-dichlorophenyl)-2-(4-((4-(1,3-dioxoisoindolin-2-yl)phenoxy)methyl)-1*H*-1,2,3-triazol-1-yl)acetamide (11g)**


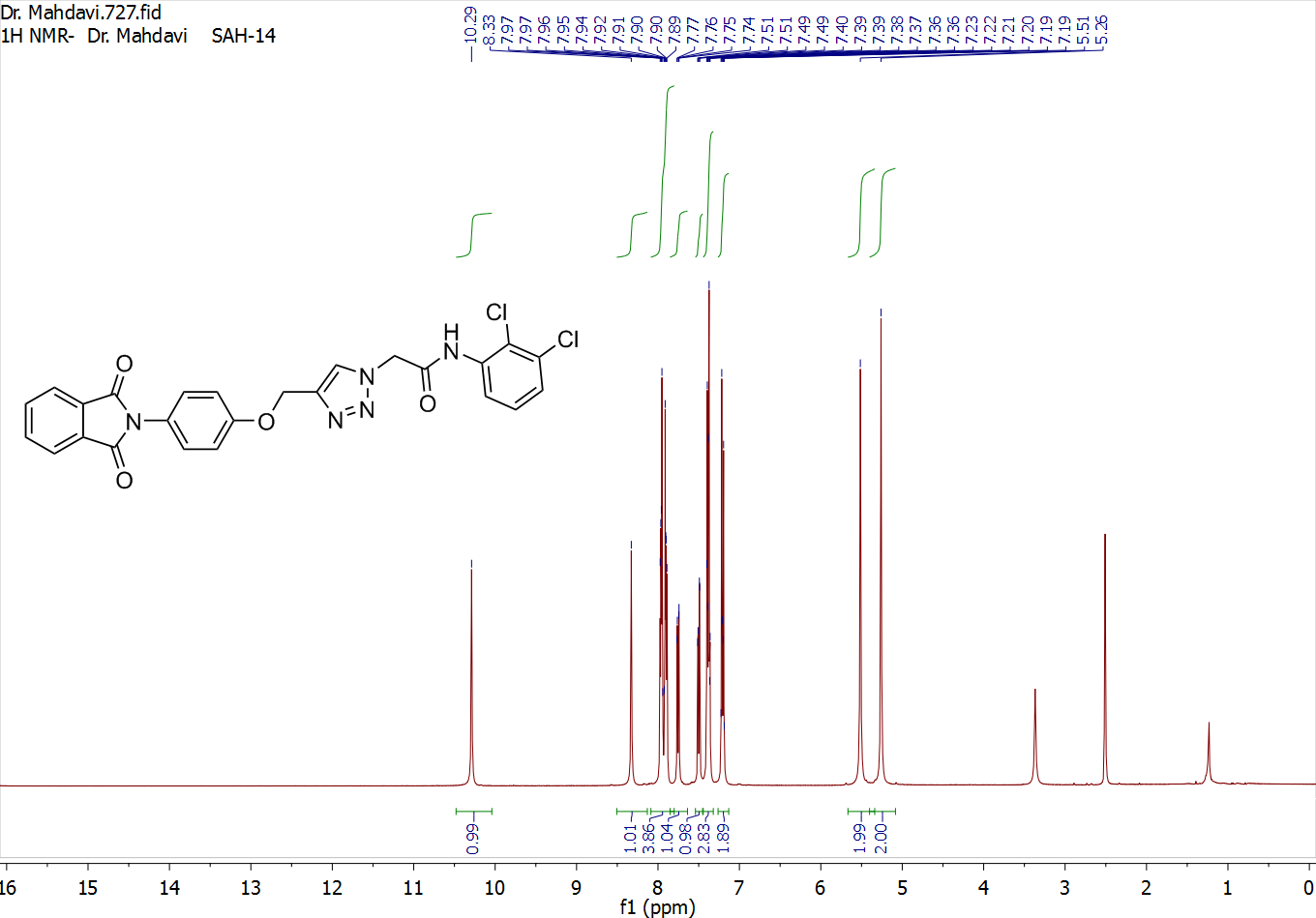


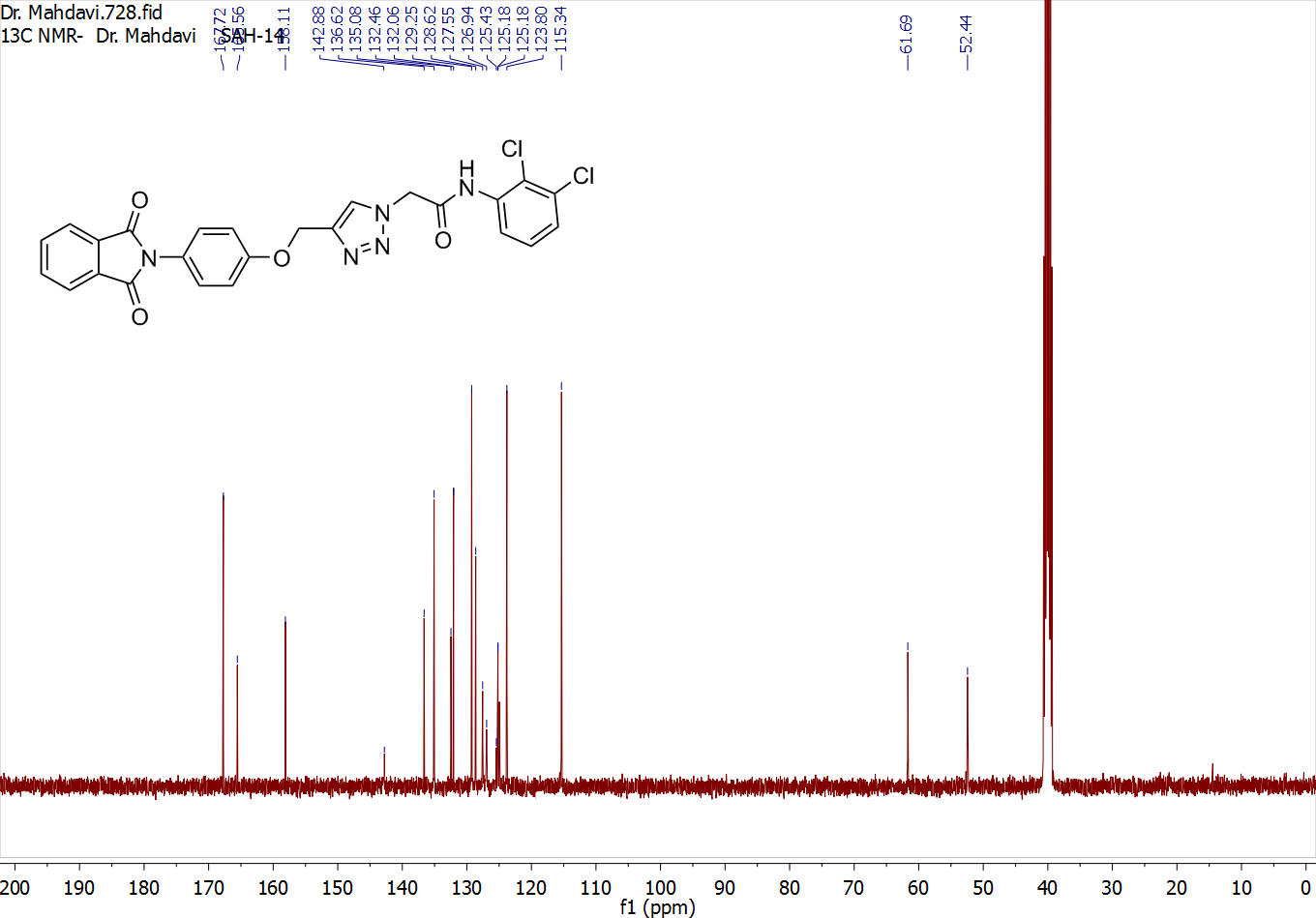


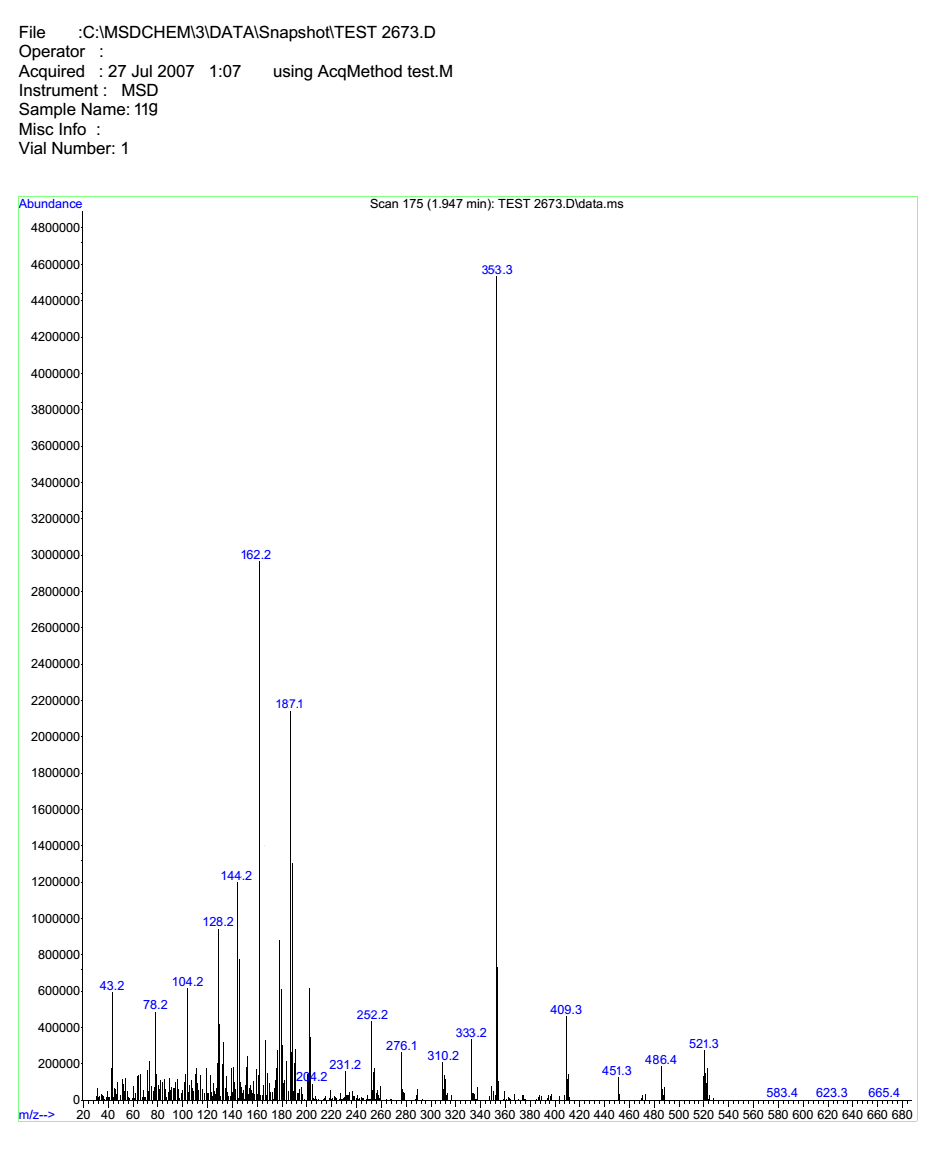


***N*-(2,4-dichlorophenyl)-2-(4-((4-(1,3-dioxoisoindolin-2-yl)phenoxy)methyl)-1*H*-1,2,3-triazol-1-yl)acetamide (11h)**


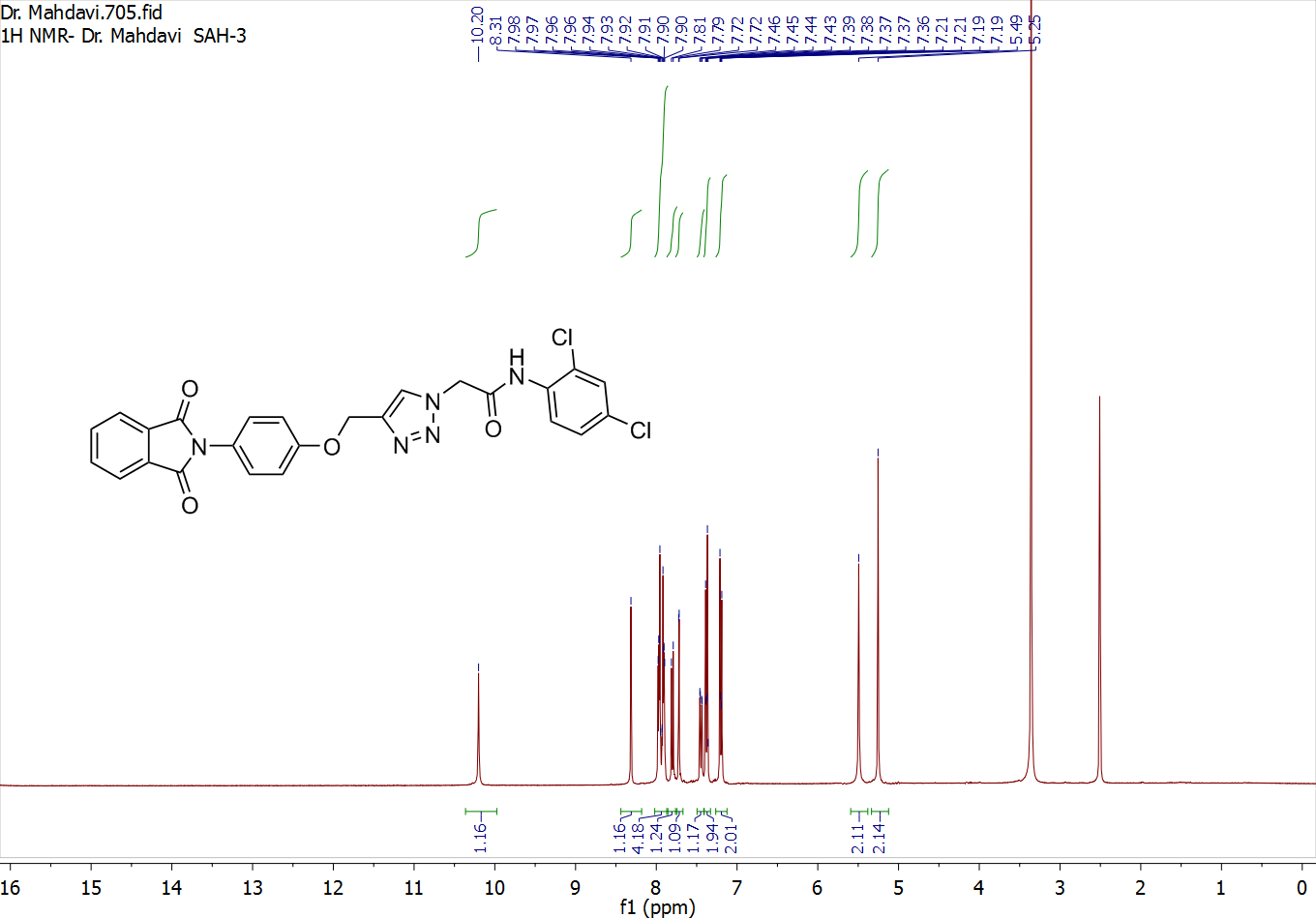


**
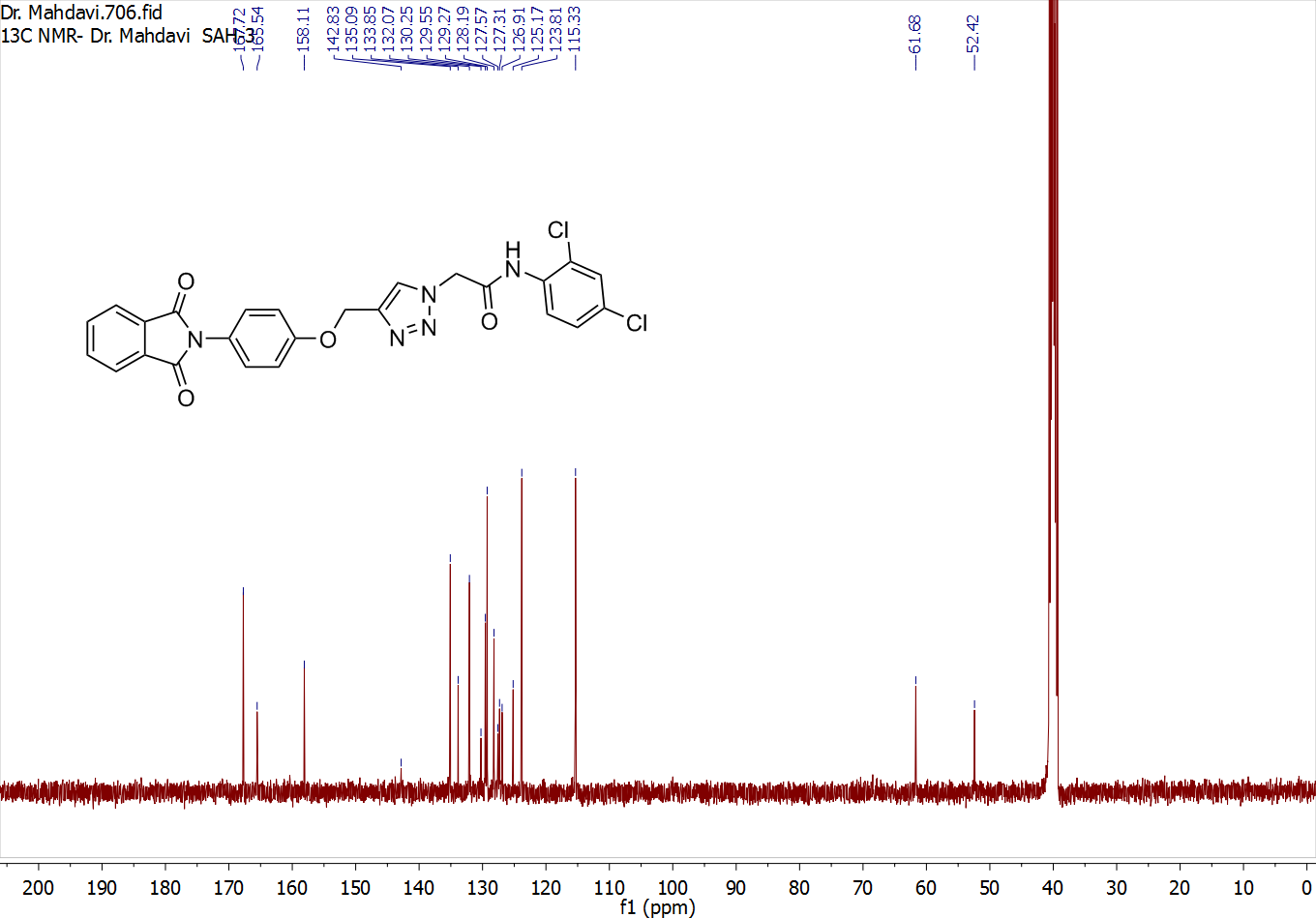
**

**
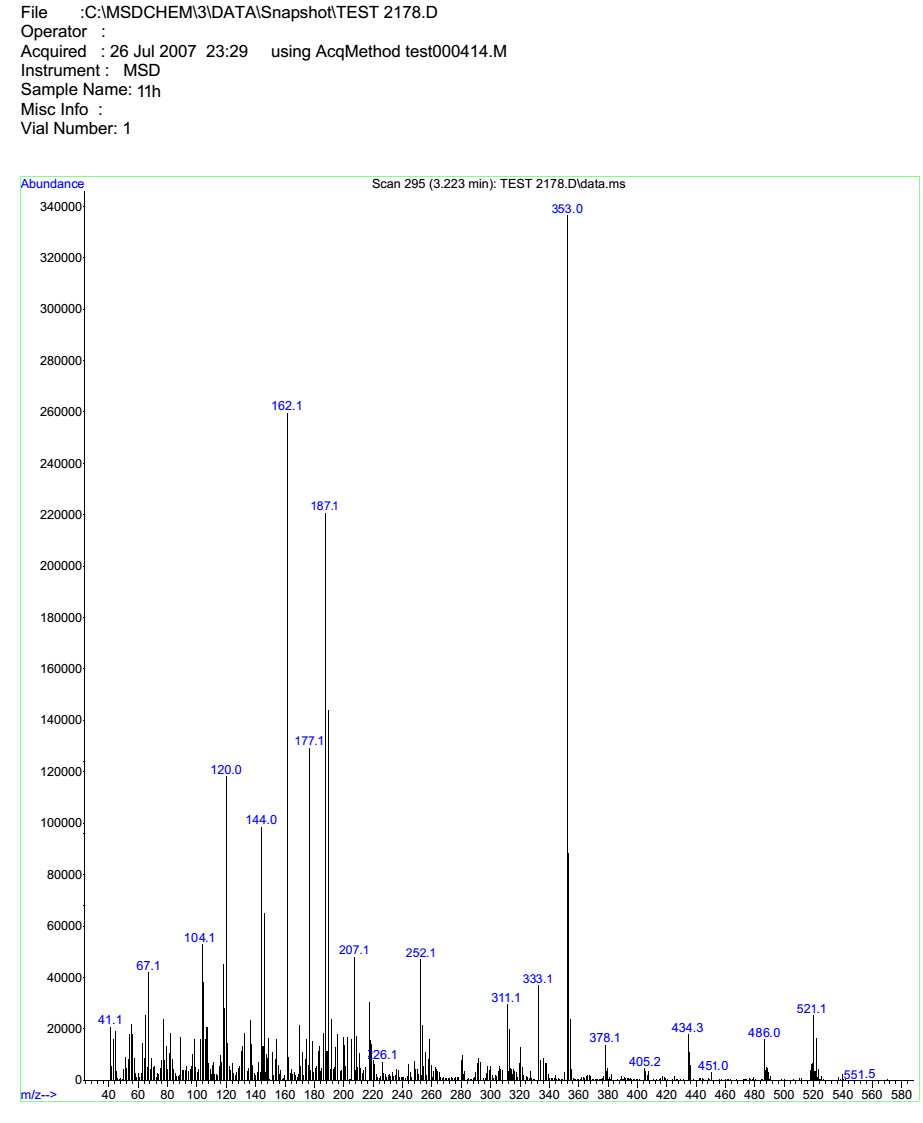
**

***N*-(2,6-dichlorophenyl)-2-(4-((4-(1,3-dioxoisoindolin-2-yl)phenoxy)methyl)-1*H*-1,2,3-triazol-1-yl)acetamide (11i)**


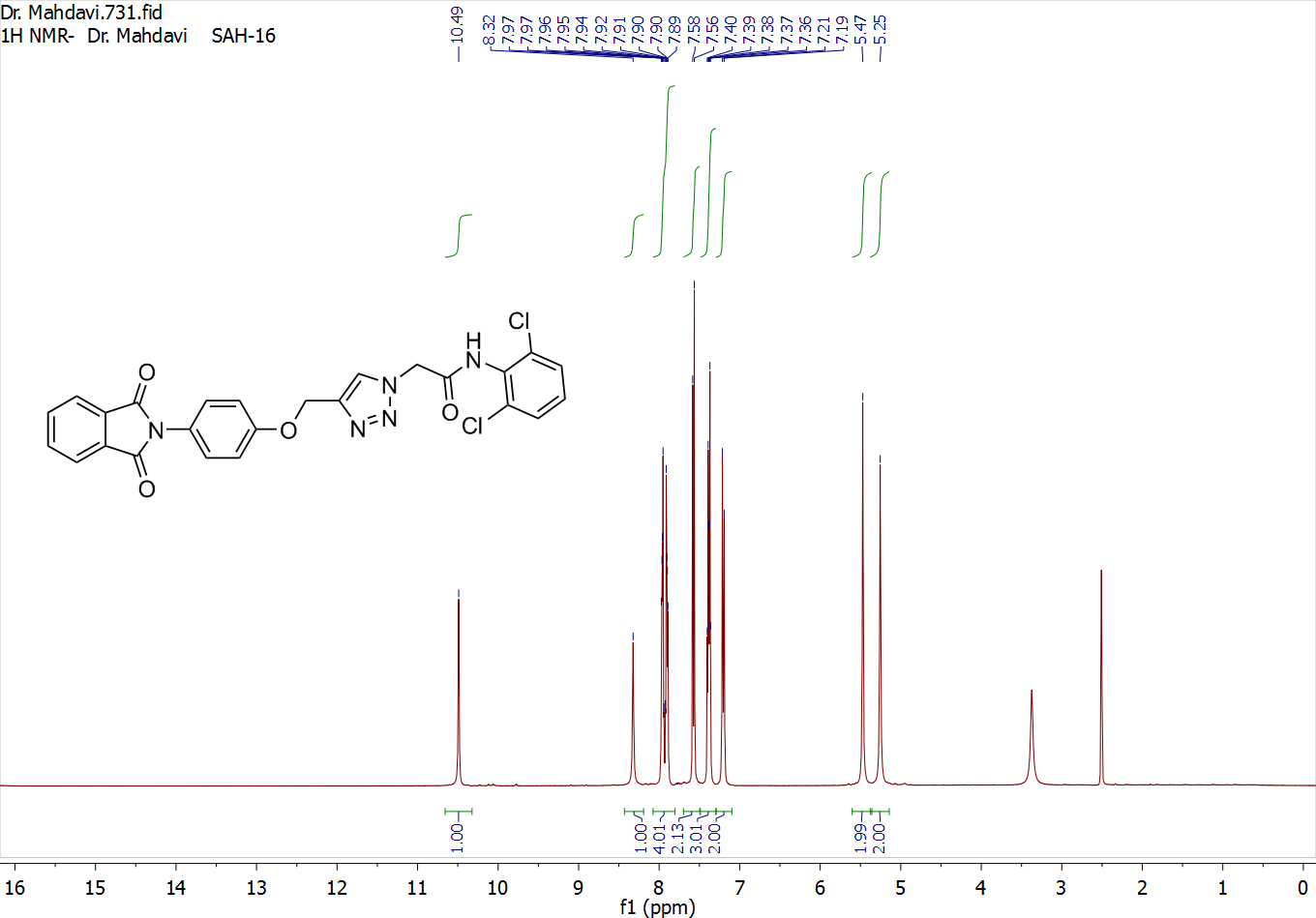


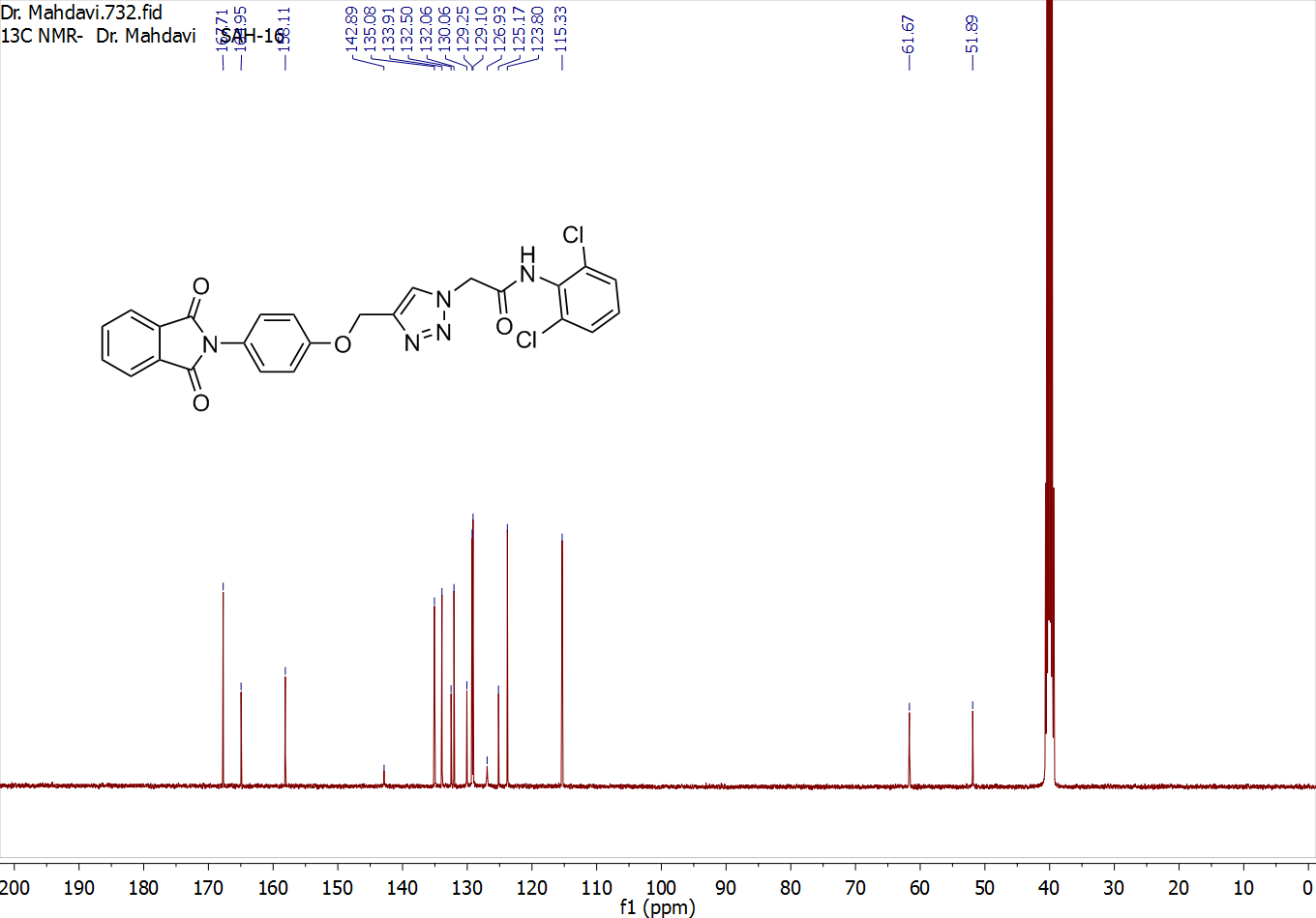


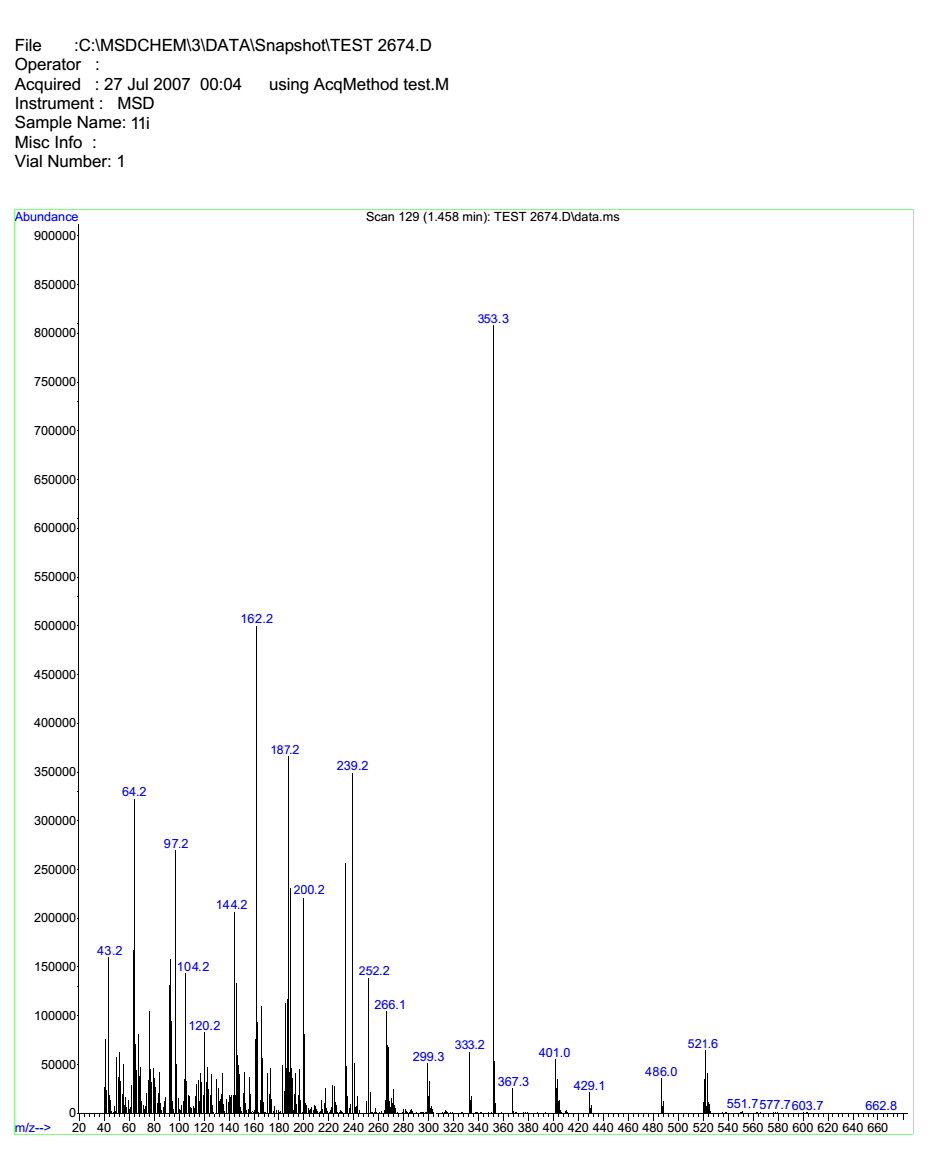


***N*-(4-bromophenyl)-2-(4-((4-(1,3-dioxoisoindolin-2-yl)phenoxy)methyl)-1*H*-1,2,3-triazol-1-yl)acetamide (11j)**


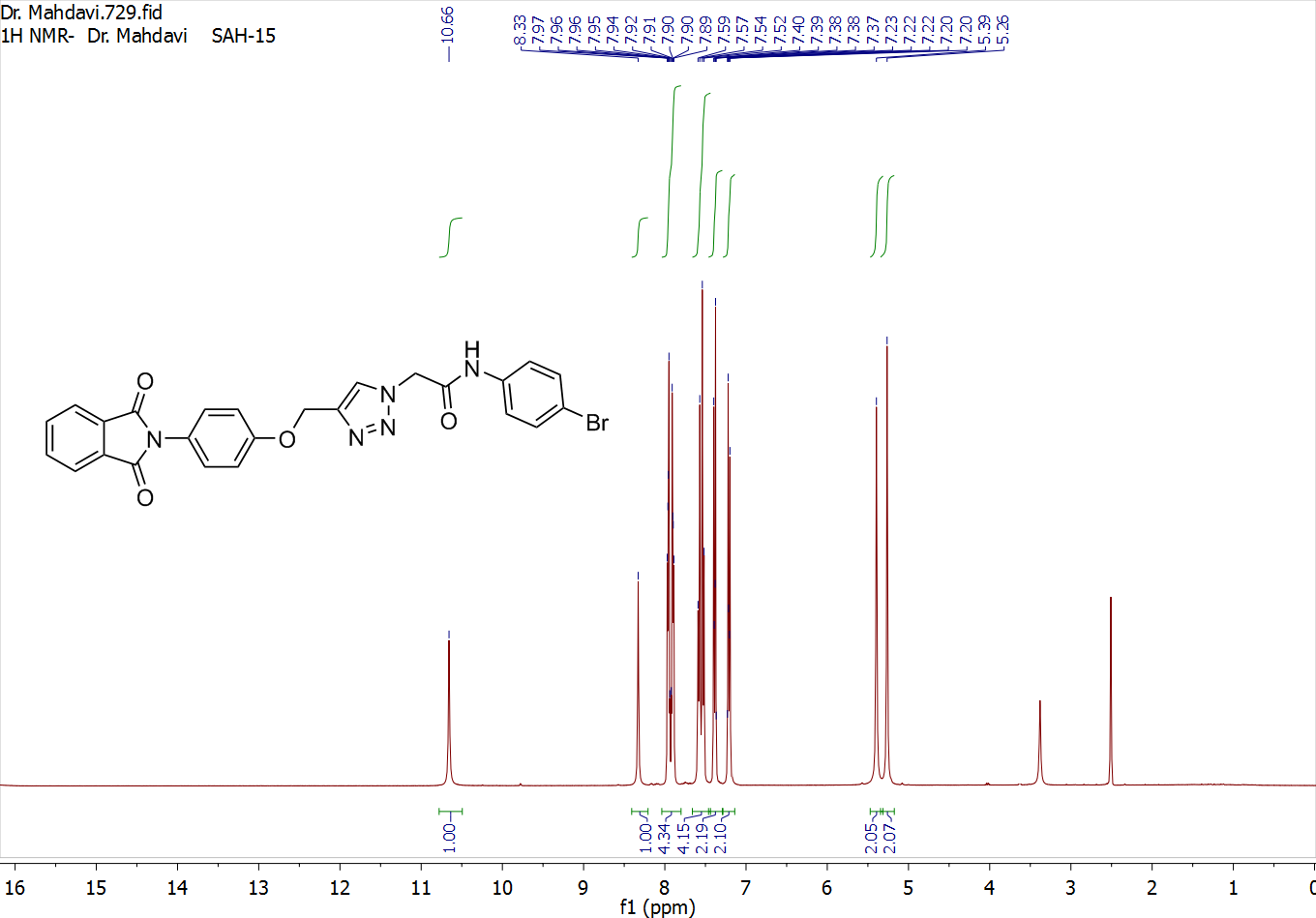


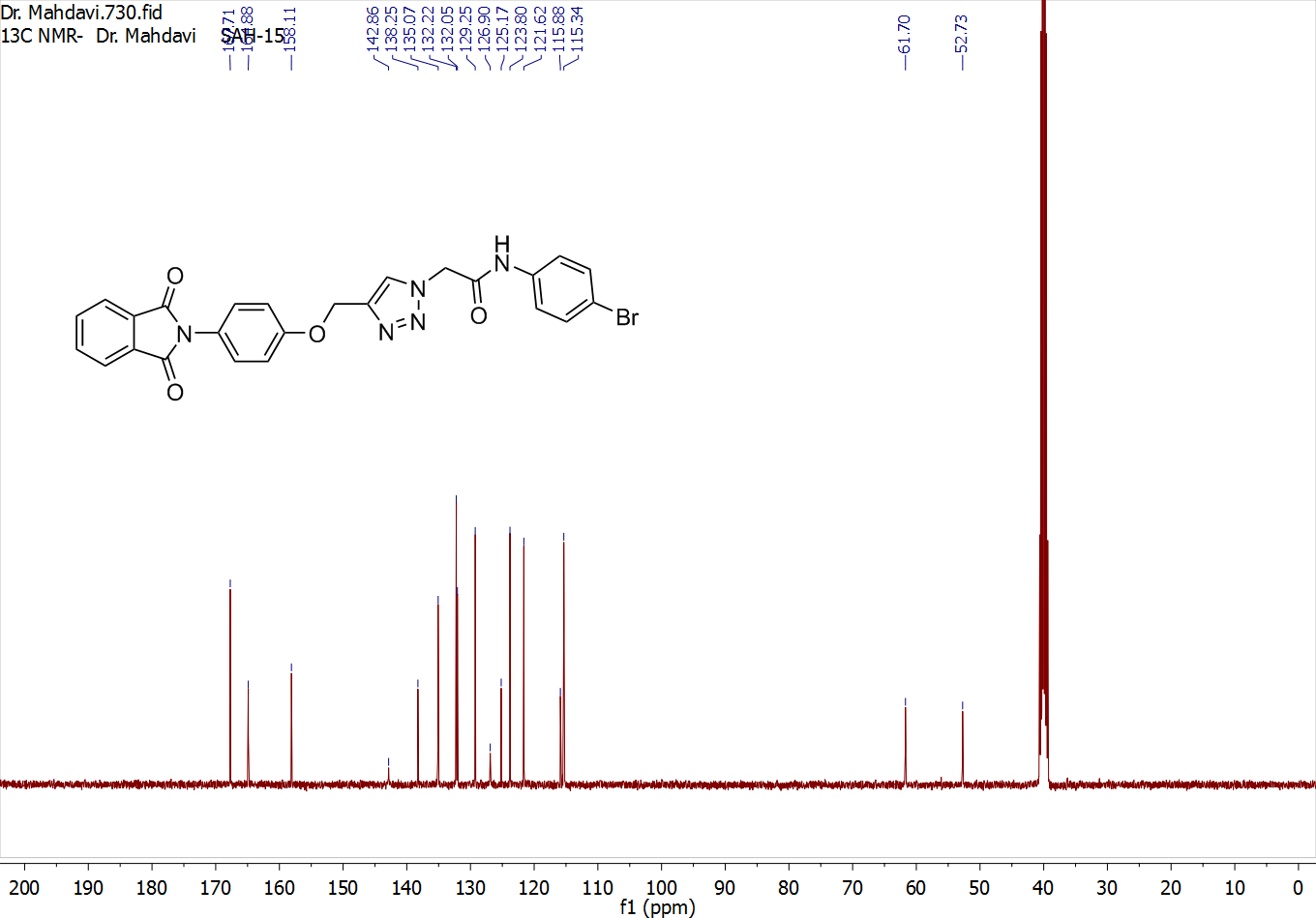


**
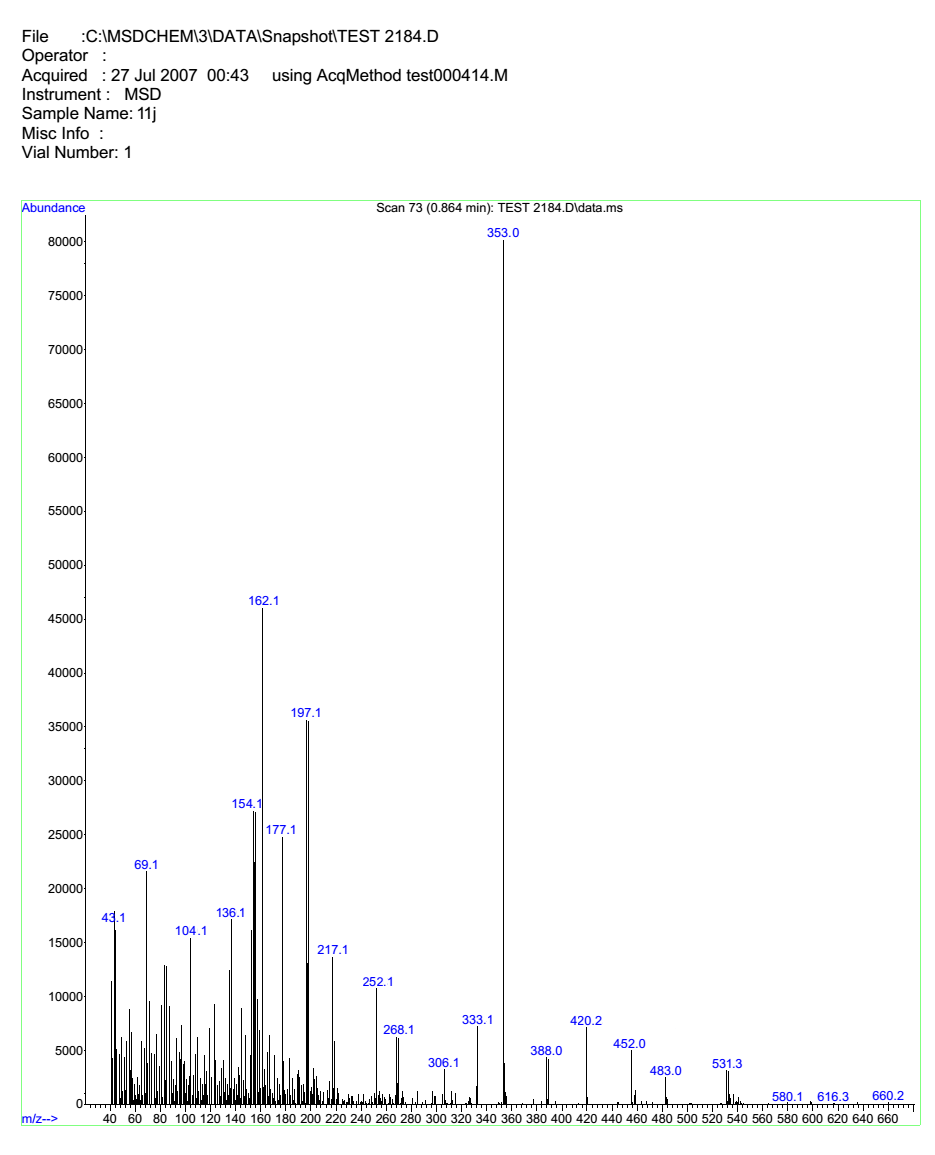
**

**2-(4-((4-(1,3-dioxoisoindolin-2-yl)phenoxy)methyl)-1*H*-1,2,3-triazol-1-yl)-*N*-(4-nitrophenyl)acetamide (11k)**

**
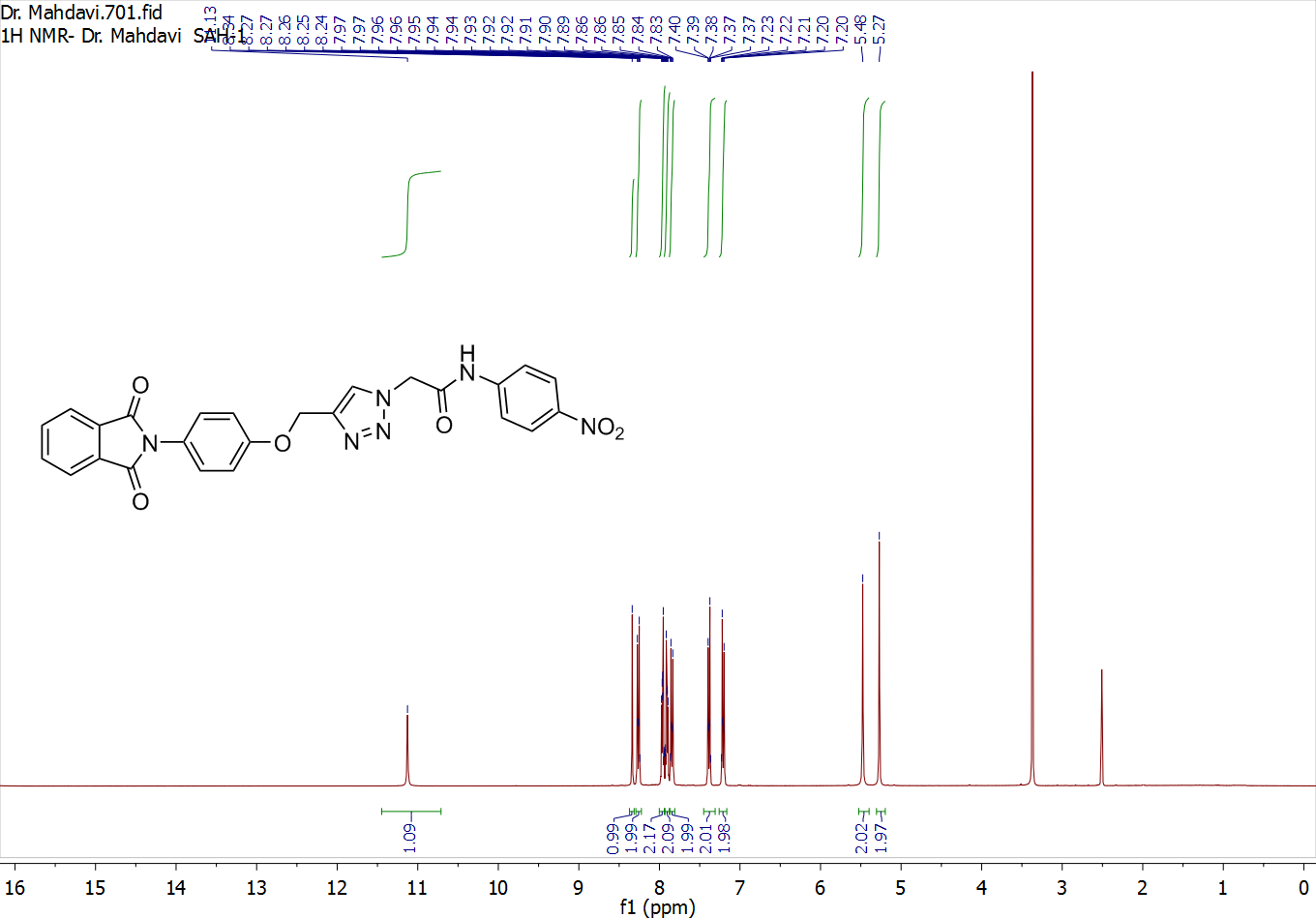
**

**
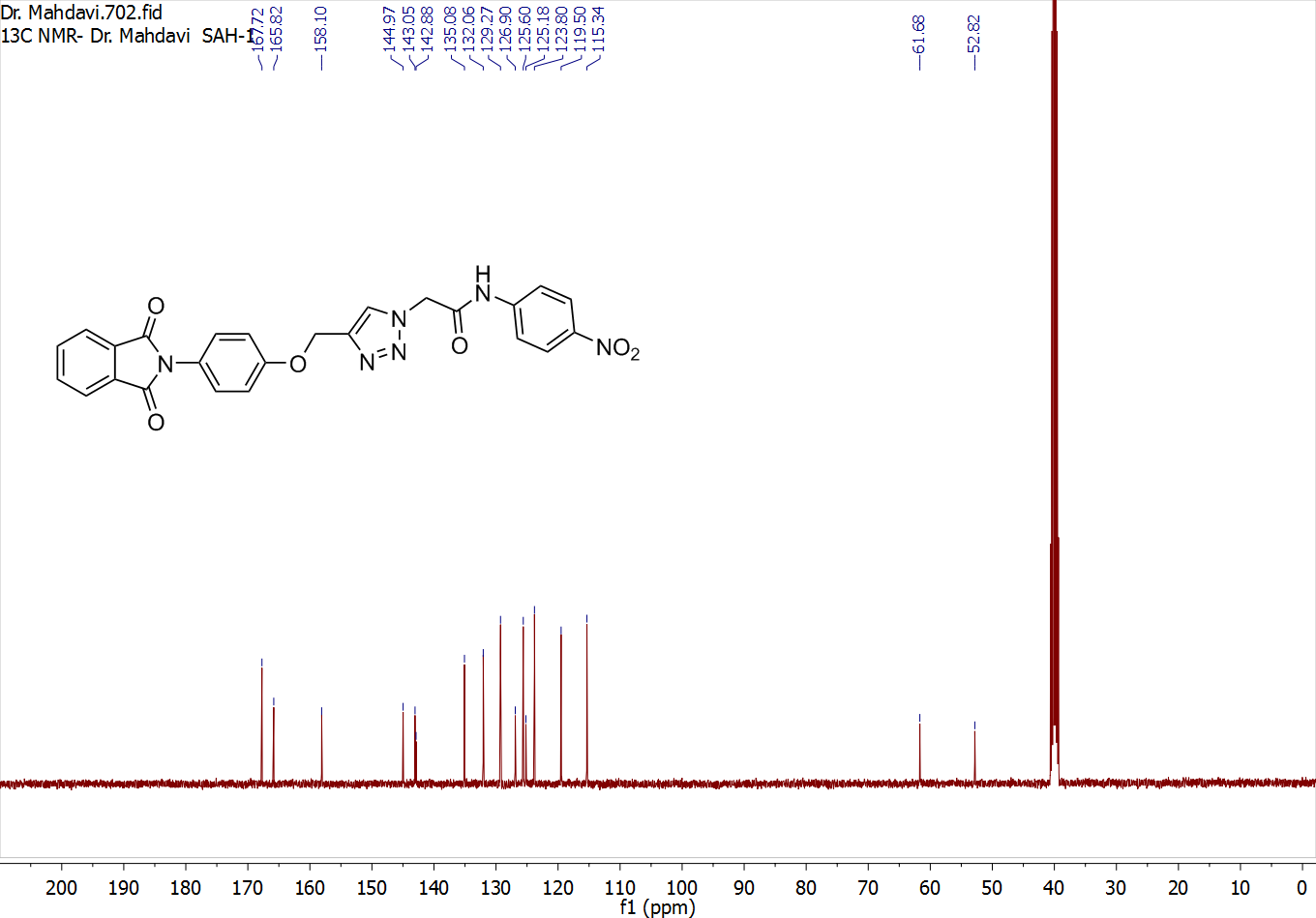
**

**
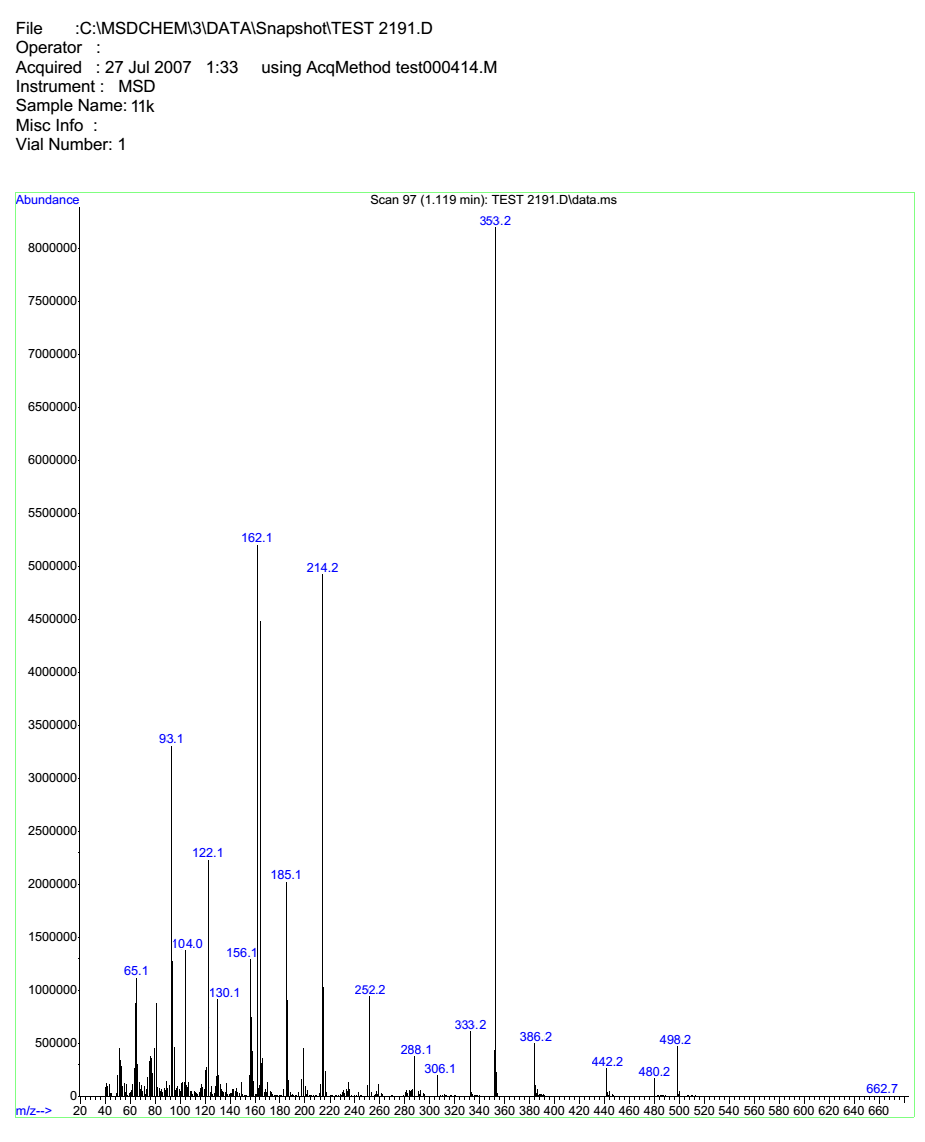
**

**2-(4-((4-(1,3-dioxoisoindolin-2-yl)phenoxy)methyl)-1*H*-1,2,3-triazol-1-yl)-*N*-(2-methyl-4-nitrophenyl)acetamide (11l)**


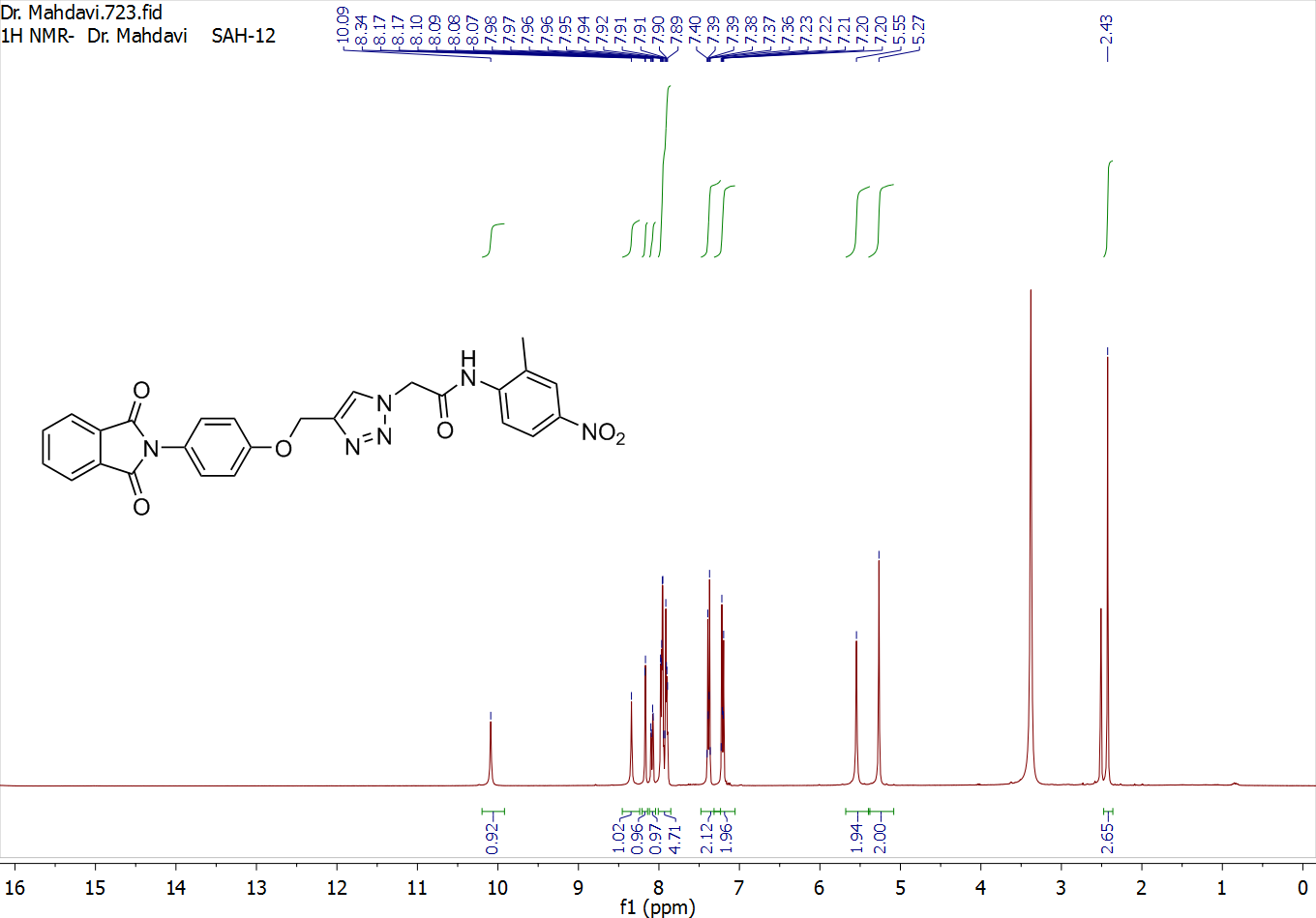


**
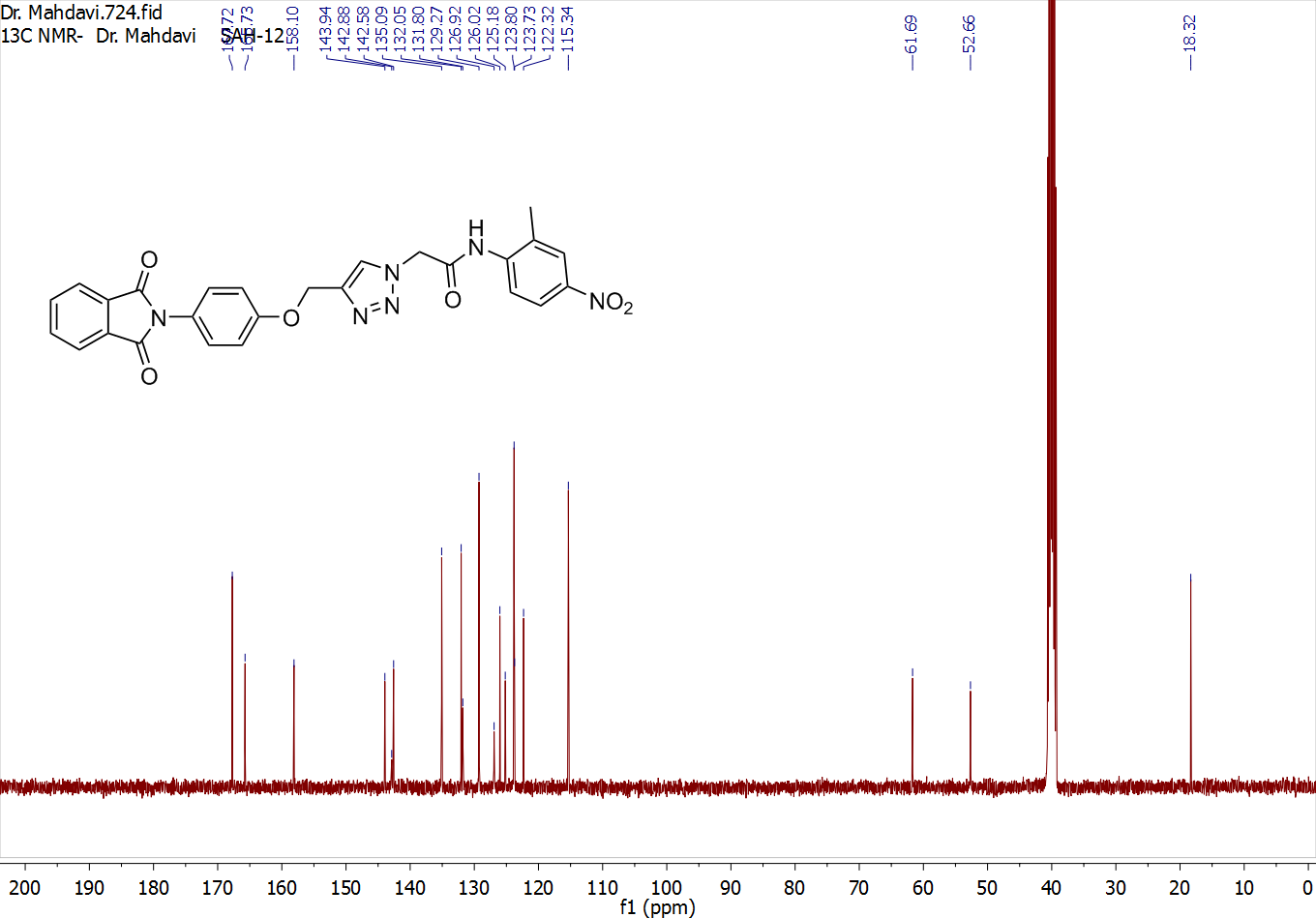
**

**
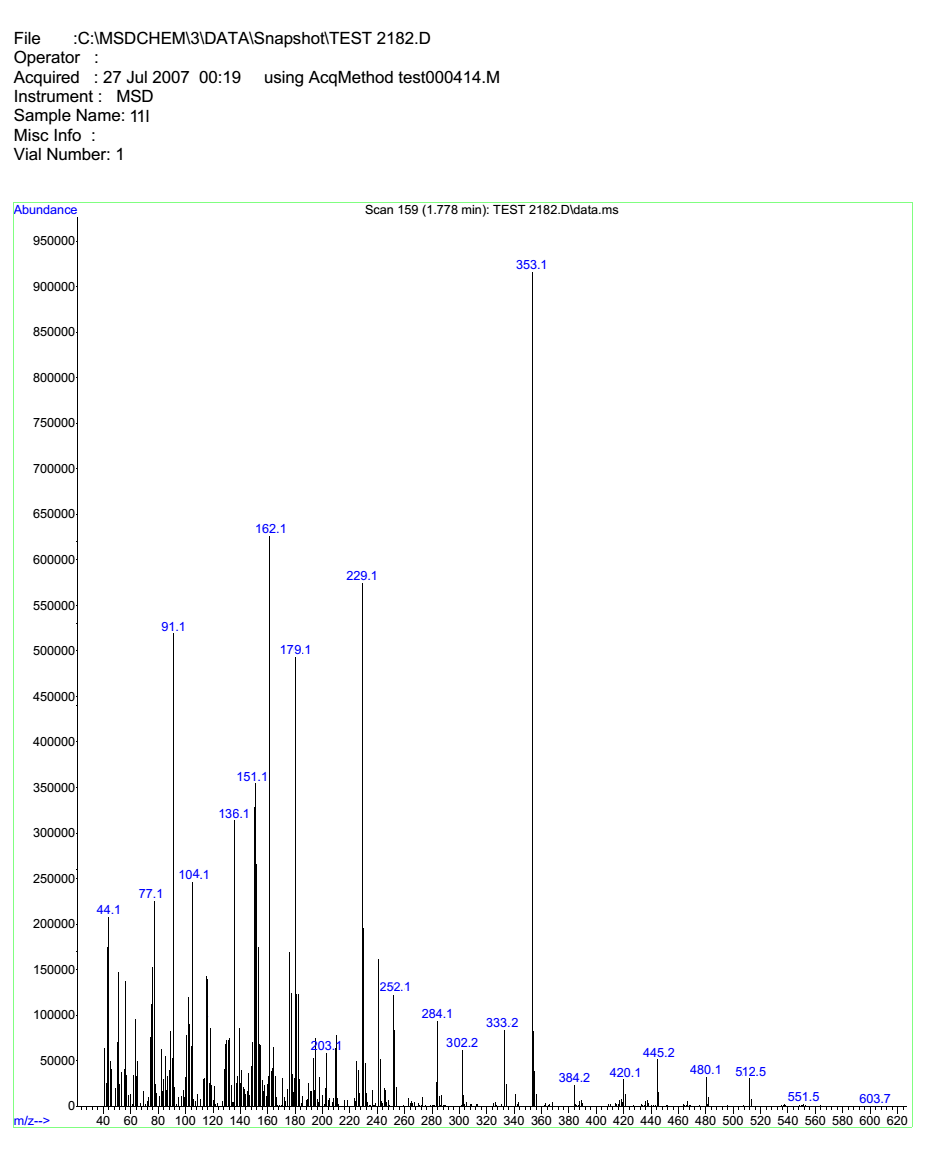
**

***N*-benzyl-2-(4-((4-(1,3-dioxoisoindolin-2-yl)phenoxy)methyl)-1*H*-1,2,3-triazol-1-yl)acetamide (11m)**


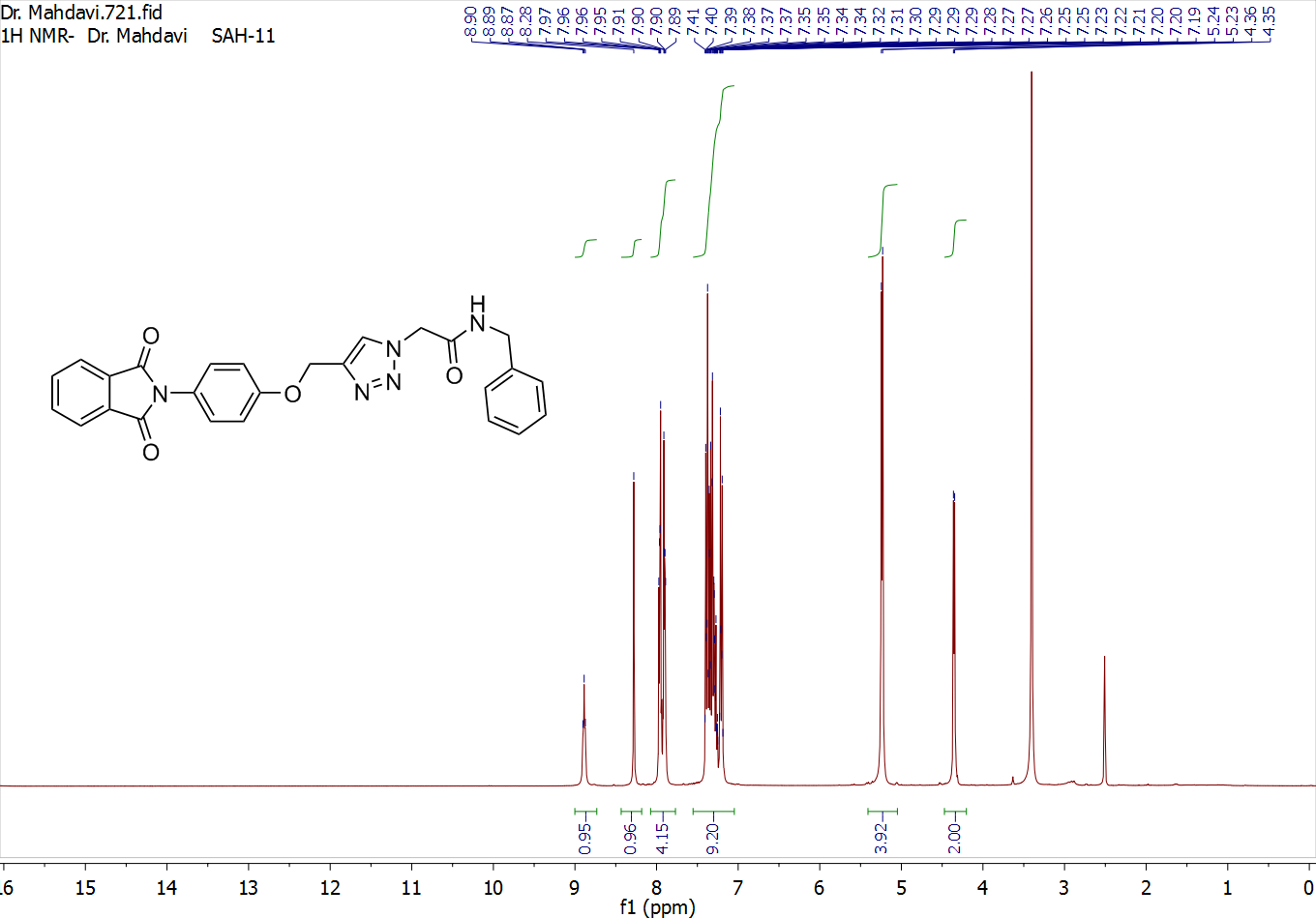


**
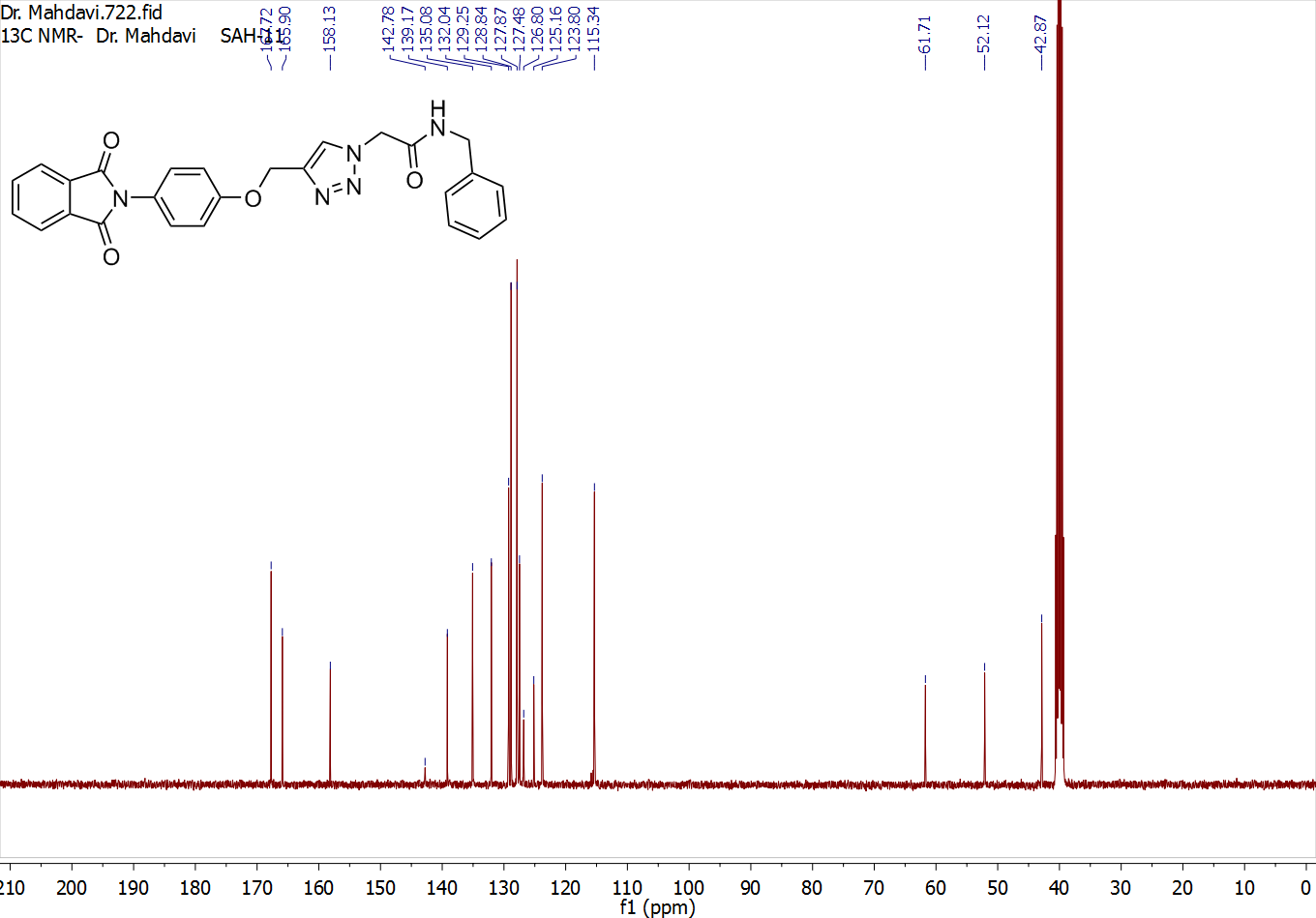
**

**
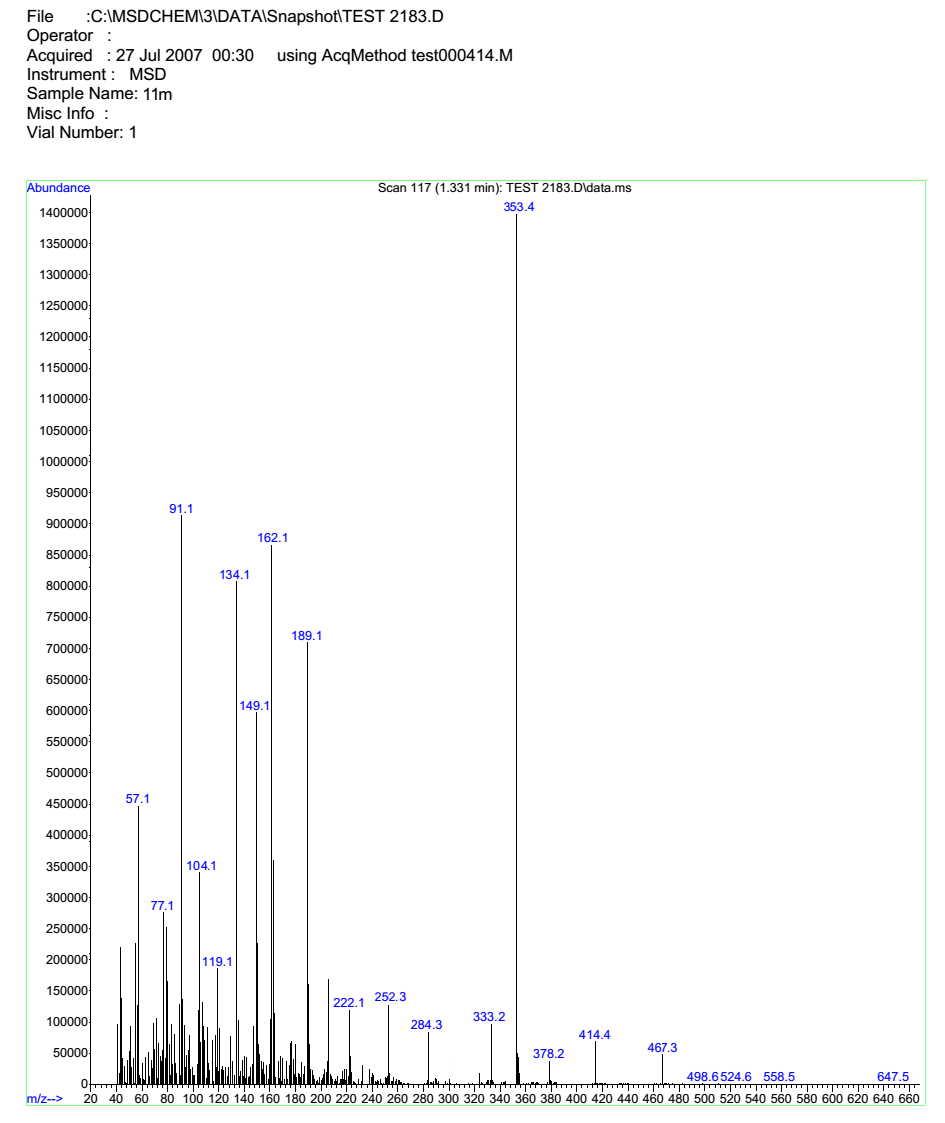
**

**2-(4-((4-(1,3-dioxoisoindolin-2-yl)phenoxy)methyl)-1*H*-1,2,3-triazol-1-yl)-*N*-(4-fluorobenzyl)acetamide (11n)**


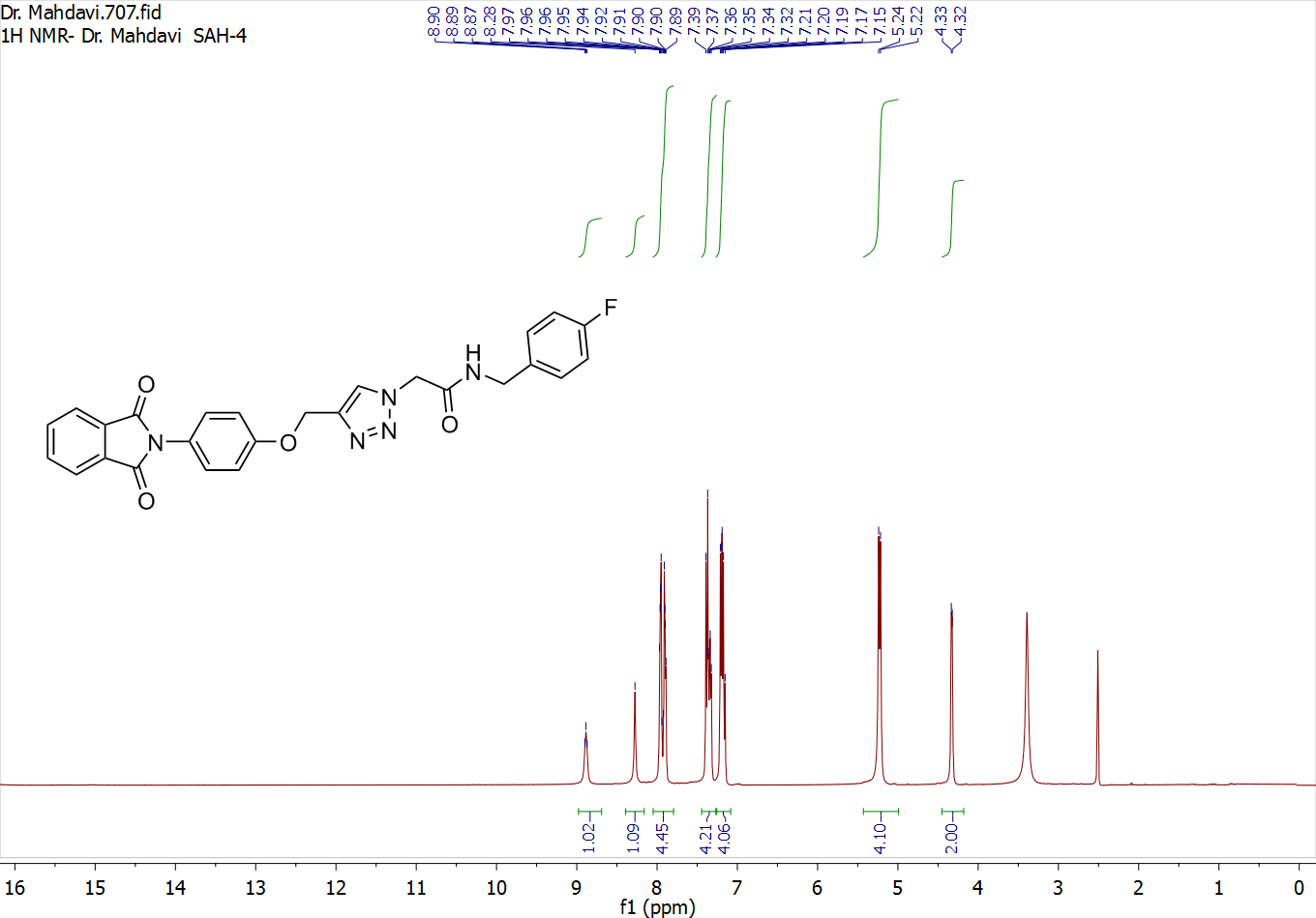


**
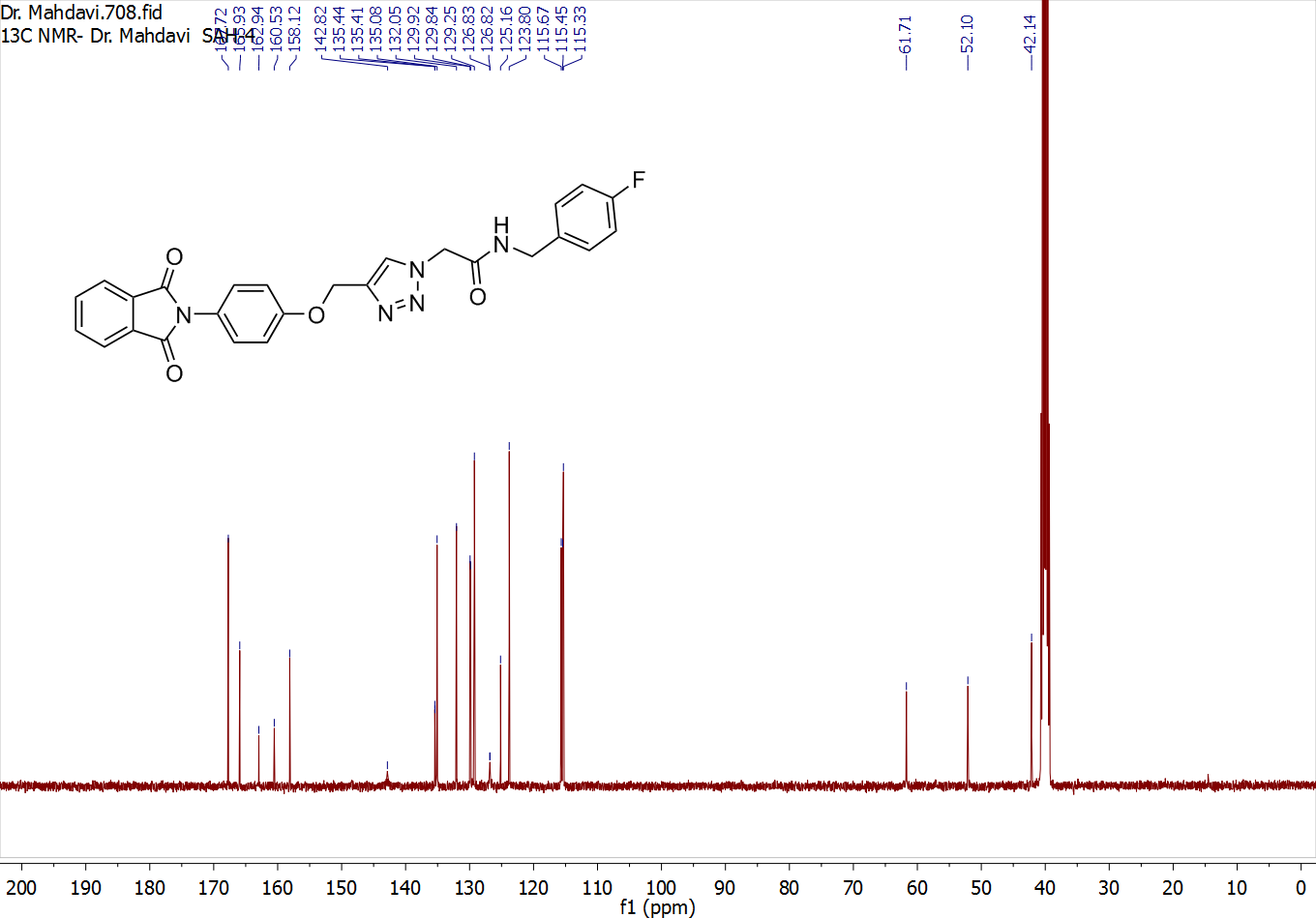
**

**
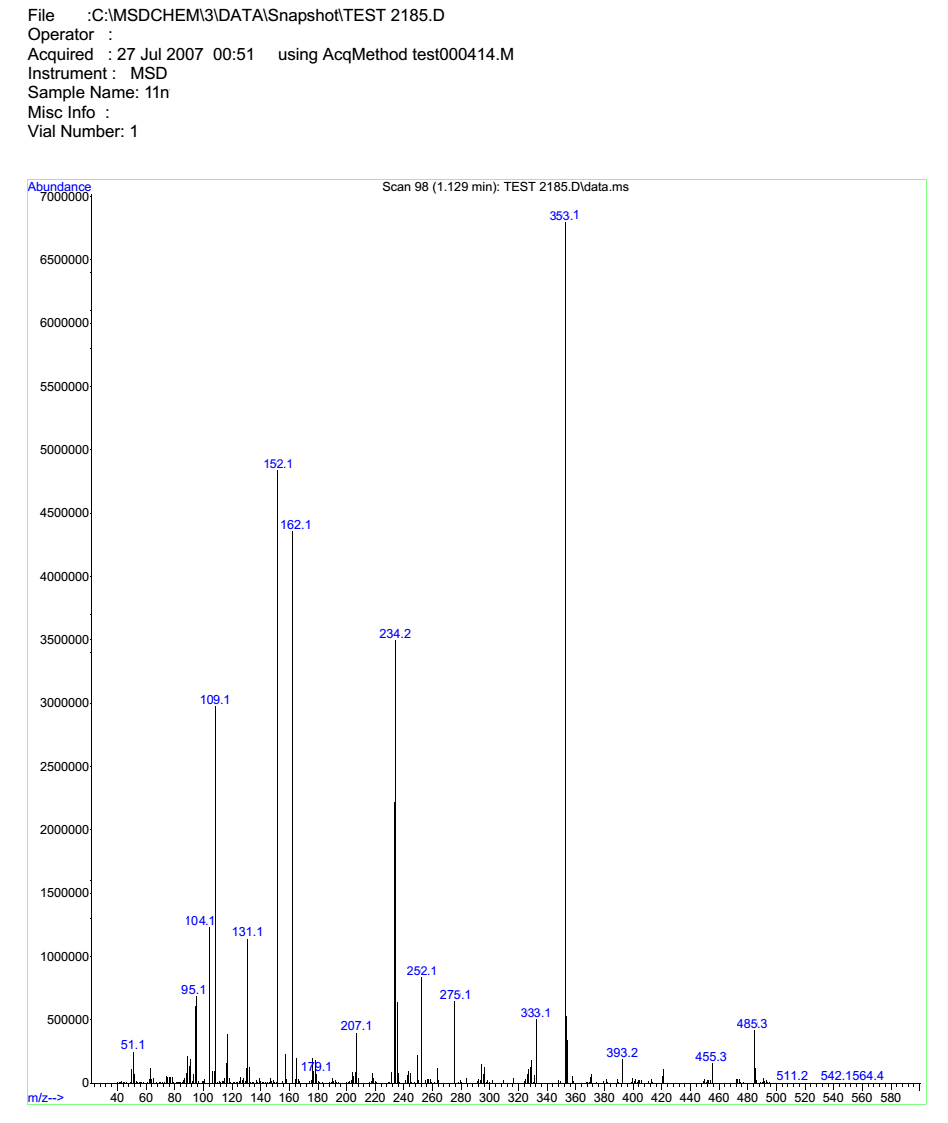
**
